# Supplementary material for: An empirical evaluation of link quality utilization in ETX routing for VANETs
Source: PeerJ Comput Sci. 2024 Sep 6;10:e2259. doi: 10.7717/peerj-cs.2259 (PMC11419655; doi:10.7717/peerj-cs.2259)
Supplement: Supplemental Information 1 [file peerj-cs-10-2259-s001.bz2 › ns-allinone-3.29/ns-3.29/CHANGES.html]

ns-3 Change Log


# ns-3: API and model change history

ns-3 is an evolving system and there will be API or behavioral changes
from time to time. Users who try to use scripts or models across
versions of ns-3 may encounter problems at compile time, run time, or
may see the simulation output change.

We have adopted the development policy that we are going to try to ease
the impact of these changes on users by documenting these changes in a
single place (this file), and not by providing a temporary or permanent
backward-compatibility software layer.

A related file is the RELEASE\_NOTES file in the top level directory.
This file complements RELEASE\_NOTES by focusing on API and behavioral
changes that users upgrading from one release to the next may encounter.
RELEASE\_NOTES attempts to comprehensively list all of the changes
that were made. There is generally some overlap in the information
contained in RELEASE\_NOTES and this file.

The goal is that users who encounter a problem when trying to use older
code with newer code should be able to consult this file to find
guidance as to how to fix the problem. For instance, if a method name
or signature has changed, it should be stated what the new replacement
name is.

Note that users who upgrade the simulator across versions, or who work
directly out of the development tree, may find that simulation output
changes even when the compilation doesn't break, such as when a
simulator default value is changed. Therefore, it is good practice for
\_anyone\_ using code across multiple ns-3 releases to consult this file,
as well as the RELEASE\_NOTES, to understand what has changed over time.

This file is a best-effort approach to solving this issue; we will do
our best but can guarantee that there will be things that fall through
the cracks, unfortunately. If you, as a user, can suggest improvements
to this file based on your experience, please contribute a patch or drop
us a note on ns-developers mailing list.

---

# Changes from ns-3.28 to ns-3.29

## New API:

- CommandLine can now handle non-option (positional) arguments.
- Added CommandLine::Parse (const std::vector> args)
- NS\_LOG\_FUNCTION can now log the contents of vectors
- A new position allocator has been added to the buildings module, allowing
  nodes to be placed outside of buildings defined in the scenario.
- The Hash() method has been added to the QueueDiscItem class to compute the
  hash of various fields of the packet header (depending on the packet type).
- Added a priority queue disc (PrioQueueDisc).
- Added 3GPP HTTP model- Added TCP PRR as recovery algorithm- Added a new trace source in StaWifiMac for tracing beacon arrivals
    - Added a new helper method to ApplicationContainer to start applications with some jitter around the start time
    - (network) Add a method to check whether a node with a given ID is within a NodeContainer.

## Changes to existing API:

- TrafficControlHelper::Install now only includes root queue discs in the returned
  QueueDiscContainer.
- Recovery algorithms are now in a different class, instead of being tied to TcpSocketBase.
  Take a look to TcpRecoveryOps for more information.
- The Mode, MaxPackets and MaxBytes attributes of the Queue class, that had been deprecated in favor of the MaxSize attribute in ns-3.28, have now been removed and cannot be used anymore. Likewise, the methods to get/set the old attributes have been removed as well. Commands such as:

  ```
    Config::SetDefault ("ns3::QueueBase::MaxPackets", UintegerValue (4));
  ```

  should now be written as:

  ```
    Config::SetDefault ("ns3::QueueBase::MaxSize", QueueSizeValue (QueueSize (QueueSizeUnit::PACKETS, 4)));
  ```

  or with a string value with 'b' (bytes) or 'p' (packets) suffix, such as:

  ```
    Config::SetDefault ("ns3::QueueBase::MaxSize", StringValue ("4p"));
  ```
- The Limit attribute of the PfifoFastQueueDisc class, that had been deprecated in favor of the MaxSize attribute in ns-3.28, has now been removed and cannot be used anymore. Likewise, the methods to get/set the old Limit attribute have been removed as well. The GetMaxSize/SetMaxSize methods of the base QueueDisc class must be used instead.
- The Mode, MaxPackets and MaxBytes attributes of the CoDelQueueDisc class, that had been deprecated in favor of the MaxSize attribute in ns-3.28, have now been removed and cannot be used anymore. Likewise, the methods to get/set the old attributes have been removed as well. The GetMaxSize/SetMaxSize methods of the base QueueDisc class must be used instead.
- The PacketLimit attribute of the FqCoDelQueueDisc class, that had been deprecated in favor of the MaxSize attribute in ns-3.28, has now been removed and cannot be used anymore. Likewise, the methods to get/set the old PacketLimit attribute have been removed as well. The GetMaxSize/SetMaxSize methods of the base QueueDisc class must be used instead.
- The Mode and QueueLimit attributes of the PieQueueDisc class, that had been deprecated in favor of the MaxSize attribute in ns-3.28, have now been removed and cannot be used anymore. Likewise, the methods to get/set the old attributes have been removed as well. The GetMaxSize/SetMaxSize methods of the base QueueDisc class must be used instead.
- The Mode and QueueLimit attributes of the RedQueueDisc class, that had been deprecated in favor of the MaxSize attribute in ns-3.28, have now been removed and cannot be used anymore. Likewise, the methods to get/set the old attributes have been removed as well. The GetMaxSize/SetMaxSize methods of the base QueueDisc class must be used instead.
- Several traffic generating applications have additional trace sources that export not only the transmitted or received packet but also the source and destination addresses.
- The returned type of **GetNDevices** methods in **Channel** and subclasses derived from it were changed from uint32\_t to std::size\_t. Likewise, the input parameter type of **GetDevice** in **Channel** and its subclasses were changed from uint32\_t to std::size\_t.
- Wifi classes **DcfManager**, **DcaTxop** and **EdcaTxopN** were renamed to **ChannelAccessManager**, **Txop** and **QosTxop**, respectively.
- QueueDisc::DequeuePeeked has been merged into QueueDisc::Dequeue and hence no longer exists.
- The QueueDisc base class now provides a default implementation of the DoPeek private method
  based on the QueueDisc::PeekDequeue method, which is now no longer available.
- The QueueDisc::SojournTime trace source is changed from a TracedValue to a TracedCallback; callbacks that hook this trace must provide one ns3::Time argument, not two.
- To avoid the code duplication in SingleModelSpectrumChannel and MultiModelSpectrumChannel classes, the attributes MaxLossDb and PropagationLossModel, and the traces PathLoss and TxSigParams are moved to the base class SpectrumChannel. Similarly, the functions AddPropagationLossModel, AddSpectrumPropagationLossModel, SetPropagationDelayModel and GetSpectrumPropagationLossModel are now defined in SpectrumChannel class. Moreover, the TracedCallback signature of LossTracedCallback has been updated from :

  ```
     typedef void (* LossTracedCallback) (Ptr<SpectrumPhy> txPhy, Ptr<SpectrumPhy> rxPhy, double lossDb);
  ```

  To :

  ```
     typedef void (* LossTracedCallback) (Ptr<const SpectrumPhy> txPhy, Ptr<const SpectrumPhy> rxPhy, double lossDb);
  ```
- For the sake of LTE module API consistency the IPV6 related functions AssignUeIpv6Address and GetUeDefaultGatewayAddress6 are now declared in EpcHelper base class. Thus, these functions are now declared as virtual in the child classes, i.e., EmuEpcHelper and PointToPointEpcHelper.

## Changes to build system:

- Waf upgraded from 1.8.19 to 2.0.9, and ns-3 build scripts aligned to the new API.
- The '--no32bit-scan' argument is removed from Waf apiscan; generation of ILP32 bindings is now automated from the LP64 bindings.
- When using on newer compilers, new warnings may trigger build failures.
  The --disable-werror flag can be passed to Waf at configuration time to turn
  off the Werror behavior.
- GTK+3 libraries (including PyGObject, GooCanvas2) are needed for the Pyviz visualizer, replacing GTK+2 libraries.

## Changed behavior:

- FqCoDelQueueDisc now computes the hash of the packet's 5-tuple to determine
  the flow the packet belongs to, unless a packet filter has been configured.
  The previous behavior is simply obtained by not configuring any packet filter.
  Consequently, the FqCoDelIpv{4,6}PacketFilter classes have been removed.
- ARP packets now pass through the traffic control layer, as in Linux.
- The maximum size UDP packet of the UdpClient application is no longer limited to 1500 bytes.
- The default values of the **MaxSlrc** and **FragmentationThreshold** attributes in WifiRemoteStationManager were changed from 7 to 4 and from 2346 to 65535, respectively.

---

# Changes from ns-3.27 to ns-3.28

## New API:

- When deserializing Packet contents, **Header::Deserialize (Buffer::Iterator start)** and **Trailer::Deserialize (Buffer::Iterator start)** can not successfully deserialize variable-length headers and trailers. New variants of these methods that also include an 'end' parameter are now provided.
- Ipv[4,6]AddressGenerator can now check if an address is allocated (**Ipv[4,6]AddressGenerator::IsAddressAllocated**) or a network has some allocated address (**Ipv[4,6]AddressGenerator::IsNetworkAllocated**).
- LTE UEs can now use IPv6 to send and receive traffic.
- UAN module now supports an IP stack.
- Class **TcpSocketBase** trace source *CongestionWindowInflated* shows the values with the in-recovery inflation and the post-recovery deflation.- Added a FIFO queue disc (FifoQueueDisc) and the Token Bucket Filter (TbfQueueDisc).

## Changes to existing API:

- Class **LrWpanMac** now supports extended addressing mode. Both **McpsDataRequest** and **PdDataIndication** methods will now use extended addressing if **McpsDataRequestParams::m\_srcAddrMode** or **McpsDataRequestParams::m\_dstAddrMode** are set to **EXT\_ADDR**.
- Class **LteUeNetDevice** MAC address is now a 64-bit address and can be set during construction.
- Class **TcpSocketBase** trace source *CongestionWindow* shows the values without the in-recovery inflation and the post-recovery deflation; the old behavior has been moved to the new trace source *CongestionWindowInflated*.- The Mode, MaxPackets and MaxBytes attributes of the Queue class have been deprecated in favor of the MaxSize attribute. Old attributes can still be used, but using them will be no longer possible in one of the next releases. The methods to get/set the old attributes will be removed as well.
  - The attributes of the QueueDisc subclasses that separately determine the mode and the limit of the QueueDisc have been deprecated in favor of the single MaxSize attribute.
  - The GetQueueSize method of some QueueDisc subclasses (e.g., RED) has been removed and replaced by the GetCurrentSize method of the QueueDisc base class.

## Changes to build system:

- The C++ standard used during compilation (default std=c++11) can be now be changed via the CXXFLAGS variable.

## Changed behavior:

- All Wi-Fi management frames are now transmitted using the lowest basic rate.
- The Wi-Fi spectrum model now takes into account adjacent channels through OFDM transmit spectrum masks.
- The CsmaNetDevice::PhyTxBeginTrace will trace all attempts to transmit, even those that result in drops. Previously, eventual channel drops were excluded from this trace.- The TCP congestion window trace now does not report on window inflation during fast recovery phase because it is no longer internally maintained as an inflated value (a separate trace called CongestionWindowInflated can be used to recover the old trace behavior).

---

# Changes from ns-3.26 to ns-3.27

## New API:

- Added `Vector{2,3}D.GetLength ()`.
- Overloaded `operator+` and `operator-` for `Vector{2,3}D`.
- Added iterator version of WifiHelper::Install() to install Wi-Fi devices on range of nodes.
- Added a new attribute in TcpSocketBase to track the advertised window.
- Included the model of **TCP Ledbat**.
- Included the TCP SACK-based loss recovery algorithm outlined in RFC 6675.
- Added **TCP SACK** and the **SACK emulation**. Added an Attribute to TcpSocketBase class,
  called "Sack", to enable or disable the SACK option usage.
- In 'src/wifi', several changes were made to enable partial **802.11ax** High Efficiency (HE) support:
  - A new standard value has been added that enables the new 11ax data rates.
  - A new 11ax preamble has been added.
  - A new attribute was added to configure the guard interval duration for High Efficiency (HE) PHY entities. This attribute can be set using the YansWifiPhyHelper.
  - A new information element has been added: HeCapabilities. This information element is added to the MAC frame header if the node is a HE node. This HeCapabilites information element is used to advertise the HE capabilities of the node to other nodes in the network.
- A new class were added for the RRPAA WiFi rate control mechanism.
- Included carrier aggregation feature in LTE module

- LTE model is extended to support carrier aggregation feature according to 3GPP Release 10, for up to 5 component
  carriers.
- InstallSingleEnbDevice and InstalSingeUeDevice functions of LteHelper are now constructing LteEnbDevice and LteUeDevice
  according to CA architecture. Each device, UE and eNodeB contains an instance of component carrier manager, and may
  have several component carrier instances.
- SAP interfaces are extended to include CA message exchange functionality.
- RRC connection procedure is extended to allow RRC connection reconfiguration for the configuration of the secondary carriers.
- RRC measurement reporting is extended to allow measurement reporting from the secondary carriers.
- LTE traces are extended to include component carrier id.

- Function **PrintRoutingTable** has been extended to add an optional Time::Units
  parameter to specify the time units used on the report. The new parameter is
  optional and if not specified defaults to the previous behavior (Time::S).
- **TxopTrace**: new trace source exported by EdcaTxopN.
- A **GetDscpCounts** method is added to **Ipv4FlowClassifier** and **Ipv6FlowClassifier**
  which returns a vector of pairs (dscp,count), each of which indicates how many packets with the
  associated dscp value have been classified for a given flow.
- MqQueueDisc, a multi-queue aware queue disc modelled after the mq qdisc in Linux, has been introduced.
- Two new methods, **QueueDisc::DropBeforeEnqueue()** and **QueueDisc::DropAfterDequeue()** have
  been introduced to replace **QueueDisc::Drop()**. These new methods require the caller to specify the
  reason why a packet was dropped. Correspondingly, two new trace sources ("DropBeforeEnqueue" and
  "DropAfterDequeue") have been added to the QueueDisc class, providing both the items that were dropped
  and the reason why they were dropped.
- Added **QueueDisc::GetStats()** which returns detailed statistics about the operations of
  a queue disc. Statistics can be accessed through the member variables of the returned object and
  by calling the **GetNDroppedPackets()**, **GetNDroppedBytes()**, **GetNMarkedPackets()** and **GetNMarkedBytes()** methods on the returned object. Such methods return the number of packets/bytes
  dropped/marked for the specified reason (passed as argument). Consequently:
  - A number of methods of the QueueDisc class have been removed: **GetTotalReceivedPackets()**,
    **GetTotalReceivedBytes()**, **GetTotalDroppedPackets()**, **GetTotalDroppedBytes()**,
    **GetTotalRequeuedPackets()**, **GetTotalRequeuedBytes()**.
  - The **Stats** struct and the **GetStats()** method of **RedQueueDisc** and **PieQueueDisc** have been removed and replaced by those of the QueueDisc base class.
  - The **GetDropOverLimit** and **GetDropCount** methods of **CoDelQueueDisc** have been removed.
    The values they returned can be obtained by calling, respectively,
    GetStats ().GetNDroppedPackets (CoDelQueueDisc::OVERLIMIT\_DROP) and
    GetStats ().GetNDroppedPackets (CoDelQueueDisc::TARGET\_EXCEEDED\_DROP). The "DropCount" trace of
    **CoDelQueueDisc** has been removed as well. Packets dropped because the target is exceeded can
    be obtained through the new "DropAfterDequeue" trace of the QueueDisc class.
- The new **QueueDisc::Mark()** method has been introduced to allow subclasses to request to mark a packet.
  The caller must specify the reason why the packet must be marked. Correspondingly, a new trace source ("Mark")
  has been added to the QueueDisc class, providing both the items that were marked and the reason why they
  were marked.
- A new trace source, **SojournTime**, is exported by the QueueDisc base class to provide the
  sojourn time of every packet dequeued from a queue disc. This has been made possible by adding a
  timestamp to QueueDiscItem objects, which can be set/get through the new **GetTimeStamp()** and
  **SetTimeStamp()** methods of the QueueDiscItem class. The **CoDel** queue disc now makes use of such feature of the base class, hence its Sojourn trace source and the CoDelTimestampTag class
  have been removed.

## Changes to existing API:

- **ParetoRandomVariable** "Mean" attribute has been deprecated,
  the "Scale" Attribute have to be used instead.
  Changing the Mean attribute has no more an effect on the distribution.
  See the documentation for the relationship between Mean, Scale and Shape.
- The default logging timestamp precision has been changed from 6 digits
  to 9 digits, with a fixed format to ensure that 9 digits to the right of
  the decimal point are always printed. Previously, default C++ iostream
  precision and formatting was used.
- Abstract base class **WifiChannel** has been removed. As a result, a Channel type instead of a WifiChannel type
  is now exported by WifiNetDevice.
- The **GetPacketSize** method of **QueueItem** has been renamed **GetSize**
- The **DequeueAll** method of **Queue** has been renamed **Flush**
- The attributes **WifiPhy::TxAntennas** and **WifiPhy::RxAntennas**,
  and the related accessor methods, were replaced by **WifiPhy::MaxSupportedTxSpatialStreams**
  and **WifiPhy::MaxSupportedRxSpatialStreams**. A new attribute **WifiPhy::Antennas**
  was added to allow users to define the number of physical antennas on the device.
- Sockets do not receive anymore broadcast packets, unless they are bound to an "Any" address (0.0.0.0)
  or to a subnet-directed broadcast packet (e.g., x.y.z.0 for a /24 noterok).
  As in Linux, the following rules are now enforced:
  - A socket bound to 0.0.0.0 will receive everything.
  - A socket bound to x.y.z.0/24 will receive subnet-directed broadcast (x.y.z.255) and unicast packets.
  - A socket bound to x.y.z.w will only receive unicast packets.**Previously, a socket bound to an unicast address received also subnet-directed broadcast packets.
  This is not anymore possible**.
- You can now Bind as many socket as you want to an address/port, provided that they are bound to different NetDevices.
  Moreover, BindToNetDevice does not anymore call Bind. In other terms, Bind and BindToNetDevice can be called
  in any order.
  However, it is suggested to use BindToNetDevice *before* Bind in order to avoid conflicts.

## Changes to build system:

- The API scanning process for Python bindings now relies on CastXML, and only 64-bit scans are presently supported (Linux 64-bit systems). Generation of 32-bit scans is documented in the Python chapter of the ns-3 manual.
- Modules can now be located in the 'contrib/' directory in addition to 'src/'
- Behavior for running Python programs was aligned with that of C++ programs; the list of modules built is no longer printed out.

## Changed behavior:

- **MultiModelSpectrumChannel** does not call StartRx for receivers that
  operate on subbands orthogonal to transmitter subbands. Models that depend
  on receiving signals with zero power spectral density from orthogonal bands
  may change their behavior.
  See bug 2467
  for discussion.
- **Packet Tag objects** are no longer constrained to fit within 21
  bytes; a maximum size is no longer enforced.
- The default value of the **TxGain** and **RxGain** attributes in WifiPhy was changed from 1 dB to 0 dB.
- The reported SNR by WifiPhy::MonitorSnifferRx did not include the RxNoiseFigure, but now does; see bug 2783 for discussion.
- **Queue** has been redesigned as a template class object, where the type parameter
  specifies the type of items to be stored in the queue. As a consequence:
  - Being a subclass of Queue, **DropTailQueue** is a template class as well.- Network devices such as SimpleNetDevice, PointToPointNetDevice and CsmaNetDevice
      use a queue of type Queue<Packet> to store the packets to transmit. The SetQueue
      method of their helpers, however, can still be invoked as, e.g.,
      SetQueue ("ns3::DropTailQueue") instead of, e.g., SetQueue
      ("ns3::DropTailQueue<Packet>").
    - The attributes **Mode**, **MaxPackets** and **MaxBytes** are now
      defined by the QueueBase class (which Queue is derived from).
- Queue discs that can operate both in packet mode and byte mode (Red, CoDel, Pie) define their own
  enum QueueDiscMode instead of using QueueBase::QueueMode.
- The CoDel, PIE and RED queue discs require that the size of the internal queue is the same as
  the queue disc limit (previously, it was allowed to be greater than or equal).
- The default value of the **EnableBeaconJitter** attribute in ApWifiMac was changed from false to true.
- The NormalClose() callback of a TcpSocket object used to fire upon leaving TIME\_WAIT state (2\*MSL after FINs have been exchanged). It now fires upon entering TIME\_WAIT state. Timing of the callback for the other path to state CLOSED (through LAST\_ACK) has not been changed.

---

# Changes from ns-3.25 to ns-3.26

## New API:

- A **SocketPriorityTag** is introduced to carry the packet priority. Such a tag
  is added to packets by sockets that support this mechanism (UdpSocketImpl,
  TcpSocketBase and PacketSocket). The base class Socket has a new SetPriority
  method to set the socket priority. When the IPv4 protocol is used, the
  priority is set based on the ToS. See the Socket options section of the
  Network model for more information.
- A **WifiNetDevice::SelectQueue** method has been added to determine the user
  priority of an MSDU. This method is called by the traffic control layer before
  enqueuing a packet in the queue disc, if a queue disc is installed on
  the outgoing device, or passing a packet to the device, otherwise. The
  user priority is set to the three most significant bits of the DS field
  (TOS field in case of IPv4 and Traffic Class field in case of IPv6). The
  packet priority carried by the SocketPriorityTag is set to the user priority.
- The **PfifoFastQueueDisc** classifies packets into bands based on their priority.
  See the pfifo\_fast queue disc section of the Traffic Control Layer model
  for more information.
- A new class **SpectrumWifiPhy** has been introduced that makes use of the
  Spectrum module. Its functionality and API is currently very similar to that
  of the YansWifiPhy, especially because it reuses the same InterferenceHelper
  and ErrorModel classes (for this release). Some example programs in the
  'examples/wireless/' directory, such as 'wifi-spectrum-per-example.cc',
  illustrate how the SpectrumWifiPhy class can be substituted for the default
  YansWifiPhy PHY model.
- We have added support for generating traces for the
  DES Metrics project.
  These can be enabled by adding --enable-des-metrics at configuration;
  you must also use CommandLine in your script. See the API docs
  for class **DesMetrics** for more details.
- The traffic control module now includes the **FQ-CoDel** and **PIE** queue disc
  models, and behavior corresponding to Linux **Byte Queue Limits (BQL)**.
- Several new TCP congestion control variants were introduced, including
  **TCP Vegas, Scalable, Veno, Illinois, Bic, YeAH, and H-TCP**
  congestion control algorithms.

## Changes to existing API:

- **SocketAddressTag** was a long-standing approach to approximate the POSIX
  socket recvfrom behavior (i.e., to know the source address of a packet)
  without actually calling RecvFrom. Experience with this revealed that
  this option was difficult to use with tunnels (the new tag has to
  replace the old one). Moreover, there is no real need
  to create a new API when there is a an existing one (i.e., RecvFrom).
  As a consequence, SocketAddressTag has been completely removed from ns-3.
  Users can use RecvFrom (for UDP), GetPeerName (for TCP), or similar.
- **InetSockAddress** can now store a ToS value, which can be set through its
  SetTos method. The Bind and Connect methods of UDP (UdpSocketImpl) and
  TCP (TcpSocketBase) sockets set the socket ToS value to the value provided
  through the address input parameter (of type InetSockAddress). See the
  Socket options section of the Network model for more information.
- The **QosTag** is removed as it has been superseded by the SocketPriorityTag.
- The **Ipv4L3Protocol::DefaultTos** attribute is removed.
- The attributes **YansWifiPhy::Frequency, YansWifiPhy::ChannelNumber, and
  YansWifiPhy::ChannelWidth**, and the related accessor methods, were moved to
  base class WifiPhy. YansWifiPhy::GetChannelFrequencyMhz() was deleted.
  A new method WifiPhy::DefineChannelNumber () was added to allow users to
  define relationships between channel number, standard, frequency, and channel width.
- The class **WifiSpectrumValueHelper** has been refactored; previously it
  was an abstract base class supporting the WifiSpectrumValue5MhzFactory spectrum
  model. It now contains various static member methods supporting the creation
  of power spectral densities with the granularity of a Wi-Fi OFDM subcarrier
  bandwidth. The class **WifiSpectrumValue5MhzFactory** and its API remain but
  it is not subclassed.
- A new Wifi method **InterferenceHelper::AddForeignSignal** has been introduced to
  support use of the SpectrumWifiPhy (so that non-Wi-Fi signals may be handled
  as noise power).
- A new Wifi attribute **Dcf::TxopLimit** has been introduced to add support for 802.11e TXOP.

## Changes to build system:

- A new waf build option, --check-config, was added to allow users to print the current configuration summary, as appears at the end of ./waf configure. See bug 2459 for discussion.
- The configure summary is now sorted, to make it easier to check the status of optional features.

## Changed behavior:

This section is for behavioral changes to the models that were not due to a bug fix.

- The relationship between Wi-Fi channel number, frequency, channel width,
  and Wi-Fi standard has been revised (see bug 2412). Previously, ChannelNumber
  and Frequency were attributes of class YansWifiPhy, and the frequency was
  defined as the start of the band. Now, Frequency has been redefined to be
  the center frequency of the channel, and the underlying device relies on
  the pair of frequency and channel width to control behavior; the channel
  number and Wi-Fi standard are used as attributes to configure frequency
  and channel width. The wifi module documentation discusses this change
  and the new behavior.
- AODV now honors the TTL in RREQ/RREP and it uses a method
  compliant with RFC 3561. The node search radius is increased progressively. This could increase
  slightly the node search time, but it also decreases the network
  congestion.

---

# Changes from ns-3.24 to ns-3.25

## New API:

- In 'src/internet/test', a new environment is created to test TCP properties.
- The 'src/traffic-control' module has been added, with new API for adding and configuring queue discs and packet filters.
- Related to traffic control, a new interface has been added to the
  NetDevice to provide a queue interface to access device queue state and
  register callbacks used for flow control.
- In 'src/wifi', a new rate control (MinstrelHT) has been added for
  802.11n/ac modes.
- In 'src/wifi', a new helper (WifiMacHelper) is added and is a merged helper from all previously existing MAC helpers (NqosWifiMacHelper, QosWifiMacHelper, HtWifiMacHelper and VhtWifiMacHelper).
- It is now possible to use RIPv2 in IPv4 network simulations.

## Changes to existing API:

- TCP-related changes:
  - Classes TcpRfc793, TcpTahoe, and TcpReno were removed.
  - The 'TcpNewReno' log component was effectively replaced by 'TcpCongestionOps'- TCP Hybla and HighSpeed have been added.
    - Added the concept of Congestion State Machine inside TcpSocketBase.
    - Merged Fast Recovery and Fast Retransmit inside TcpSocketBase.
    - Some member variables have been moved from TcpSocketBase inside TcpSocketState. Attributes are not touched.
    - Congestion control split from TcpSocketBase as subclass of TcpCongestionOps.
    - Added Rx and Tx callbacks on TcpSocketBase.
    - Added BytesInFlight trace source on TcpSocketBase. The trace is updated when the implementation requests the value.
    - Added attributes about the number of connection and data retransmission attempts.
- ns-3 is now capable of serializing SLL (a.k.a. cooked) headers.
  This is used in DCE to allow the generation of pcap directly readable by wireshark.
- In the WifiHelper class in the wifi module, Default has been declared deprecated. This is now immediately handled by the constructor of the class.
- The API for configuring 802.11n/ac aggregation has been modified to be more user friendly. As any MAC layer attributes, aggregation parameters can now also be configured through WifiMacHelper::SetType.
- The class Queue and subclasses derived from it have been changed in two ways:
  - Queues no longer enqueue simple Packets but instead enqueue QueueItem objects, which include Packet but possibly other information such as headers.
  - The attributes governing the mode of operation (packets or bytes) and the maximum size have been moved to base class Queue.
- Users of advanced queues (RED, CoDel) who have been using them directly in the NetDevice will need to adjust to the following changes:
  - RED and CoDel are no longer specializations of the Queue class, but are now specializations of the new QueueDisc class. This means that RED and CoDel can now be installed in the context of the new Traffic Control layer instead of as queues in (some) NetDevices. The reason for such a change is to make the ns-3 stack much more similar to that of real operating systems (Linux has been taken as a reference). Queuing disciplines such as RED and CoDel can now be tested with all the NetDevices, including WifiNetDevices.
  - NetDevices still use queues to buffer packets. The only subclass of Queue currently available for this purpose is DropTailQueue. If one wants to approximate the old behavior, one needs to set the DropTailQueue MaxPackets attribute to very low values, e.g., 1.
  - The Traffic Control layer features a mechanism by which packets dropped by the NetDevice are requeued in the queue disc (more precisely: if NetDevice::Send returns false, the packet is requeued), so that they are retransmitted later. This means that the MAC drop traces may include packets that have not been actually lost, because they have been dropped by the device, requeued by the traffic control layer and successfully retransmitted. To get the correct number of packets that have been actually lost, one has to subtract the number of packets requeued from the number of packets dropped as reported by the MAC drop trace.

## Changes to build system:

- Waf was upgraded to 1.8.19
- A new waf build option, --check-profile, was added to allow users to check the currently active build profile. It is discussed in bug 2202 in the tracker.

## Changed behavior:

This section is for behavioral changes to the models that were not due to a bug fix.

- TCP behavioral changes:
  - TCP closes connection after a number of failed segment retries,
    rather than trying indefinitely. The maximum number of retries, for both SYN
    attempts and data attempts, is controlled by attributes.
  - Congestion algorithms not compliant with Fast Retransmit
    and Fast Recovery (TCP 793, Reno, Tahoe) have been removed.
- 802.11n/ac MPDU aggregation is now enabled by default for both AC\_BE and AC\_VI.
- The introduction of the traffic control layer leads to some additional buffering by default in the stack; when a device queue fills up, additional packets become enqueued at the traffic control layer.

---

# Changes from ns-3.23 to ns-3.24

## New API:

- In 'src/wifi', several changes were made to enable partial 802.11ac support:
  - A new helper (VhtWifiMacHelper) was added to set up a Very high throughput (VHT) MAC entity.
  - A new standard value has been added that enables the new 11ac data rates.
  - A new 11ac preamble has been added.
  - A new information element has been added: VhtCapabilities. This information element is added to the MAC frame header if the node is a VHT node. This VhtCapabilites information element is used to advertise the VHT capabilities of the node to other nodes in the network.
- The ArpCache API was extended to allow the manual removal of ArpCache entries and the addition of permanent (static) entries for IPv4.
- The SimpleChannel in the 'network' module now allows per-NetDevice blacklists, in order to do hidden terminal testcases.

## Changes to existing API:

- The signatures on several TcpHeader methods were changed to take const arguments.
- class TcpL4Protocol replaces Send() methods with SendPacket(), and adds new methods to AddSocket() and RemoveSocket() from a node. Also, a new PacketReceived() method was introduced to get the TCP header of an incoming packet and check its checksum.
- The CongestionWindow and SlowStartThreshold trace sources have been moved from the TCP subclasses such as NewReno, Reno, Tahoe, and Westwood to the TcpSocketBase class.
- The WifiMode object has been refactored:
  - 11n data rates are now renamed according to their MCS value. E.g. OfdmRate65MbpsBW20MHz has been renamed into HtMcs7. 11ac data rates have been defined according to this new renaming.
  - HtWifiMacHelper and VhtWifiMacHelper provide a helper to convert a MCS value into a data rate value.
  - The channel width is no longer tied to the wifimode. It is now included in the TXVECTOR.
  - The physical bitrate is no longer tied to the wifimode. It is computed based on the selected wifimode and on the TXVECTOR parameters (channel width, guard interval and number of spatial streams).

## Changes to build system:

- Waf was upgraded to 1.8.12
- Waf scripts and test.py test runner program were made compatible with Python 3

## Changed behavior:

This section is for behavioral changes to the models that were not due to a bug fix.


---

# Changes from ns-3.22 to ns-3.23

## New API:

- The mobility module includes a GeographicPositions class used to
  convert geographic to cartesian coordinates, and to generate randomly
  distributed geographic coordinates.
- The spectrum module includes new TvSpectrumTransmitter classes and helpers to create television transmitter(s) that transmit PSD spectrums customized by attributes such as modulation type, power, antenna type, channel frequency, etc.

## Changes to existing API:

- In LteSpectrumPhy, LtePhyTxEndCallback and the corresponding methods have been removed, since they were unused.
- In the DataRate class in the network module, CalculateTxTime has been declared deprecated. CalculateBytesTxTime and CalculateBitsTxTime are to be used instead. The return value is a Time, instead of a double.
- In the Wi-Fi InterferenceHelper, the interference event now takes the WifiTxVector as an input parameter, instead of the WifiMode. A similar change was made to the WifiPhy::RxOkCallback signature.

## Changes to build system:

- None

## Changed behavior:

This section is for behavioral changes to the models that were not due to a bug fix.

- In Wi-Fi, HT stations (802.11n) now support two-level aggregation. The InterferenceHelper now distinguishes between the PLCP and regular payload reception, for higher fidelity modeling. ACKs are now sent using legacy rates and preambles. Access points now establish BSSBasicRateSet for control frame transmissions. PLCP header and PLCP payload reception have been decoupled to improve PHY layer modeling accuracy. RTS/CTS with A-MPDU is now fully supported.
- The mesh module was made more compliant to the IEEE 802.11s-2012 standard and packet traces are now parseable by Wireshark.

---

# Changes from ns-3.21 to ns-3.22

## New API:

- New classes were added for the PARF and APARF WiFi power and rate control mechanisms.
- Support for WiFi 802.11n MPDU aggregation has been added.
- Additional support for modeling of vehicular WiFi networks has been added, including the channel-access coordination feature of IEEE 1609.4. In addition, a Basic Safety Message (BSM) packet generator and related statistics-gathering classes have been added to the wave module.
- A complete LTE release bearer procedure is now implemented which can be invoked by calling the new helper method LteHelper::DeActivateDedicatedEpsBearer ().
- It is now possible to print the Neighbor Cache (ARP and NDISC) by using
  the RoutingProtocolHelper
- A TimeProbe class has been added to the data collection framework in
  the stats module, enabling TracedValues emitting values of type
  ns3::Time to be handled by the framework.
- A new attribute 'ClockGranularity' was added to the TcpSocketBase class,
  to control modeling of RTO calculation.

## Changes to existing API:

- Several deprecated classes and class methods were removed, including EmuNetDevice, RandomVariable and derived classes, Packet::PeekData(), Ipv6AddressHelper::NewNetwork(Ipv6Address, Ipv6Prefix), Ipv6InterfaceContainer::SetRouter(), Ipv4Route::GetOutputTtl(), TestCase::AddTestCase(TestCase\*), and TestCase::GetErrorStatus().
- Print methods involving routing tables and neighbor caches, in classes Ipv4RoutingHelper and Ipv6RoutingHelper, were converted to static methods.
- PointerValue attribute types in class UanChannel (NoiseModel), UanPhyGen (PerModel and SinrModel), UanPhyDual (PerModelPhy1, PerModelPhy2, SinrModelPhy1, and SinrModelPhy2), and SimpleNetDevice (TxQueue), were changed from PointerValue type to StringValue type, making them configurable via the Config subsystem.
- WifiPhy::CalculateTxDuration() and WifiPhy::GetPayloadDurationMicroSeconds () now take an additional frequency parameter.
- The attribute 'Recievers' in class YansWifiPhy was misspelled, so
  this has been corrected to 'Receivers'.
- We have now documented the callback function signatures
  for all TracedSources, using an extra (fourth) argument to
  TypeId::AddTraceSource to pass the fully-qualified name
  of the signature typedef. To ensure that future TraceSources
  are similarly documented, the three argument version of
  AddTraceSource has been deprecated.
- The "MinRTO" attribute of the RttEstimator class was moved to the TcpSocketBase class. The "Gain" attribute of the RttMeanDeviation class was replaced
  by new "Alpha" and "Beta" attributes.
- Attributes of the TcpTxBuffer and TcpRxBuffer class are now accessible through the TcpSocketBase class.
- The LrWpanHelper class has a new constructor allowing users to configure a MultiModelSpectrumChannel as an option, and also provides Set/Get API to allow users to access the underlying channel object.

## Changes to build system:

- waf was upgraded to version 1.7.16

## Changed behavior:

This section is for behavioral changes to the models that were not due to a bug fix.

- The default value of the `Speed` attribute of ConstantSpeedPropagationDelayModel was changed from 300,000,000 m/s to 299,792,458 m/s (speed of light in a vacuum), causing propagation delays using this model to vary slightly.
- The LrWpanHelper object was previously instantiating only a LogDistancePropagationLossModel on a SingleModelSpectrumChannel, but no PropagationDelayModel. The constructor now adds by default a ConstantSpeedPropagationDelayModel.
- The Nix-vector routing implementation now uses a lazy flush mechanism,
  which dramatically speeds up the creation of large topologies.

---

# Changes from ns-3.20 to ns-3.21

## New API:

- New "const double& SpectrumValue:: operator[] (size\_t index) const".
- A new TraceSource has been added to TCP sockets: SlowStartThreshold.
- New method CommandLine::AddValue (name, attibutePath) to provide a
  shorthand argument "name" for the Attribute "path". This also has
  the effect of including the help string for the Attribute in the
  Usage message.
- The GSoC 2014 project in the LTE module has brought some additional APIs:
  - a new abstract class LteFfrAlgorithm, which every future
    implementation of frequency reuse algorithm should inherit from
  - a new SAPs: one between MAC Scheduler and FrAlgorithm, one between
    RRC and FrAlgorithm
  - new attribute to enable Uplink Power Control in LteUePhy
  - new LteUePowerControl class, an implementation of Uplink Power Control, which is
    configurable by attributes. ReferenceSignalPower is sent by eNB in SIB2.
    Uplink Power Control in Closed Loop Accumulative Mode is enabled by default
  - seven different Frequency Reuse Algorithms (each has its own attributes):
  - LteFrNoOpAlgorithm
  - LteFrHardAlgorithm
  - LteFrStrictAlgorithm
  - LteFrSoftAlgorithm
  - LteFfrSoftAlgorithm
  - LteFfrEnhancedAlgorithm
  - LteFfrDistributedAlgorithm- attribute in LteFfrAlgorithm to set FrCellTypeId which is used in automatic
    Frequency Reuse algorithm configuration
  - LteHelper has been updated with new methods related to frequency reuse algorithm:
    SetFfrAlgorithmType and SetFfrAlgorithmAttribute
- A new SimpleNetDeviceHelper can now be used to install SimpleNetDevices.
- New PacketSocketServer and PacketSocketClient apps, meant to be used in tests.
- Tcp Timestamps and Window Scale options have been added and are enabled by default (controllable by attribute).
- A new CoDel queue model has been added to the 'internet' module.
- New test macros NS\_TEST\_ASSERT\_MSG\_GT\_OR\_EQ() and NS\_TEST\_EXPECT\_MSG\_GT\_OR\_EQ() have been added.

## Changes to existing API:

- "Icmpv6L4Protocol::ForgeEchoRequest" is now returning a packet with the proper IPv6 header.
- The TCP socket Attribute "SlowStartThreshold" has been renamed "InitialSlowStartThreshold" to
  clarify that the effect is only on the initial value.
- all schedulers were updated to interact with FR entity via FFR-SAP. Only PF, PSS, CQA,
  FD-TBFQ, TD-TBFQ schedulers supports Frequency Reuse functionality. In the beginning
  of scheduling process, schedulers ask FR entity for available RBGs and then ask if UE
  can be scheduled on RB
- eNB RRC interacts with FFR entity via RRC-FFR SAP
- new DL-CQI generation approach was implemented. Now DL-CQI is computed from control channel as signal
  and data channel (if received) as interference. New attribute in LteHelper was added to specify
  DL-CQI generation approach. New approach is default one in LteHelper
- RadioEnvironmentMap can be generated for Data or Control channel and for specified RbId;
  Data or Control channel and RbId can be configured by new attributes in RadioEnvironmentMapHelper
- lte-sinr-chunk-processor refactored to lte-chunk-processor. Removed all lte-xxx-chunk-processor
  implementations
- BindToNetDevice affects also sockets using IPv6.
- BindToNetDevice now calls implicitly Bind (). To bind a socket to a NetDevice and to a specific address,
  the correct sequence is Bind (address) - BindToNetDevice (device). The opposite will raise an error.

## Changes to build system:

- None for this release.

## Changed behavior:

- Behavior will be changed due to the list of bugs fixed (listed in RELEASE\_NOTES); users are requested to review that list as well.

---

# Changes from ns-3.19 to ns-3.20

## New API:

- Models have been added for low-rate, wireless personal area networks
  (LR-WPAN) as specified by IEEE standard 802.15.4 (2006). The current
  emphasis is on the unslotted mode of 802.15.4 operation for use in Zigbee,
  and the scope is limited to enabling a single mode (CSMA/CA) with basic
  data transfer capabilities. Association with PAN coordinators is not yet
  supported, nor the use of extended addressing. Interference is modeled as
  AWGN but this is currently not thoroughly tested. The NetDevice Tx queue
  is not limited, i.e., packets are never dropped due to queue becoming full.
  They may be dropped due to excessive transmission retries or channel access
  failure.
- A new IPv6 routing protocol has been added: RIPng. This protocol is
  an Interior Gateway Protocol and it is available in the Internet module.
- A new LTE MAC downlink scheduling algorithm named Channel and QoS
  Aware (CQA) Scheduler is provided by the new "ns3::CqaFfMacScheduler" object.
- Units may be attached to Time objects, to facilitate specific output
  formats (see Time::As())
- FlowMonitor "SerializeToXml" functions are now directly available
  from the helper.
- Access to OLSR's HNA table has been enabled

## Changes to existing API:

- The SixLowPan model can now use uncompressed IPv6 headers. An option to
  define the minimum compressed packet size has been added.
- MinDistance wsa replaced by MinLoss in FriisPropagationLossModel, to
  better handle conditions outside of the assumed far field region.
- In the DSR model, the attribute DsrOptionRerrHeader::ErrorType" has
  been removed.

## Changes to build system:

- Python 3.3 is now supported for Python bindings for ns-3. Python 3.3
  support for API scanning is not supported. Python 3.2 is not supported.
- Enable selection of high precision int64x64\_t implementation
  at configure time, for debugging purposes.
- Optimized builds are now enabling signed overflow optimization
  (-fstrict-overflow) and for gcc 4.8.2 and greater, also warning for cases
  where an optimizization may occur due to compiler assumption that
  overflow will not occur.

## Changed behavior:

- The Internet FlowMonitor can now track IPv6 packets.
- Ipv6Extension::m\_dropTrace has been removed. Ipv6L3Protocol::m\_dropTrace
  is now fired when appropriate.
- IPv4 identification field value is now dependent on the protocol
  field.
- Point-to-point trace sources now contain PPP headers

---

# Changes from ns-3.18.1 to ns-3.19

## New API:

- A new wifi extension for vehicular simulation support is available in the
  src/wave directory. The current code represents an interim capability to
  realize an IEEE 802.11p-compliant device, but without the WAVE extensions
  (which are planned for a later patch). The WaveNetDevice modelled herein
  enforces that a WAVE-compliant physical layer (at 5.9 GHz) is selected, and
  does not require any association between devices (similar to an adhoc WiFi
  MAC), but is otherwise similar (at this time) to a WifiNetDevice. WAVE
  capabililties of switching between control and service channels, or using
  multiple radios, are not yet modelled.
- New SixLowPanNetDevice class providing a shim between
  IPv6 and real NetDevices. The new module implements 6LoWPAN:
  "Transmission of IPv6 Packets over IEEE 802.15.4 Networks" (see
  RFC 4944 and
  RFC 6262),
  resulting in a heavy header compression for IPv6 packets.
  The module is intended to be used on 802.15.4 NetDevices, but
  it can be used over other NetDevices. See the manual for
  further discussion.
- LteHelper has been updated with some new APIs:
  - new overloaded Attach methods to enable UE to automatically determine
    the eNodeB to attach to (using initial cell selection);
  - new methods related to handover algorithm: SetHandoverAlgorithmType
    and SetHandoverAlgorithmAttribute;
  - a new attribute AnrEnabled to activate/deactivate Automatic Neighbour
    Relation (ANR) function; and
  - a new method SetUeDeviceAttribute for configuring LteUeNetDevice.
- The GSoC 2013 project in the LTE module has brought some additional APIs:
  - a new abstract class LteHandoverAlgorithm, which every future
    implementation of automatic handover trigger should inherit from;
  - new classes LteHandoverAlgorithm and LteAnr as sub-modules of
    LteEnbNetDevice class; both interfacing with the LteEnbRrc sub-module
    through Handover Management SAP and ANR SAP;
  - new attributes in LteEnbNetDevice and LteUeNetDevice classes related
    to Closed Subscriber Group (CSG) functionality in initial cell
    selection;
  - new attributes in LteEnbRrc for configuring UE measurements' filtering
    coefficient (i.e., quantity configuration);
  - a new public method AddUeMeasReportConfig in LteEnbRrc for setting up
    custom UE measurements' reporting configuration; measurement reports
    can then be captured from the RecvMeasurementReport trace source;
    and
  - new trace sources in LteUeRrc to capture more events, such as System
    Information messages (MIB, SIB1, SIB2), initial cell selection, random
    access, and handover.
- A new parallel scheduling algorithm based on null messages, a common
  parallel DES scheduling algorithm, has been added. The null message
  scheduler has better scaling properties when running on some scenarios
  with large numbers of nodes since it does not require a global
  communication.

## Changes to existing API:

- It is now possible to use Ipv6PacketInfoTag from UDP applications in the
  same way as with Ipv4PacketInfoTag. See Doxygen for current limitations in
  using Ipv[4,6]PacketInfoTag to set IP properties.
- A change is introduced for the usage of the EpcHelper
  class. Previously, the EpcHelper class included both the API
  definition and its (only) implementation; as such, users would
  instantiate and use the EpcHelper class directly in their
  simulation programs. From now on,
  EpcHelper is just the base class defining the API, and the
  implementation has been moved to derived classes; as such,
  users are now expected to use one of the derived classes in
  their simulation program. The implementation previously
  provided by the EpcHelper class has been moved to the new
  derived class PointToPointEpcHelper.
- The automatic handover trigger and ANR functions in LTE module have been
  moved from LteEnbRrc class to separate classes. As a result, the related
  attributes, e.g., ServingCellHandoverThreshold, NeighbourCellHandoverOffset,
  EventA2Threshold, and EventA4Threshold have been removed from LteEnbRrc
  class. The equivalent attributes are now in A2A4RsrqHandoverAlgorithm and
  LteAnr classes.
- Master Information Block (MIB) and System Information Block Type 1 (SIB1)
  are now transmitted as LTE control messages, so they are no longer part of
  RRC protocol.
- UE RRC state model in LTE module has been considerably modified and is
  not backward compatible with the previous state model.
- Additional time units (Year, Day, Hour, Minute) were added to the time
  value class that represents simulation time; the largest unit prior to
  this addition was Second.
- SimpleNetDevice and SimpleChannel are not so simple anymore. SimpleNetDevice can be now a
  Broadcast or PointToPoint NetDevice, it can have a limited bandwidth and it uses an output
  queue.

## Changes to build system:

## Changed behavior:

- For the TapBridge device, in UseLocal mode there is a MAC learning function. TapBridge has been waiting for the first packet received from tap interface to set the address of the bridged device to the source address of the first packet. This has caused problems with WiFi. The new behavior is that after connection to the tap interface, ns-3 learns the MAC address of that interface with a system call and immediately sets the address of the bridged device to the learned one. See bug 1777 for more details.
- TapBridge device now correctly implements IsLinkUp() method.
- IPv6 addresses and routing tables are printed like in Linux "route -A inet6" command.
- A change in Ipv[4,6]Interface enforces the correct behaviour of IP
  when a device do not support the minimum MTU requirements.
  This is set to 68 and 1280 octects respectively. IP simulations that
  may have run over devices with smaller MTUs than 68 or 1280, respectively,
  will no longer be able to use such devices.

---

# Changes from ns-3.18 to ns-3.18.1

## New API:

- It is now possible to randomize the time of the first beacon from an
  access point. Use an attribute "EnableBeaconJitter" to enable/disable
  this feature.
- A new FixedRoomPositionAllocator helper class is available; it
  allows one to generate a random position uniformly distributed in the
  volume of a chosen room inside a chosen building.

## Changes to existing API:

- Logging wildcards: allow "\*\*\*" as synonym for "\*=\*\*" to turn on all logging.
- The log component list ("NS\_LOG=print-list") is now printed alphabetically.
- Some deprecated IEEE 802.11p code has been removed from the wifi module

## Changes to build system:

- The Python API scanning system (./waf --apiscan) has been fixed (bug 1622)
- Waf has been upgraded from 1.7.11 to 1.7.13

## Changed behavior:

- Wifi simulations have additional jitter on AP beaconing (see above) and some bug fixes have been applied to wifi module (see RELEASE\_NOTES)

---

# Changes from ns-3.17 to ns-3.18

## New API:

- New features have been added to the LTE module:
  - PHY support for UE measurements (RSRP and RSRQ)
  - RRC support for UE measurements (configuration, execution, reporting)
  - Automatic Handover trigger based on RRC UE measurement reports- Data collection components have been added in the 'src/stats' module.
    Data collection includes a Probe class that attaches to ns-3 trace
    sources to filter their output, and two Aggregator classes for
    marshaling probed data into text files or gnuplot plots. The ns-3
    tutorial has been extended to illustrate basic functionality.
  - In 'src/wifi', several changes were made to enable partial 802.11n support:
    - A new helper (HtWifiMacHelper) was added to set up a High Throughput (HT) MAC entity
    - New attributes were added to help the user setup a High Throughput (HT) PHY entity. These attributes can be set using the YansWifiPhyHelper
    - A new standard value has been added that enables the new 11n data rates.
    - New 11n preambles has been added (Mixed format and greenfield). To be able to change Tx duration according to the preamble used, a new class TxVector has been added to carry the transmission parameters (mode, preamble, stbc,..). Several functions have been updated to allow the passage of TxVector instead of WifiMode in MacLow, WifiRemoteStationManager, WifiPhy, YansWifiPhy,..
    - A new information element has been added: HTCapabilities. This information element is added to the MAC frame header if the node is an HT node. This HTCapabilites information element is used to advertise the HT capabilities of the node to other nodes in the network- InternetStackHelper has two new functions:SetIpv4ArpJitter (bool enable)
      and SetIpv6NsRsJitter (bool enable) to enable/disable
      the random jitter on the tranmission of IPv4 ARP Request and IPv6 NS/RS.
    - Bounds on valid time inputs for time attributes can now be enabled.
      See attribute-test-suite.cc for an example.
    - New generic hash function interface provided in the simulation core.
      Two hash functions are provided: murmur3 (default), and the venerable
      FNV1a. See the Hash Functions section in the ns-3 manual.
    - New Mac16Address has been added. It can be used with IPv6 to make
      an Autoconfigured address.
    - Mac64Address support has been extended. It can now be used with
      IPv6 to make an Autoconfigured address.
    - IPv6 can now detect and use Path-MTU. See
      examples/ipv6/fragmentation-ipv6-two-MTU.cc for an example.
    - Radvd application has a new Helper. See the updated
      examples/ipv6/radvd.cc for an example.

## Changes to existing API:

- The Ipv6InterfaceContainer functions to set a node in forwarding state (i.e., a router)
  and to install a default router in a group of nodes have been extensively changed.
  The old function void Ipv6InterfaceContainer::SetRouter (uint32\_t i, bool router)
  is now DEPRECATED.
- The documentation's IPv6 addresses (2001:db8::/32, RFC 3849) are now
  dropped by routers.
- The 'src/tools' module has been removed, and most files migrated to
  'src/stats'. For users of these programs (the statistics-processing
  in average.h, or the gnuplot support), the main change is likely to be
  replacing the inclusion of "tools-module.h" with "stats-module.h".
  Users of the event garbage collector, previously in tools, will now
  include it from the core module.
- The Ipv6 UnicastForwardCallback and MulticastForwardCallback
  have a new parameter, the NetDevice the packet has been received from.
  Existing Ipv6RoutingProtocols should update their RouteInput function
  accordingly, e.g., from ucb (rtentry, p, header); to ucb (idev, rtentry, p, header);
- The previous buildings module relied on a specific MobilityModel called
  BuildingsMobilityModel, which supported buildings but only allowed
  static positions. This mobility model has been removed. Now, the
  Buildings module instead relies on a new class called
  MobilityBuildingInfo which can be aggregated to any MobilityModel. This
  allows having moving nodes in presence of buildings with any of
  the existing MobilityModels.
- All functions in WifiRemoteStationManager named GetXxxMode have been changed to GetXxxTxVector

## Changes to build system:

- Make references to bug id's in doxygen comments with
  \bugid{num}, where num is the bug id number. This
  form will generate a link to the bug in the bug database.

## Changed behavior:

- Now it is possible to request printing command line arguments to the
  desired output stream using PrintHelp or operator <<

  ```
    CommandLine cmd;
    cmd.Parse (argc, argv);
  ...

    std::cerr << cmd;
  ```

  or

  ```
    cmd.PrintHelp (std::cerr);
  ```
- Command line boolean arguments specified with no integer value (e.g. "--boolArg") will toggle the value from the default, instead of always setting the value to true.
- IPv4's ARP Request and IPv6's NS/RS are now transmitted with a random delay.
  The delay is, by default, a uniform random variable in time between 0 and 10ms.
  This is aimed at preventing reception errors due to collisions during wifi broadcasts when the sending behavior is synchronized (e.g. due to applications starting at the same time on several different nodes).
  This behaviour can be modified by using ArpL3Protocol's
  RequestJitter and Icmpv6L4Protocol's SolicitationJitter
  attributes or by using the new InternetStackHelper functions.
- AODV Hellos are disabled by default. The performance with Hellos enabled and disabled are almost identical. With Hellos enabled, AODV will suppress hellos from transmission, if any recent broadcast such as RREQ was transmitted. The attribute ns3::aodv::RoutingProtocol::EnableHello can be used to enable/disable Hellos.

---

# Changes from ns-3.16 to ns-3.17

## New API:

- New TCP Westwood and Westwood+ models- New FdNetDevice class providing a special NetDevice that is able to read
    and write traffic from a file descriptor. Three helpers are provided
    to associate the file descriptor with different underlying devices:
    - EmuFdNetDeviceHelper (to associate the |ns3| device with a physical
      device in the host machine). This helper is intended to
      eventually replace the EmuNetDevice in src/emu.
    - TapFdNetDeviceHelper (to associate the ns-3 device with the file
      descriptor from a tap device in the host machine)
    - PlanteLabFdNetDeviceHelper (to automate the creation of tap devices
      in PlanetLab nodes, enabling |ns3| simulations that can send and
      receive traffic though the Internet using PlanetLab resource.
  - In Ipv4ClickRouting, the following APIs were added:
    - Ipv4ClickRouting::SetDefines(), accessible through ClickInternetStackHelper::SetDefines(), for the user to set Click defines from the ns-3 simulation file.
    - SIMCLICK\_GET\_RANDOM\_INT click-to-simulator command for ns-3 to drive Click's random number generation.
  - LTE module
    - New user-visible LTE API
      - Two new methods have been added to LteHelper to enable the X2-based handover functionality: AddX2Interface, which setups the X2 interface between two eNBs, and HandoverRequest, which is a convenience method that schedules an explicit handover event to be executed at a given point in the simulation.
      - the new LteHelper method EnablePhyTraces can now be used to enable the new PHY traces
    - New internal LTE API
      - New LTE control message classes DlHarqFeedbackLteControlMessage,
        RachPreambleLteControlMessage, RarLteControlMessage, MibLteControlMessage
      - New class UeManager- New LteRadioBearerInfo subclasses LteSignalingRadioBearerInfo,
          LteDataRadioBearerInfo
        - New LteSinrChunkProcessor subclasses LteRsReceivedPowerChunkProcessor,
          LteInterferencePowerChunkProcessor
  - New DSR API
    - Added PassiveBuffer class to save maintenance packet entry for passive acknowledgment option
    - Added FindSourceEntry function in RreqTable class to keep track of route request entry received from same source node
    - Added NotifyDataReciept function in DsrRouting class to notify the data receipt of the next hop from link layer. This is used for the link layer acknowledgment.
  - New Tag, PacketSocketTag, to carry the destination address of a packet and the packet type
  - New Tag, DeviceNameTag, to carry the ns3 device name from where a packet is coming
  - New Error Model, BurstError model, to determine which bursts of packets are errored corresponding to an underlying distribution, burst rate, and burst size

## Changes to existing API:

- ns3::Object and subclasses DoStart has been renamed to DoInitialize
- ns3::Object and subclasses Start has been renamed to Initialize
- EnergySource StartDeviceModels renamed to InitializeDeviceModels
- A typo was fixed in an LTE variable name. The variable ns3::AllocationRetentionPriority::preemprionVulnerability was changed to preemptionVulnerability.
- Changes in TestCase API
  - TestCase has new enumeration TestDuration containing QUICK, EXTENSIVE, TAKES\_FOREVER
  - TestCase constructor now requires TestDuration, old constructor marked deprecated
- Changes in LTE API
  - User-visible LTE API
    - The previous LteHelper method ActivateEpsBearer has been now replaced by two alternative methods: ActivateDataRadioBearer (to be used when the EPC model is not used) and ActivateDedicatedEpsBearer (to be used when the EPC model is used). In the case where the EPC model is used, the default EPS bearer is not automatically activated without the need for a specific method to be called.
  - Internal LTE API
    - EpcHelper added methods AddUe, AddX2Interface. Method AddEnb now requires a cellId. Signature of ActivateEpsBearer changed to void ActivateEpsBearer (Ptr ueLteDevice, uint64\_t imsi, Ptr tft, EpsBearer bearer)
    - LteHelper added methods EnableDlPhyTraces, EnableUlPhyTraces, EnableDlTxPhyTraces, EnableUlTxPhyTraces, EnableDlRxPhyTraces, EnableUlRxPhyTraces
    - LteHelper removed methods EnableDlRlcTraces, EnableUlRlcTraces, EnableDlPdcpTraces, EnableUlPdcpTraces
    - RadioBearerStatsCalculator added methods (Set/Get)StartTime, (Set/Get)Epoch, RescheduleEndEpoch, EndEpoch
    - RadioBearerStatsCalculator removed methods StartEpoch, CheckEpoch
    - RadioBearerStatsCalculator methods UlTxPdu, DlRxPdu now require a cellId
    - EpcEnbApplication constructor now requires Ipv4Addresses enbS1uAddress and sgwS1uAddress as well as cellId
    - EpcEnbApplication added methods SetS1SapUser, GetS1SapProvider, SetS1apSapMme and GetS1apSapEnb
    - EpcEnbApplication removed method ErabSetupRequest
    - EpcSgwPgwApplication added methods SetS11SapMme, GetS11SapSgw, AddEnb, AddUe, SetUeAddress
    - lte-common.h new structs PhyTransmissionStatParameters and PhyReceptionStatParameters used in TracedCallbacks
    - LteControlMessage new message types DL\_HARQ, RACH\_PREAMBLE, RAR, MIB
    - LteEnbCmacSapProvider new methods RemoveUe, GetRachConfig, AllocateNcRaPreamble, AllocateTemporaryCellRnti
    - LteEnbPhy new methods GetLteEnbCphySapProvider, SetLteEnbCphySapUser, GetDlSpectrumPhy, GetUlSpectrumPhy, CreateSrsReport
    - LteEnbPhy methods DoSendMacPdu, DoSetTransmissionMode, DoSetSrsConfigurationIndex, DoGetMacChTtiDelay, DoSendLteControlMessage, AddUePhy, DeleteUePhy made private
    - LteEnbPhySapProvider removed methods SetBandwidth, SetTransmissionMode, SetSrsConfigurationIndex, SetCellId
    - LteEnbPhySapUser added methods ReceiveRachPreamble, UlInfoListElementHarqFeeback, DlInfoListElementHarqFeeback
    - LtePdcp added methods (Set/Get)Status
    - LtePdcp DoTransmitRrcPdu renamed DoTransmitPdcpSdu
    - LteUeRrc new enum State. New methods SetLteUeCphySapProvider, GetLteUeCphySapUser, SetLteUeRrcSapUser, GetLteUeRrcSapProvider, GetState, GetDlEarfcn, GetDlBandwidth, GetUlBandwidth, GetCellId, SetUseRlcSm . GetRnti made const.
    - LteUeRrc removed methods ReleaseRadioBearer, GetLcIdVector, SetForwardUpCallback, DoRrcConfigurationUpdateInd
    - LtePdcpSapProvider struct TransmitRrcPduParameters renamed TransmitPdcpSduParameters. Method TransmitRrcPdu renamed TransmitPdcpSdu
    - LtePdcpSapUser struct ReceiveRrcPduParameters renamed ReceivePdcpSduParameters. Method ReceiveRrcPdu renamed TransmitPdcpSdu
    - LtePdcpSpecificLtePdcpSapProvider method TransmitRrcPdu renamed TransmitPdcpSdu
    - LtePdcpSpecificLtePdcpSapUser method ReceiveRrcPdu renamed ReceivePdcpSdu. Method ReceiveRrcPdu renamed ReceivePdcpSdu
    - LtePhy removed methods DoSetBandwidth and DoSetEarfcn
    - LtePhy added methods ReportInterference and ReportRsReceivedPower
    - LteSpectrumPhy added methods SetHarqPhyModule, Reset, SetLtePhyDlHarqFeedbackCallback, SetLtePhyUlHarqFeedbackCallback, AddRsPowerChunkProcessor, AddInterferenceChunkProcessor
    - LteUeCphySapProvider removed methods ConfigureRach, StartContentionBasedRandomAccessProcedure, StartNonContentionBasedRandomAccessProcedure
    - LteUeMac added method AssignStreams
    - LteUeNetDevice methods GetMac, GetRrc, GetImsi made const
    - LteUeNetDevice new method GetNas
    - LteUePhy new methods GetLteUeCphySapProvider, SetLteUeCphySapUser, GetDlSpectrumPhy, GetUlSpectrumPhy, ReportInterference, ReportRsReceivedPower, ReceiveLteDlHarqFeedback
    - LteUePhy DoSendMacPdu, DoSendLteControlMessage, DoSetTransmissionMode, DoSetSrsConfigurationIndex made private
    - LteUePhySapProvider removed methods SetBandwidth, SetTransmissionMode, SetSrsConfigurationIndex
    - LteUePhySapProvider added method SendRachPreamble- AnimationInterface method EnableIpv4RouteTracking returns reference to calling AnimationInterface object
  - To make the API more uniform across the various
    PropagationLossModel classes, the Set/GetLambda methods of the
    FriisPropagationLossModel and TwoRayGroundPropagationLossModel
    classes have been changed to Set/GetFrequency, and now a Frequency
    attribute is exported which replaces the pre-existing Lambda
    attribute. Any previous user code setting a value for Lambda should
    be changed to set instead a value of Frequency = C / Lambda, with C
    = 299792458.0.

## Changes to build system:

- Waf shipped with ns-3 has been upgraded to version 1.7.10 and custom
  pkg-config generator has been replaced by Waf's builtin tool.

## Changed behavior:

- DSR link layer notification has changed. The model originally used
  "TxErrHeader" in Ptr to indicate the transmission
  error of a specific packet in link layer; however, it was not working
  correctly. The model now uses a different path to implement
  the link layer notification mechanism; specifically, looking into the
  trace file to find packet receive events. If the model finds one
  receive event for the data packet, it is used as the indicator for
  successful data delivery.

---

# Changes from ns-3.15 to ns-3.16

## New API:

- In the Socket class, the following functions were added:
  - (Set/Get)IpTos - sets IP Type of Service field in the IP headers.
  - (Set/Is)IpRecvTos - tells the socket to pass information about IP ToS up the stack (by adding SocketIpTosTag to the packet).
  - (Set/Get)IpTtl - sets IP Time to live field in the IP headers.
  - (Set/Is)RecvIpTtl - tells the socket to pass information about IP TTL up the stack (by adding SocketIpTtlTag to the packet).
  - (Set/Is)Ipv6Tclass - sets Traffic Class field in the IPv6 headers.
  - (Set/Is)Ipv6RecvTclass - tells the socket to pass information about IPv6 TCLASS up the stack (by adding SocketIpv6TclassTag to the packet).
  - (Set/Get)Ipv6HopLimit - sets Hop Limit field in the IPv6 headers.
  - (Set/Is)Ipv6RecvHopLimit - tells the socket to pass information about IPv6 HOPLIMIT up the stack (by adding SocketIpv6HoplimitTag to the packet).A user can call these functions to set/get the corresponding socket option. See examples/socket/socket-options-ipv4.cc and examples/socket/socket-options-ipv6.cc for examples.

## Changes to existing API:

- In the MobilityHelper class, the functions EnableAscii () and EnableAsciiAll () were changed to use output stream wrappers rather than standard C++ ostreams. The purpose of this change was to make them behave analogously to other helpers in ns-3 that generate ascii traces. Now, the file stream that is open in MobilityHelper is closed nicely upon asserts and program exits.

## Changes to build system:

- It's now possible to use distcc when building ns-3. See tutorial for details.

## Changed behavior:

- Sending a packet through Ipv4RawSocket now supports checksum in the Ipv4Header. It is still not possible to manually put in arbitrary checksum as the checksum is automatically calculated at Ipv4L3Protocol. The user has to enable checksum globally for this to work. Simply calling Ipv4Header::EnableChecksum() for a single Ipv4Header will not work.
- Now MultiModelSpectrumChannel allows a SpectrumPhy instance to change SpectrumModel at runtime by issuing a call to MultiModelSpectrumChannel::AddRx (). Previously, MultiModelSpectrumChannel required each SpectrumPhy instance to stick with the same SpectrumModel for the whole simulation.

---

# Changes from ns-3.14 to ns-3.15

## New API:

- A RandomVariableStreamHelper has been introduced to assist with
  using the Config subsystem path names to assign fixed stream numbers
  to RandomVariableStream objects.

## Changes to existing API:

- Derived classes of RandomVariable (i.e. the random variable
  implementations) have been ported to a new RandomVariableStream base class.- For a given distribution DistributionVariable (such as UniformVariable),
    the new class name is DistributionRandomVariable (such as
    UniformRandomVariable).
  - The new implementations are also derived from class ns3::Object and
    are handled using the ns-3 smart pointer (Ptr) class.
  - The new variable classes also have a new attributed called "Stream"
    which allows them to be assigned to a fix stream index when assigned
    to the underlying pseudo-random stream of numbers.

## Changes to build system:


## Changed behavior:

- Programs using random variables or models that include random variables
  may exhibit changed output for a given run number or seed, due to a possible
  change in the order in which random variables are assigned to underlying
  pseudo-random sequences. Consult the manual for more information regarding
  this.

---

# Changes from ns-3.13 to ns-3.14

## New API:

- The new class AntennaModel provides an API for modeling the radiation pattern of antennas.
- The new buildings module introduces an API (classes, helpers, etc)
  to model the presence of buildings in a wireless network topology.
- The LENA project's implementation of the LTE Mac Scheduler Interface Specification
  standardized by the Small Cell Forum (formerly Femto Forum) is now available for
  use with the LTE module.

## Changes to existing API:

- The Ipv6RawSocketImpl "IcmpFilter" attribute has been removed. Six
  new member functions have been added to enable the same functionality.
- IPv6 support for TCP and UDP has been implemented. Socket functions
  that take an address [e.g. Send (), Connect (), Bind ()] can accept an
  ns3::Ipv6Address or a ns3::Address in addition to taking an ns3::Ipv4Address.
  (Note that the ns3::Address must contain a ns3::Ipv6Address or a ns3::Ipv4Address,
  otherwise these functions will return an error).
  Internally, the socket now stores the remote address as a type "ns3::Address"
  instead of a type "ns3::Ipv4Address". The IPv6 Routing Header extension is not
  currently supported in ns3 and will not be reflected in the TCP and UDP checksum
  calculations per RFC 2460. Also note that UDP checksums for IPv6 packets are
  required per RFC, but remain optional and disabled by default in ns3 (in the
  interest of performance).
- When calling Bind () on a socket without an address, the behavior remains the
  same: it will bind to the IPv4 "any" address (0.0.0.0). In order to Bind () to
  the IPv6 "any" address in a similar fashion, use "Bind6 ()".
- The prototype for the RxCallback function in the Ipv6EndPoint was changed.
  It now includes the destination IPv6 address of the end point which was
  needed for TCP. This lead to a small change in the UDP and ICMPv6 L4
  protocols as well.
- Ipv6RoutingHelper can now print the IPv6 Routing Tables at specific
  intervals or time. Exactly like Ipv4RoutingHelper do.
- New "SendIcmpv6Redirect" attribute (and getter/setter functions) to
  Ipv6L3Protocol. The behavior is similar to Linux's conf "send\_redirects",
  i.e., enable/disable the ICMPv6 Redirect sending.
- The SpectrumPhy abstract class now has a new method

  ```
  virtual Ptr<AntennaModel> GetRxAntenna () = 0;
  ```

  that all derived classes need to implement in order to integrate properly with the newly added antenna model. In addition, a new member variable "Ptr<AntennaModel> txAntenna" has been added to SpectrumSignalParameters in order to allow derived SpectrumPhy classes to provide information about the antenna model used for the transmission of a waveform.
- The Ns2CalendarScheduler event scheduler has been removed.
- ErrorUnit enum has been moved into RateErrorModel class, and symbols EU\_BIT, EU\_BYTE and EU\_PKT have been renamed to RateErrorModel::ERROR\_UNIT\_BIT, RateErrorModel::ERROR\_UNIT\_BYTE and RateErrorModel::ERROR\_UNIT\_PACKET. RateErrorModel class attribute "ErrorUnit" values have also been renamed for consistency, and are now "ERROR\_UNIT\_BIT", "ERROR\_UNIT\_BYTE", "ERROR\_UNIT\_PACKET".
- QueueMode enum from DropTailQueue and RedQueue classes has been unified and moved to Queueu class. Symbols DropTailQueue::PACKETS and DropTailQueue::BYTES are now named Queue::QUEUE\_MODE\_PACKETS and DropTailQueue::QUEUE\_MODE\_BYTES. In addition, DropTailQueue and RedQueue class attributes "Mode" have been renamed for consistency from "Packets" and "Bytes" to "QUEUE\_MODE\_PACKETS" and "QUEUE\_MODE\_BYTES".
- The API of the LTE module has undergone a significant redesign with
  the merge of the code from the LENA project. The new API is not
  backwards compatible with the previous version of the LTE module.
- The Ipv6AddressHelper API has been aligned with the Ipv4AddressHelper API.
  The helper can be set with a call to Ipv6AddressHelper::SetBase
  (Ipv6Address network, Ipv6Prefix prefix) instead of NewNetwork
  (Ipv6Address network, Ipv6Prefix prefix). A new NewAddress (void) method
  has been added. Typical usage will involve calls to SetBase (), NewNetwork (),
  and NewAddress (), as in class Ipv4AddressHelper.

## Changes to build system:

- The following files are removed:

  ```
    src/internet/model/ipv4-l4-protocol.cc
    src/internet/model/ipv4-l4-protocol.h
    src/internet/model/ipv6-l4-protocol.cc
    src/internet/model/ipv6-l4-protocol.h
  ```

  and replaced with:

  ```
    src/internet/model/ip-l4-protocol.cc
    src/internet/model/ip-l4-protocol.h
  ```

## Changed behavior:

- Dual-stacked IPv6 sockets are implemented. An IPv6 socket can accept
  an IPv4 connection, returning the senders address as an IPv4-mapped address
  (IPV6\_V6ONLY socket option is not implemented).
- The following examples/application/helpers were modified to support IPv6:

  ```
  csma-layout/examples/csma-star [*]
  netanim/examples/star-animation [*]
  point-to-point-layout/model/point-to-point-star.cc
  point-to-point-layout/model/point-to-point-grid.cc
  point-to-point-layout/model/point-to-point-dumbbell.cc
  examples/udp/udp-echo [*]
  examples/udp-client-server/udp-client-server [*]
  examples/udp-client-server/udp-trace-client-server [*]
  applications/helper/udp-echo-helper
  applications/model/udp-client
  applications/model/udp-echo-client
  applications/model/udp-echo-server
  applications/model/udp-server
  applications/model/udp-trace-client

  [*]  Added '--useIpv6' flag to switch between IPv4 and IPv6
  ```

---

# Changes from ns-3.12 to ns-3.13

## Changes to build system:

- The underlying version of waf used by ns-3 was upgraded to 1.6.7.
  This has a few changes for users and developers:
  - by default, "build" no longer has a subdirectory debug or optimized.
    To get different build directories for different build types, you can use
    the waf configure -o  option, e.g.:

    ```
      ./waf configure -o shared
      ./waf configure --enable-static -o static
    ```
  - (for developers) the ns3headers taskgen needs to be created with a
    features parameter name:

    ```
      -  headers = bld.new_task_gen('ns3header')
      +  headers = bld.new_task_gen(features=['ns3header'])
    ```

    - no longer need to edit src/wscript to add a module, just create your
      module directory inside src and ns-3 will pick it up- In WAF 1.6, adding -Dxxx options is done via the DEFINES env. var.
        instead of CXXDEFINES- waf env values are always lists now, e.g. env['PYTHON'] returns
          ['/usr/bin/python'], so you may need to add [0] to the value in some places

## New API:

- In the mobility module, there is a new MobilityModel::GetRelativeSpeed() method returning the relative speed of two objects.
- A new Ipv6AddressGenerator class was added to generate sequential
  addresses from a provided base prefix and interfaceId. It also will detect
  duplicate address assignments.

## Changes to existing API:

- In the spectrum module, the parameters to SpectrumChannel::StartTx () and SpectrumPhy::StartRx () methods are now passed using the new struct SpectrumSignalParameters. This new struct supports inheritance, hence it allows technology-specific PHY implementations to provide technology-specific parameters in SpectrumChannel::StartTx() and SpectrumPhy::StartRx(), while at the same time keeping a set of technology-independent parameters common across all spectrum-enabled PHY implementations (i.e., the duration and the power spectral density which are needed for interference calculation). Additionally, the SpectrumType class has been removed, since now the type of a spectrum signal can be inferred by doing a dynamic cast on SpectrumSignalParameters. See the Spectrum API change discussion on ns-developers for the motivation behind this API change.
- The WifiPhyStandard enumerators for specifying half- and quarter-channel
  width standards has had a change in capitalization:
  - WIFI\_PHY\_STANDARD\_80211\_10Mhz was changed to WIFI\_PHY\_STANDARD\_80211\_10MHZ- WIFI\_PHY\_STANDARD\_80211\_5Mhz was changed to WIFI\_PHY\_STANDARD\_80211\_5MHZ- In the SpectrumPhy base class, the methods to get/set the
    MobilityModel and the NetDevice were previously working with
    opaque Ptr<Object>. Now all these methods have been
    changed so that they work with Ptr<NetDevice>
    and Ptr<MobilityModel> as appropriate. See Bug 1271 on
    bugzilla for the motivation.

## Changed behavior:

- TCP bug fixes
  - Connection retries count is a separate variable with the retries limit, so cloned sockets can reset the count- Fix bug on RTO that may halt the data flow- Make TCP endpoints always holds the accurate address:port info- RST packet is sent on closed sockets- Fix congestion window sizing problem upon partial ACK in TcpNewReno- Acknowledgement is sent, rather than staying silent, upon arrival of unacceptable packets- Advance TcpSocketBase::m\_nextTxSequence after RTO- TCP enhancements
    - Latest RTT value now stored in variable TcpSocketBase::m\_lastRtt- The list variable TcpL4Protocol::m\_sockets now always holds all the created, running TcpSocketBase objects- Maximum announced window size now an attribute, ns3::TcpSocketBase::MaxWindowSize- TcpHeader now recognizes ECE and CWR flags (c.f. RFC3168)- Added TCP option handling call in TcpSocketBase for future extension- Data out of range (i.e. outsize acceptable range of receive window) now computed on bytes, not packets- TCP moves from time-wait state to closed state after twice the time specified by attribute ns3:TcpSocketBase::MaxSegLifeTime- TcpNewReno supports limited transmit (RFC3042) if asserting boolean attribute ns3::TcpNewReno::LimitedTransmit- Nagle's algorithm supported. Default off, turn on by calling TcpSocket::SetTcpNoDelay(true)

---

# Changes from ns-3.11 to ns-3.12

## Changes to build system:


## New API:

- New method, RegularWifiMac::SetPromisc (void), to set the interface
  to promiscuous mode.

## Changes to existing API:

- The spelling of the attribute 'IntialCellVoltage' from LiIonEnergySource
  was corrected to 'InitialCellVoltage'; this will affect existing users who
  were using the attribute with the misspelling.- Two trace sources in class WifiPhy have had their names changed:
    - 'PromiscSnifferRx' is now 'MonitorSnifferRx'- 'PromiscSnifferTx' is now 'MonitorSnifferTx'

## Changed behavior:

- IPv4 fragmentation is now supported.

---

# Changes from ns-3.10 to ns-3.11

## Changes to build system:

- **Examples and tests are no longer built by default in ns-3**

  You can now make examples and tests be built in ns-3 in two ways.
  1. Using build.py when ns-3 is built for the first time:

     ```
         ./build.py --enable-examples --enable-tests
     ```

     - Using waf once ns-3 has been built:

       ```
           ./waf configure --enable-examples --enable-tests
       ```
- **Subsets of modules can be enabled using the ns-3 configuration file**

  A new configuration file, .ns3rc, has been added to ns-3 that
  specifies the modules that should be enabled during the ns-3 build.
  See the documentation for details.

## New API:

- **int64x64\_t**

  The **int64x64\_t** type implements all the C++ arithmetic operators to behave like one of the
  C++ native types. It is a 64.64 integer type which means that it is a 128bit integer type with
  64 bits of fractional precision. The existing **Time** type is now automatically convertible to
  **int64x64\_t** to allow arbitrarily complex arithmetic operations on the content of **Time**
  objects. The implementation of **int64x64\_t** is based on the previously-existing
  **HighPrecision** type and supersedes it.

## Changes to existing API:

- **Wifi TX duration calculation moved from InterferenceHelper to WifiPhy**

  The following static methods have been moved from the InterferenceHelper class to the WifiPhy class:

  ```
  static Time CalculateTxDuration (uint32_t size, WifiMode payloadMode, enum WifiPreamble preamble);
  static WifiMode GetPlcpHeaderMode (WifiMode payloadMode, WifiPreamble preamble);
  static uint32_t GetPlcpHeaderDurationMicroSeconds (WifiMode payloadMode, WifiPreamble preamble);
  static uint32_t GetPlcpPreambleDurationMicroSeconds (WifiMode payloadMode, WifiPreamble preamble);
  static uint32_t GetPayloadDurationMicroSeconds (uint32_t size, WifiMode payloadMode);
  ```
- **Test cases no longer return a boolean value**

  Unit test case DoRun() functions no longer return a bool value. Now, they don't return a value at all. The motivation for this change was to disallow users from merely returning "true" from a test case to force an error to be recorded. Instead, test case macros should be used.
- **PhyMac renamed to GenericPhy**

  The PhyMac interface previously defined in phy-mac.h has been
  renamed to GenericPhy interface and moved to a new file
  generic-phy.h. The related variables and methods have been renamed accordingly.
- **Scalar**

  The Scalar type has been removed. Typical code such as:

  ```
  Time tmp = ...;
  Time result = tmp * Scalar (5);
  ```

  Can now be rewritten as:

  ```
  Time tmp = ...;
  Time result = Time (tmp * 5);
  ```
- **Multicast GetOutputTtl() commands**

  As part of bug 1047 rework to enable multicast routes on nodes with
  more than 16 interfaces, the methods Ipv4MulticastRoute::GetOutputTtl ()
  and Ipv6MulticastRoute::GetOutputTtl () have been modified to return
  a std::map of interface IDs and TTLs for the route.

## Changed behavior:

- If the data inside the TCP buffer is less than the available window, TCP tries to ask for more data to the application, in the hope of filling the usable transmission window. In some cases, this change allows sending bigger packets than the previous versions, optimizing the transmission.
- In TCP, the ACK is now processed before invoking any routine that deals with the segment sending, except in case of retransmissions.

---

# Changes from ns-3.9 to ns-3.10

## Changes to build system:

- **Regression tests are no longer run using waf**

  All regression testing is now being done in test.py. As a result, a
  separate reference trace repository is no longer needed to perform
  regression tests. Tests that require comparison against known good traces
  can still be run from test.py. The --regression option for waf has been
  removed. However, the "-r" option to download.py has been kept to
  allow users to fetch older revisions of ns-3 that contain these traces.

  - **Documentation converted to Sphinx**

    Project documentation (manual, tutorial, and testing) have been
    converted to Sphinx from the GNU Texinfo markup format.

## New API:

- **Pyviz visualizer**

  A Python-based visualizer called pyviz is now integrated with ns-3.
  For Python simulations, there is an API to start the visualizer. You
  have to import the visualizer module, and call visualizer.start()
  instead of ns3.Simulator.Run(). For C++ simulations, there is no API.
  For C++ simulations (but also works for Python ones) you need to set
  the GlobalValue SimulatorImplementationType to
  "ns3::VisualSimulatorImpl". This can be set from the command-line,
  for example (add the
  --SimulatorImplementationType=ns3::VisualSimulatorImpl
  option), or via the waf option --visualizer, in addition to
  the usual --run option to run programs.
- **WaypointMobility attributes**

  Two attributes were added to WaypointMobility model: LazyNotify and
  InitialPositionIs Waypoint. See RELEASE\_NOTES for details.
- **802.11g rates for ERP-OFDM added**

  New WifiModes of the form ErpOfdmRatexxMbps, where xx is the rate
  in Mbps (6, 9, 12, 18, 24, 36, 48, 54), are available for 802.11g.
  More details are in the RELEASE\_NOTES.
- **Socket::GetSocketType ()**

  This is analogous to getsockopt(SO\_TYPE). ipv4-raw-socket, ipv6-raw-socket,
  and packet-socket return NS3\_SOCK\_RAW. tcp-socket and nsc-tcp-socket return
  NS3\_SOCK\_STREAM. udp-socket returns NS3\_SOCK\_DGRAM.
- **BulkSendApplication**

  Sends data as fast as possible up to MaxBytes or unlimited if MaxBytes is
  zero. Think OnOff, but without the "off" and without the variable data rate.
  This application only works with NS3\_SOCK\_STREAM and NS3\_SOCK\_SEQPACKET sockets,
  for example TCP sockets and not UDP sockets. A helper class exists to
  facilitate creating BulkSendApplications. The API for the helper class
  is similar to existing application helper classes, for example, OnOff.
- **Rakhmatov Vrudhula non-linear battery model**

  New class and helper for this battery model.
- **Print IPv4 routing tables**

  New class methods and helpers for printing IPv4 routing tables
  to an output stream.
- **Destination-Sequenced Distance Vector (DSDV) routing protocol**

  Derives from Ipv4RoutingProtocol and contains a DsdvHelper class.
- **3GPP Long Term Evolution (LTE) models**

  More details are in the RELEASE\_NOTES.

## Changes to existing API:

- **Consolidation of Wi-Fi MAC high functionality**

  Wi-Fi MAC high classes have been reorganised in attempt to
  consolidate shared functionality into a single class. This new class
  is RegularWifiMac, and it derives from the abstract WifiMac, and is
  parent of AdhocWifiMac, StaWifiMac, ApWifiMac, and
  MeshWifiInterfaceMac. The QoS and non-QoS class variants are no
  longer, with a RegularWifiMac attribute "QosSupported" allowing
  selection between these two modes of operation. QosWifiMacHelper and
  NqosWifiMacHelper continue to work as previously, with a
  behind-the-scenes manipulation of the 'afore-mentioned attribute.
- **New TCP architecture**

  TcpSocketImpl was replaced by a new base class TcpSocketBase and
  several subclasses implementing different congestion control. From
  a user-level API perspective, the main change is that a new attribute
  "SocketType" is available in TcpL4Protocol, to which a TypeIdValue
  of a specific Tcp variant can be passed. In the same class, the attribute
  "RttEstimatorFactory" was also renamed "RttEstimatorType" since it now
  takes a TypeIdValue instead of an ObjectFactoryValue. In most cases,
  however, no change to existing user programs should be needed.

## Changed behavior:

- **EmuNetDevice uses DIX instead of LLC encapsulation by default**

  bug 984 in ns-3 tracker: real devices don't usually understand LLC/SNAP
  so the default of DIX makes more sense.
- **TCP defaults to NewReno congestion control**

  As part of the TCP socket refactoring, a new TCP implementation provides
  slightly different behavior than the previous TcpSocketImpl that provided
  only fast retransmit. The default behavior now is NewReno which provides
  fast retransmit and fast recovery with window inflation during recovery.

---

# Changes from ns-3.8 to ns-3.9

## Changes to build system:

## New API:

- **Wifi set block ack threshold:** Two methods for setting block ack
  parameters for a specific access class:

  ```
  void QosWifiMacHelper::SetBlockAckThresholdForAc (enum AccessClass accessClass, uint8_t threshold);
  void QosWifiMacHelper::SetBlockAckInactivityTimeoutForAc (enum AccessClass accessClass, uint16_t timeout);
  ```
- **Receive List Error Model:** Another basic error model that allows
  the user to specify a list of received packets that should be errored. The
  list corresponds not to the packet UID but to the sequence of received
  packets as observed by the error model. See src/common/error-model.h
- **Respond to interface events:** New attribute for Ipv4GlobalRouting,
  "RespondToInterfaceEvents", which when enabled, will cause global routes
  to be recomputed upon any interface or address notification event from IPv4.
- **Generic sequence number:** New generic sequence number class to
  easily handle comparison, subtraction, etc. for sequence numbers.
  To use it you need to supply two fundamental types as template parameters:
  NUMERIC\_TYPE and SIGNED\_TYPE. For instance, SequenceNumber<uint32\_t, int32\_t>
  gives you a 32-bit sequence number, while SequenceNumber<uint16\_t, int16\_t>
  is a 16-bit one. For your convenience, these are typedef'ed as
  SequenceNumber32 and SequenceNumber16, respectively.
- **Broadcast socket option:** New Socket
  methods SetAllowBroadcast and GetAllowBroadcast add
  to NS-3 Socket's the equivalent to the POSIX SO\_BROADCAST
  socket option (setsockopt/getsockopt). Starting from this NS-3
  version, IPv4 sockets do not allow us to send packets to broadcast
  destinations by default; SetAllowBroadcast must be called beforehand
  if we wish to send broadcast packets.
- **Deliver of packet ancillary information to sockets:** A method to deliver ancillary information
  to the socket interface (fixed in bug 671):

  ```
  void Socket::SetRecvPktInfo (bool flag);
  ```

## Changes to existing API:

- **Changes to construction and naming of Wi-Fi transmit rates:**
  A reorganisation of the construction of Wi-Fi transmit rates has been
  undertaken with the aim of simplifying the task of supporting further
  IEEE 802.11 PHYs. This work has been completed under the auspices of
  Bug 871.
  From the viewpoint of simulation scripts not part of the ns-3
  distribution, the key change is that WifiMode names of the form
  wifi*x*-*n*mbs are now invalid. Names now take the
  form *Cccc*Rate*n*Mbps[BW*b*MHz],
  where *n* is the root bitrate in megabits-per-second as before
  (with only significant figures included, and an underscore replacing
  any decimal point), and *Cccc* is a representation of the
  Modulation Class as defined in Table 9-2 of IEEE
  Std. 802.11-2007. Currently-supported options for *Cccc*
  are *Ofdm* and *Dsss*. For modulation classes where
  optional reduced-bandwidth transmission is possible, this is captured
  in the final part of the form above, with *b* specifying the
  nominal signal bandwidth in megahertz.
- **Consolidation of classes support Wi-Fi Information Elements:**
  When the *mesh* module was introduced it added a class
  hierarchy for modelling of the various Information Elements that were
  required. In this release, this class hierarchy has extended by moving
  the base classes (WifiInformationElement and
  WifiInformationElementVector) into the *wifi* module. This
  change is intended to ease the addition of support for modelling of
  further Wi-Fi functionality.
- **Changed for {Ipv4,Ipv6}PacketInfoTag delivery:** In order to
  deliver ancillary information to the socket interface (fixed in bug 671),
  *Ipv4PacketInfoTag* and *Ipv6PacketInfoTag* are implemented.
  For the delivery of this information, the following changes are made into
  existing class.
  In Ipv4EndPoint class,

  ```
  -  void SetRxCallback (Callback<void,Ptr<Packet>, Ipv4Address, Ipv4Address, uint16_t> callback);
  +  void SetRxCallback (Callback<void,Ptr<Packet>, Ipv4Header, uint16_t, Ptr<Ipv4Interface> > callback);

  -  void ForwardUp (Ptr<Packet> p, Ipv4Address saddr, Ipv4Address daddr, uint16_t sport);
  +  void ForwardUp (Ptr<Packet> p, const Ipv4Header& header, uint16_t sport, 
  +                  Ptr<Ipv4Interface> incomingInterface);
  ```

  In Ipv4L4Protocol class,

  ```
     virtual enum RxStatus Receive(Ptr<Packet> p, 
  -                                Ipv4Address const &source,
  -                                Ipv4Address const &destination,
  +                                Ipv4Header const &header,
                                   Ptr<Ipv4Interface> incomingInterface) = 0;
  ```

  ```
  -Ipv4RawSocketImpl::ForwardUp (Ptr<const Packet> p, Ipv4Header ipHeader, Ptr<NetDevice> device)
  +Ipv4RawSocketImpl::ForwardUp (Ptr<const Packet> p, Ipv4Header ipHeader, Ptr<Ipv4Interface> incomingInterface)

  -NscTcpSocketImpl::ForwardUp (Ptr<Packet> packet, Ipv4Address saddr, Ipv4Address daddr, uint16_t port)
  +NscTcpSocketImpl::ForwardUp (Ptr<Packet> packet, Ipv4Header header, uint16_t port,
  +                             Ptr<Ipv4Interface> incomingInterface)

  -TcpSocketImpl::ForwardUp (Ptr<Packet> packet, Ipv4Address saddr, Ipv4Address daddr, uint16_t port)
  +TcpSocketImpl::ForwardUp (Ptr<Packet> packet, Ipv4Header header, uint16_t port,
  +                          Ptr<Ipv4Interface> incomingInterface)

  -UdpSocketImpl::ForwardUp (Ptr<Packet> packet, Ipv4Address saddr, Ipv4Address daddr, uint16_t port)
  +UdpSocketImpl::ForwardUp (Ptr<Packet> packet, Ipv4Header header, uint16_t port,
  +                          Ptr<Ipv4Interface> incomingInterface)
  ```
- The method OutputStreamWrapper::SetStream (std::ostream \*ostream) was removed.
)

## Changed behavior:

- **Queue trace behavior during Enqueue changed:** The behavior of the
  Enqueue trace source has been changed to be more intuitive and to agree with
  documentation. Enqueue and Drop events in src/node/queue.cc are now mutually
  exclusive. In the past, the meaning of an Enqueue event was that the Queue
  Enqueue operation was being attempted; and this could be followed by a Drop
  event if the Queue was full. The new behavior is such that a packet is either
  Enqueue'd successfully or Drop'ped.- **Drop trace logged for Ipv4/6 forwarding failure:** Fixed bug 861; this
    will add ascii traces (drops) in Ipv4 and Ipv6 traces for forwarding failures- **Changed default WiFi error rate model for OFDM modulation types:**
      Adopted more conservative ErrorRateModel for OFDM modulation types (a/g).
      This will require 4 to 5 more dB of received power to get similar results
      as before, so users may observe a reduced WiFi range when using the defaults.
      See tracker issue 944 for more details.

---

# Changes from ns-3.7 to ns-3.8

## Changes to build system:

## New API:

- **Matrix propagation loss model:** This radio propagation model uses a two-dimensional matrix
  of path loss indexed by source and destination nodes.- **WiMAX net device**: The developed WiMAX model attempts to provide an accurate MAC and
    PHY level implementation of the 802.16 specification with the Point-to-Multipoint (PMP) mode and the WirelessMAN-OFDM
    PHY layer. By adding WimaxNetDevice objects to ns-3 nodes, one can create models of
    802.16-based networks. The source code for the WiMAX models lives in the directory src/devices/wimax.
    The model is mainly composed of three layers:
    - The convergence sublayer (CS)- The MAC Common Part Sublayer (MAC-CPS)- The Physical layerThe main way that users who write simulation scripts will typically
    interact with the Wimax models is through the helper API and through
    the publicly visible attributes of the model.
    The helper API is defined in src/helper/wimax-helper.{cc,h}.
    Three examples containing some code that shows how to setup a 802.16 network are located under examples/wimax/- **MPI Interface for distributed simulation:** Enables access
      to necessary MPI information such as MPI rank and size.- **Point-to-point remote channel:** Enables point-to-point
        connection between net-devices on different simulators, for use
        with distributed simulation.- **GetSystemId in simulator:** For use with distributed
          simulation, GetSystemId returns zero by non-distributed
          simulators. For the distributed simulator, it returns the
          MPI rank.- **Enhancements to src/core/random-variable.cc/h:** New Zeta random variable generator. The Zeta random
            distribution is tightly related to the Zipf distribution (already in ns-3.7). See the documentation,
            especially because sometimes the Zeta distribution is called Zipf and viceversa. Here we conform to the
            Wikipedia naming convention, i.e., Zipf is bounded while Zeta isn't.- **Two-ray ground propagation loss model:** Calculates the crossover distance under which Friis is used. The antenna
              height is set to the nodes z coordinate, but can be added to using the model parameter SetHeightAboveZ, which
              will affect ALL stations- **Pareto random variable** has two new constructors to specify scale and shape:

                ```
                ParetoVariable (std::pair params);
                ParetoVariable (std::pair params, double b);
                ```

## Changes to existing API:

- **Tracing Helpers**: The organization of helpers for both pcap and ascii
  tracing, in devices and protocols, has been reworked. Instead of each device
  and protocol helper re-implementing trace enable methods, classes have been
  developed to implement user-level tracing in a consistent way; and device and
  protocol helpers use those classes to provide tracing functionality.  
  In addition to consistent operation across all helpers, the object name service
  has been integrated into the trace file naming scheme.  
  The internet stack helper has been extensively massaged to make it easier to
  manage traces originating from protocols. It used to be the case that there
  was essentially no opportunity to filter tracing on interfaces, and resulting
  trace file names collided with those created by devices. File names are now
  disambiguated and one can enable traces on a protocol/interface basis analogously
  to the node/device granularity of device-based helpers.  
  The primary user-visible results of this change are that trace-related functions
  have been changed from static functions to method calls; and a new object has
  been developed to hold streams for ascii traces.  
  New functionality is present for ascii traces. It is now possible to create
  multiple ascii trace files automatically just as was possible for pcap trace
  files.  
  The implementation of the helper code has been designed also to provide
  functionality to make it easier for sophisticated users to hook traces of
  various kinds and write results to (file) streams.
  Before:

  ```
    CsmaHelper::EnablePcapAll ();

    std::ofstream ascii;
    ascii.open ("csma-one-subnet.tr", std::ios_base::binary | std::ios_base::out);
    CsmaHelper::EnableAsciiAll (ascii);

    InternetStackHelper::EnableAsciiAll (ascii);
  ```

  After:

  ```
    CsmaHelper csmaHelper;
    InternetStackHelper stack;
    csmaHelper.EnablePcapAll ();

    AsciiTraceHelper ascii;
    csma.EnableAsciiAll (ascii.CreateFileStream ("csma-one-subnet.tr"));

    stack.EnableAsciiIpv4All (stream);
  ```

  - **Serialization and Deserialization** in buffer, nix-vector,
    packet-metadata, and packet has been modified to use raw character
    buffers, rather than the Buffer class

    ```
    + uint32_t Buffer::GetSerializedSize (void) const;
    + uint32_t Buffer::Serialize (uint8_t* buffer, uint32_t maxSize) const;
    + uint32_t Buffer::Deserialize (uint8_t* buffer, uint32_t size); 

    - void NixVector::Serialize (Buffer::Iterator i, uint32_t size) const;
    + uint32_t NixVector::Serialize (uint32_t* buffer, uint32_t maxSize) const;
    - uint32_t NixVector::Deserialize (Buffer::Iterator i);
    + uint32_t NixVector::Deserialize (uint32_t* buffer, uint32_t size);

    - void PacketMetadata::Serialize (Buffer::Iterator i, uint32_t size) const;
    + uint32_t PacketMetadata::Serialize (uint8_t* buffer, uint32_t maxSize) const;
    - uint32_t PacketMetadata::Deserialize (Buffer::Iterator i);
    + uint32_t PacketMetadata::Deserialize (uint8_t* buffer, uint32_t size);

    + uint32_t Packet::GetSerializedSize (void) const;
    - Buffer Packet::Serialize (void) const;
    + uint32_t Packet::Serialize (uint8_t* buffer, uint32_t maxSize) const;
    - void Packet::Deserialize (Buffer buffer);
    + Packet::Packet (uint8_t const*buffer, uint32_t size, bool magic);
    ```

    - **PacketMetadata uid** has been changed to a 64-bit value. The
      lower 32 bits give the uid, while the upper 32-bits give the MPI rank
      for distributed simulations. For non-distributed simulations, the
      upper 32 bits are simply zero.

      ```
      - inline PacketMetadata (uint32_t uid, uint32_t size);
      + inline PacketMetadata (uint64_t uid, uint32_t size);
      - uint32_t GetUid (void) const;
      + uint64_t GetUid (void) const;
      - PacketMetadata::PacketMetadata (uint32_t uid, uint32_t size);
      + PacketMetadata::PacketMetadata (uint64_t uid, uint32_t size); 

      - uint32_t Packet::GetUid (void) const;
      + uint64_t Packet::GetUid (void) const;
      ```

      - **Moved propagation models** from src/devices/wifi to src/common- **Moved Mtu attribute from base class NetDevice** This attribute is
          now found in all NetDevice subclasses.

## Changed behavior:


---

# Changes from ns-3.6 to ns-3.7

## Changes to build system:

## New API:

- **Equal-cost multipath for global routing:** Enables quagga's
  equal cost multipath for Ipv4GlobalRouting, and adds an attribute that
  can enable it with random packet distribution policy across equal cost routes.- **Binding sockets to devices:** A method analogous to a SO\_BINDTODEVICE
    socket option has been introduced to class Socket:

    ```
    virtual void Socket::BindToNetDevice (Ptr<NetDevice> netdevice);
    ```

    - **Simulator event contexts**: The Simulator API now keeps track of a per-event
      'context' (a 32bit integer which, by convention identifies a node by its id). Simulator::GetContext
      returns the context of the currently-executing event while Simulator::ScheduleWithContext creates an
      event with a context different from the execution context of the caller. This API is used
      by the ns-3 logging system to report the execution context of each log line.- **Object::DoStart**: Users who need to complete their object setup at the start of a simulation
        can override this virtual method, perform their adhoc setup, and then, must chain up to their parent.- **Ad hoc On-Demand Distance Vector (AODV)** routing model,
          RFC 3561
        - **Ipv4::IsDestinationAddress (Ipv4Address address, uint32\_t iif)** Method added to support checks of whether a destination address should be accepted
          as one of the host's own addresses. RFC 1122 Strong/Weak end system behavior can be changed with a new attribute (WeakEsModel) in class Ipv4.
        - **Net-anim interface**: Provides an interface to net-anim, a network animator for point-to-point
          links in ns-3. The interface generates a custom trace file for use with the NetAnim program.
        - **Topology Helpers**: New topology helpers have been introduced including PointToPointStarHelper,
          PointToPointDumbbellHelper, PointToPointGridHelper, and CsmaStarHelper.
        - **IPv6 extensions support**: Provides API to add IPv6 extensions and options. Two examples (fragmentation
          and loose routing) are available.

## Changes to existing API:

- **Ipv4RoutingProtocol::RouteOutput** no longer takes an outgoing
  interface index but instead takes an outgoing device pointer; this affects all
  subclasses of Ipv4RoutingProtocol.

  ```
  -  virtual Ptr<Ipv4Route> RouteOutput (Ptr<Packet> p, const Ipv4Header &header, uint32_t oif, Socket::SocketErrno &sockerr) = 0;
  +  virtual Ptr<Ipv4Route> RouteOutput (Ptr<Packet> p, const Ipv4Header &header, Ptr<NetDevice> oif, Socket::SocketErrno &sockerr) = 0;
  ```

  - **Ipv6RoutingProtocol::RouteOutput** no longer takes an outgoing
    interface index but instead takes an outgoing device pointer; this affects all
    subclasses of Ipv6RoutingProtocol.

    ```
    -  virtual Ptr<Ipv6Route> RouteOutput (Ptr<Packet> p, const Ipv6Header &header, uint32_t oif, Socket::SocketErrno &sockerr) = 0;
    +  virtual Ptr<Ipv6Route> RouteOutput (Ptr<Packet> p, const Ipv6Header &header, Ptr<NetDevice> oif, Socket::SocketErrno &sockerr) = 0;
    ```

    - **Application::Start** and **Application::Stop** have been renamed to
      **Application::SetStartTime** and **Application::SetStopTime**.- **Channel::Send**: this method does not really exist but each subclass of the Channel
        base class must implement a similar method which sends a packet from a node to another node.
        Users must now use Simulator::ScheduleWithContext instead of Simulator::Schedule to schedule
        the reception event on a remote node.  
        For example, before:

        ```
        void
        SimpleChannel::Send (Ptr<Packet> p, uint16_t protocol, 
                 Mac48Address to, Mac48Address from,
                 Ptr<SimpleNetDevice> sender)
        {
          for (std::vector<Ptr<SimpleNetDevice> >::const_iterator i = m_devices.begin (); i != m_devices.end (); ++i)
            {
              Ptr<SimpleNetDevice> tmp = *i;
              if (tmp == sender)
          {
            continue;
          }
              Simulator::ScheduleNow (&SimpleNetDevice::Receive, tmp, p->Copy (), protocol, to, from);
            }
        }
        ```

        After:

        ```
        void
        SimpleChannel::Send (Ptr<Packet> p, uint16_t protocol, 
                 Mac48Address to, Mac48Address from,
                 Ptr<SimpleNetDevice> sender)
        {
          for (std::vector<Ptr<SimpleNetDevice> >::const_iterator i = m_devices.begin (); i != m_devices.end (); ++i)
            {
              Ptr<SimpleNetDevice> tmp = *i;
              if (tmp == sender)
          {
            continue;
          }
              Simulator::ScheduleWithContext (tmp->GetNode ()->GetId (), Seconds (0),
                                              &SimpleNetDevice::Receive, tmp, p->Copy (), protocol, to, from);
            }
        }
        ```

        - **Simulator::SetScheduler**: this method now takes an ObjectFactory
          instead of an object pointer directly. Existing callers can trivially be
          updated to use this new method.  
          Before:

          ```
          Ptr<Scheduler> sched = CreateObject<ListScheduler> ();
          Simulator::SetScheduler (sched);
          ```

          After:

          ```
          ObjectFactory sched;
          sched.SetTypeId ("ns3::ListScheduler");
          Simulator::SetScheduler (sched);
          ```

          - Extensions to IPv4 **Ping** application: verbose output and the ability to configure different ping
            sizes and time intervals (via new attributes)- **Topology Helpers**: Previously, topology helpers such as a point-to-point star existed in the
              PointToPointHelper class in the form of a method (ex: PointToPointHelper::InstallStar). These topology
              helpers have been pulled out of the specific helper classes and created as separate classes. Several
              different topology helper classes now exist including PointToPointStarHelper, PointToPointGridHelper,
              PointToPointDumbbellHelper, and CsmaStarHelper. For example, a user wishes to create a
              point-to-point star network:  
              Before:

              ```
              NodeContainer hubNode;
              NodeContainer spokeNodes;
              hubNode.Create (1);
              Ptr<Node> hub = hubNode.Get (0);
              spokeNodes.Create (nNodes - 1);

              PointToPointHelper pointToPoint;
              pointToPoint.SetDeviceAttribute ("DataRate", StringValue ("5Mbps"));
              pointToPoint.SetChannelAttribute ("Delay", StringValue ("2ms"));
              NetDeviceContainer hubDevices, spokeDevices;
              pointToPoint.InstallStar (hubNode.Get (0), spokeNodes, hubDevices, spokeDevices);
              ```

              After:

              ```
              PointToPointHelper pointToPoint;
              pointToPoint.SetDeviceAttribute ("DataRate", StringValue ("5Mbps"));
              pointToPoint.SetChannelAttribute ("Delay", StringValue ("2ms"));
              PointToPointStarHelper star (nSpokes, pointToPoint);
              ```

## Changed behavior:

- Changed default value of YansWifiPhy::EnergyDetectionThreshold from
  -140.0 dBm to -96.0 dBm. Changed default value of
  YansWifiPhy::CcaModelThreshold from -140.0 dBm to -99.0 dBm. Rationale
  can be found 
  here.
- Default TTL of IPv4 broadcast datagrams changed from 1 to 64.
- Changed DcfManager::UpdateBackoff (): using flooring instead of rounding in calculation of remaining slots. 
  See bug 695.

---

# Changes from ns-3.5 to ns-3.6

## Changes to build system:

- **A new test framework is provided with ns-3.6 that primarilay runs outside waf**

  "./waf check" now runs the new unit tests of the core part of ns-3.6.
  In order to run the complete test package, use "./test.py" which is
  documented in a new manual -- find it in ./doc/testing. "./waf check"
  no longer generates the introspected Doxygen. Now use "./waf doxygen"
  to do this and generate the Doxygen documentation in one step.

## New API:

- **Longest prefix match, support for metrics, for Ipv4StaticRouting and Ipv6StaticRouting**

  When performing route lookup, first match for longest prefix, and then
  based on metrics (default metric = 0). If metrics are equal, most recent
  addition is picked. Extends API for support of metrics but preserves
  backward compatibility. One small change is that the default route
  is no longer stored as index 0 route in the host route table so
  GetDefaultRoute () must be used.
- **Route injection for global routing**

  Add ability to inject and withdraw routes to Ipv4GlobalRouting. This
  allows a user to insert a route and have it redistributed like an OSPF
  external LSA to the rest of the topology.
- **Athstats**

  New classes AthstatsWifiTraceSink and AthstatsHelper.
- **WifiRemoteStationManager** 

  New trace sources exported by WifiRemoteStationManager: MacTxRtsFailed, MacTxDataFailed, MacTxFinalRtsFailed and MacTxFinalDataFailed.
- **IPv6 additions**

  Add an IPv6 protocol and ICMPv6 capability.
  - new classes Ipv6, Ipv6Interface, Ipv6L3Protocol, Ipv6L4Protocol- Ipv6RawSocket (no UDP or TCP capability yet)- a set of classes to implement Icmpv6, including neighbor discovery,
        router solicitation, DAD- new applications Ping6 and Radvd- routing objects Ipv6Route and Ipv6MulticastRoute- routing protocols Ipv6ListRouting and Ipv6StaticRouting- examples: icmpv6-redirect.cc, ping6.cc, radvd.cc, radvd-two-prefix.cc, simple-routing-ping6.cc
- **Wireless Mesh Networking models**


  - General multi-interface mesh stack infrastructure (devices/mesh module).- IEEE 802.11s (Draft 3.0) model including Peering Management Protocol and HWMP.- Forwarding Layer for Meshing (FLAME) protocol.
- **802.11 enhancements**

  - 10MHz and 5MHz channel width supported by 802.11a model (Ramon Bauza and Kirill Andreev).
  - Channel switching support. YansWifiPhy can now switch among different channels (Ramon Bauza and Pavel Boyko).
- **Nix-vector Routing**

  Add nix-vector routing protocol
  - new helper class Ipv4NixVectorHelper
  - examples: nix-simple.cc, nms-p2p-nix.cc
- **New Test Framework**

  Add TestCase, TestSuite classes
  - examples: src/core/names-test-suite.cc, src/core/random-number-test-suite.cc, src/test/ns3tcp/ns3tcp-cwnd-test-suite.cc

## Changes to existing API:

- **InterferenceHelper**

  The method InterferenceHelper::CalculateTxDuration (uint32\_t size, WifiMode payloadMode, WifiPreamble preamble) has been made static, so that the frame duration depends only on the characteristics of the frame (i.e., the function parameters) and not on the particular standard which is used by the receiving PHY. This makes it now possible to correctly calculate the duration of incoming frames in scenarios in which devices using different PHY configurations coexist in the same channel (e.g., a BSS using short preamble and another BSS using long preamble).

  The following member methods have been added to InterferenceHelper:

  ```
    static WifiMode GetPlcpHeaderMode (WifiMode, WifiPreamble);
    static uint32_t GetPlcpHeaderDurationMicroSeconds (WifiMode, WifiPreamble);
    static uint32_t GetPlcpPreambleDurationMicroSeconds (WifiMode, WifiPreamble);
    static uint32_t GetPayloadDurationMicroSeconds (size, WifiMode);
  ```

  The following member methods have been removed from InterferenceHelper:

  ```
    void Configure80211aParameters (void);
    void Configure80211bParameters (void);
    void Configure80211_10MhzParameters (void);
    void Configure80211_5MhzParameters (void);
  ```
- **WifiMode**

  WifiMode now has a WifiPhyStandard attribute which identifies the standard the WifiMode belongs to. To properly set this attribute when creating a new WifiMode, it is now required to explicitly pass a WifiPhyStandard parameter to all WifiModeFactory::CreateXXXX() methods. The WifiPhyStandard value of an existing WifiMode can be retrieved using the new method WifiMode::GetStandard().
- **NetDevice**

  In order to have multiple link change callback in NetDevice (i.e. to flush ARP and IPv6 neighbor discovery caches), the following member method has been renamed:

  ```
  - virtual void SetLinkChangeCallback (Callback<void> callback);
  + virtual void AddLinkChangeCallback (Callback<void> callback);
  ```

  Now each NetDevice subclasses have a TracedCallback<> object (list of callbacks) instead of Callback<void> ones.

---

# Changes from ns-3.4 to ns-3.5

## Changes to build system:


## New API:

- **YansWifiPhyHelper supporting radiotap and prism PCAP output**

  The newly supported pcap formats can be adopted by calling the following new method of YansWifiPhyHelper:

  ```
   +  void SetPcapFormat (enum PcapFormat format);
  ```

  where format is one of PCAP\_FORMAT\_80211\_RADIOTAP, PCAP\_FORMAT\_80211\_PRISM or PCAP\_FORMAT\_80211. By default, PCAP\_FORMAT\_80211 is used, so the default PCAP format is the same as before.
- **attributes for class Ipv4**

  class Ipv4 now contains attributes in ipv4.cc; the first one
  is called "IpForward" that will enable/disable Ipv4 forwarding.
- **packet tags**

  class Packet now contains AddPacketTag, RemovePacketTag and PeekPacketTag
  which can be used to attach a tag to a packet, as opposed to the old
  AddTag method which attached a tag to a set of bytes. The main
  semantic difference is in how these tags behave in the presence of
  fragmentation and reassembly.

## Changes to existing API:

- **Ipv4Interface::GetMtu () deleted**

  The Ipv4Interface API is private to internet-stack module; this method
  was just a pass-through to GetDevice ()->GetMtu ().
- **GlobalRouteManager::PopulateRoutingTables () and RecomputeRoutingTables () are deprecated** 

  This API has been moved to the helper API and the above functions will
  be removed in ns-3.6. The new API is:

  ```
  Ipv4GlobalRoutingHelper::PopulateRoutingTables ();
  Ipv4GlobalRoutingHelper::RecomputeRoutingTables ();
  ```

  Additionally, these low-level functions in GlobalRouteManager are now public,
  allowing more API flexibility at the low level ns-3 API:

  ```
  GlobalRouteManager::DeleteGlobalRoutes ();
  GlobalRouteManager::BuildGlobalRoutingDatabase ();
  GlobalRouteManager::InitializeRoutes ();
  ```
- **CalcChecksum attribute changes**

  Four IPv4 CalcChecksum attributes (which enable the computation of
  checksums that are disabled by default) have been collapsed into one global
  value in class Node. These four calls:

  ```
  Config::SetDefault ("ns3::Ipv4L3Protocol::CalcChecksum", BooleanValue (true)); 
  Config::SetDefault ("ns3::Icmpv4L4Protocol::CalcChecksum", BooleanValue (true));
  Config::SetDefault ("ns3::TcpL4Protocol::CalcChecksum", BooleanValue (true));
  Config::SetDefault ("ns3::UdpL4Protocol::CalcChecksum", BooleanValue (true));
  ```

  are replaced by one call to:

  ```
  GlobalValue::Bind ("ChecksumEnabled", BooleanValue (true));
  ```
- **CreateObject changes**

  CreateObject is now able to construct objects with a non-default constructor.
  If you used to pass attribute lists to CreateObject, you must now use CreateObjectWithAttributes.
- **packet byte tags renaming**
  - Packet::AddTag to Packet::AddByteTag
  - Packet::FindFirstMatchingTag to Packet::FindFirstMatchingByteTag
  - Packet::RemoveAllTags to Packet::RemoveAllByteTags
  - Packet::PrintTags to Packet::PrintByteTags
  - Packet::GetTagIterator to Packet::GetByteTagIterator
- **YansWifiPhyHelper::EnablePcap\* methods not static any more**

  To accommodate the possibility of configuring the PCAP format used for wifi promiscuous mode traces, several methods of YansWifiPhyHelper had to be made non-static:

  ```
  -  static void EnablePcap (std::string filename, uint32_t nodeid, uint32_t deviceid);
  +         void EnablePcap (std::string filename, uint32_t nodeid, uint32_t deviceid);
  -  static void EnablePcap (std::string filename, Ptr<NetDevice> nd);
  +         void EnablePcap (std::string filename, Ptr<NetDevice> nd);
  -  static void EnablePcap (std::string filename, std::string ndName);
  +         void EnablePcap (std::string filename, std::string ndName);
  -  static void EnablePcap (std::string filename, NetDeviceContainer d);
  +         void EnablePcap (std::string filename, NetDeviceContainer d);
  -  static void EnablePcap (std::string filename, NodeContainer n);
  +         void EnablePcap (std::string filename, NodeContainer n);
  -  static void EnablePcapAll (std::string filename);
  +         void EnablePcapAll (std::string filename);
  ```
- **Wifi Promisc Sniff interface modified** 

  To accommodate support for the radiotap and prism headers in PCAP traces, the interface for promiscuos mode sniff in the wifi device was changed. The new implementation was heavily inspired by the way the madwifi driver handles monitor mode. A distinction between TX and RX events is introduced, to account for the fact that different information is to be put in the radiotap/prism header (e.g., RSSI and noise make sense only for RX packets). The following are the relevant modifications to the WifiPhy class:

  ```
  -  void NotifyPromiscSniff (Ptr<const Packet> packet);
  +  void NotifyPromiscSniffRx (Ptr<const Packet> packet, uint16_t channelFreqMhz, uint32_t rate, bool isShortPreamble, double signalDbm, double noiseDbm);
  +  void NotifyPromiscSniffTx (Ptr<const Packet> packet, uint16_t channelFreqMhz, uint32_t rate, bool isShortPreamble);
  -  TracedCallback<Ptr<const Packet> > m_phyPromiscSnifferTrace;
  +  TracedCallback<Ptr<const Packet>, uint16_t, uint32_t, bool, double, double> m_phyPromiscSniffRxTrace;
  +  TracedCallback<Ptr<const Packet>, uint16_t, uint32_t, bool> m_phyPromiscSniffTxTrace;
  ```

  The above mentioned callbacks are expected to be used to call the following method to write Wifi PCAP traces in promiscuous mode:

  ```
  +  void WriteWifiMonitorPacket(Ptr<const Packet> packet, uint16_t channelFreqMhz, uint32_t rate, bool isShortPreamble, bool isTx, double signalDbm, double noiseDbm);
  ```

  In the above method, the isTx parameter is to be used to differentiate between TX and RX packets. For an example of how to implement these callbacks, see the implementation of PcapSniffTxEvent and PcapSniffRxEvent in src/helper/yans-wifi-helper.cc
- **Routing decoupled from class Ipv4**

  All calls of the form "Ipv4::AddHostRouteTo ()" etc. (i.e. to
  add static routes, both unicast and multicast) have been moved to a new
  class Ipv4StaticRouting. In addition, class Ipv4 now holds only
  one possible routing protocol; the previous way to add routing protocols
  (by ordered list of priority) has been moved to a new class Ipv4ListRouting.
  Class Ipv4 has a new minimal routing API (just to set and get the routing
  protocol):

  ```
  -  virtual void AddRoutingProtocol (Ptr<Ipv4RoutingProtocol> routingProtocol, int16_t priority) = 0;
  +  virtual void SetRoutingProtocol (Ptr<Ipv4RoutingProtocol> routingProtocol) = 0;
  +  virtual Ptr<Ipv4RoutingProtocol> GetRoutingProtocol (void) const = 0;
  ```
- **class Ipv4RoutingProtocol is refactored**

  The abstract base class Ipv4RoutingProtocol has been refactored to
  align with corresponding Linux Ipv4 routing architecture, and has been
  moved from ipv4.h to a new file ipv4-routing-protocol.h. The new
  methods (RouteOutput () and RouteInput ()) are aligned with Linux
  ip\_route\_output() and ip\_route\_input(). However,
  the general nature of these calls (synchronous routing lookup for
  locally originated packets, and an asynchronous, callback-based lookup
  for forwarded packets) is still the same.

  ```
  -  typedef Callback<void, bool, const Ipv4Route&, Ptr<Packet>, const Ipv4Header&> RouteReplyCallback;
  +  typedef Callback<void, Ptr<Ipv4Route>, Ptr<const Packet>, const Ipv4Header &> UnicastForwardCallback;
  +  typedef Callback<void, Ptr<Ipv4MulticastRoute>, Ptr<const Packet>, const Ipv4Header &> MulticastForwardCallback;
  +  typedef Callback<void, Ptr<const Packet>, const Ipv4Header &, uint32_t > LocalDeliverCallback;
  +  typedef Callback<void, Ptr<const Packet>, const Ipv4Header &> ErrorCallback;
  -  virtual bool RequestInterface (Ipv4Address destination, uint32_t& interface) = 0;
  +  virtual Ptr<Ipv4Route> RouteOutput (Ptr<Packet> p, const Ipv4Header &header, uint32_t oif, Socket::SocketErrno &errno) = 0;
  -  virtual bool RequestRoute (uint32_t interface,
  -                            const Ipv4Header &ipHeader,
  -                            Ptr<Packet> packet,
  -                            RouteReplyCallback routeReply) = 0;
  +  virtual bool RouteInput  (Ptr<const Packet> p, const Ipv4Header &header, Ptr<const NetDevice> idev,
  +                             UnicastForwardCallback ucb, MulticastForwardCallback mcb,
  +                             LocalDeliverCallback lcb, ErrorCallback ecb) = 0;
  ```
- **previous class Ipv4Route, Ipv4MulticastRoute renamed; new classes with
  those same names added**

  The previous class Ipv4Route and Ipv4MulticastRoute are used by
  Ipv4StaticRouting and Ipv4GlobalRouting to record internal routing table
  entries, so they were renamed to class Ipv4RoutingTableEntry and
  Ipv4MulticastRoutingTableEntry, respectively. In their place, new
  class Ipv4Route and class Ipv4MulticastRoute have been added. These
  are reference-counted objects that are analogous to Linux struct
  rtable and struct mfc\_cache, respectively, to achieve better compatibility
  with Linux routing architecture in the future.- **class Ipv4 address-to-interface mapping functions changed**

    There was some general cleanup of functions that involve mappings
    from Ipv4Address to either NetDevice or Ipv4 interface index.

    ```
    -  virtual uint32_t FindInterfaceForAddr (Ipv4Address addr) const = 0;
    -  virtual uint32_t FindInterfaceForAddr (Ipv4Address addr, Ipv4Mask mask) const = 0;
    +  virtual int32_t GetInterfaceForAddress (Ipv4Address address) const = 0;
    +  virtual int32_t GetInterfaceForPrefix (Ipv4Address address, Ipv4Mask mask) const = 0;
    -  virtual int32_t FindInterfaceForDevice(Ptr<NetDevice> nd) const = 0;
    +  virtual int32_t GetInterfaceForDevice (Ptr<const NetDevice> device) const = 0;
    -  virtual Ipv4Address GetSourceAddress (Ipv4Address destination) const = 0;
    -  virtual bool GetInterfaceForDestination (Ipv4Address dest,
    -  virtual uint32_t GetInterfaceByAddress (Ipv4Address addr, Ipv4Mask mask = Ipv4Mask("255.255.255.255"));
    ```

    - **class Ipv4 multicast join API deleted**

      The following methods are not really used in present form since IGMP
      is not being generated, so they have been removed (planned to be replaced
      by multicast socket-based calls in the future):

      ```
      - virtual void JoinMulticastGroup (Ipv4Address origin, Ipv4Address group) = 0;
      - virtual void LeaveMulticastGroup (Ipv4Address origin, Ipv4Address group) = 0;
      ```

      - **Deconflict NetDevice::ifIndex and Ipv4::ifIndex (bug 85).**

        All function parameters named "ifIndex" that refer
        to an Ipv4 interface are instead named "interface".

        ```
        - static const uint32_t Ipv4RoutingProtocol::IF_INDEX_ANY = 0xffffffff;
        + static const uint32_t Ipv4RoutingProtocol::INTERFACE_ANY = 0xffffffff;

        - bool Ipv4RoutingProtocol::RequestIfIndex (Ipv4Address destination, uint32_t& ifIndex);
        + bool Ipv4RoutingProtocol::RequestInterface (Ipv4Address destination, uint32_t& interface);
        (N.B. this particular function is planned to be renamed to RouteOutput() in the
        proposed IPv4 routing refactoring)

        - uint32_t Ipv4::GetIfIndexByAddress (Ipv4Address addr, Ipv4Mask mask);
        + int_32t Ipv4::GetInterfaceForAddress (Ipv4Address address, Ipv4Mask mask) const;

        - bool Ipv4::GetIfIndexForDestination (Ipv4Address dest, uint32_t &ifIndex) const;
        + bool Ipv4::GetInterfaceForDestination (Ipv4Address dest, uint32_t &interface) const;
        (N.B. this function is not needed in the proposed Ipv4 routing refactoring)
        ```

        - **Allow multiple IPv4 addresses to be assigned to an interface (bug 188)**
          - Add class Ipv4InterfaceAddress:
            This is a new class to resemble Linux's struct in\_ifaddr. It holds IP addressing information, including mask,
            broadcast address, scope, whether primary or secondary, etc.

            ```
            +  virtual uint32_t AddAddress (uint32_t interface, Ipv4InterfaceAddress address) = 0;
            +  virtual Ipv4InterfaceAddress GetAddress (uint32_t interface, uint32_t addressIndex) const = 0;
            +  virtual uint32_t GetNAddresses (uint32_t interface) const = 0;
            ```

            - Regarding legacy API usage, typically where you once did the following,
              using the public Ipv4 class interface (e.g.):

              ```
                ipv4A->SetAddress (ifIndexA, Ipv4Address ("172.16.1.1"));
                ipv4A->SetNetworkMask (ifIndexA, Ipv4Mask ("255.255.255.255"));
              ```

              you now do:

              ```
                Ipv4InterfaceAddress ipv4IfAddrA = Ipv4InterfaceAddress (Ipv4Address ("172.16.1.1"), Ipv4Mask ("255.255.255.255"));
                ipv4A->AddAddress (ifIndexA, ipv4IfAddrA);
              ```

              - At the helper API level, one often gets an address from an interface
                container. We preserve the legacy GetAddress (uint32\_t i) but it
                is documented that this will return only the first (address index 0)
                address on the interface, if there are multiple such addresses.
                We provide also an overloaded variant for the multi-address case:

                ```
                Ipv4Address Ipv4InterfaceContainer::GetAddress (uint32_t i)
                + Ipv4Address Ipv4InterfaceContainer::GetAddress (uint32_t i, uint32_t j)
                ```- **New WifiMacHelper objects**

            The type of wifi MAC is now set by two new specific helpers, NqosWifiMacHelper for non QoS MACs and QosWifiMacHelper for Qos MACs. They are passed as argument to WifiHelper::Install methods.

          ```
          - void WifiHelper::SetMac (std::string type, std::string n0 = "", const AttributeValue &v0 = EmptyAttributeValue (),...)

          - NetDeviceContainer WifiHelper::Install (const WifiPhyHelper &phyHelper, NodeContainer c) const
          + NetDeviceContainer WifiHelper::Install (const WifiPhyHelper &phyHelper, const WifiMacHelper &macHelper, NodeContainer c) const

          - NetDeviceContainer WifiHelper::Install (const WifiPhyHelper &phy, Ptr<Node> node) const
          + NetDeviceContainer WifiHelper::Install (const WifiPhyHelper &phy, const WifiMacHelper &mac, Ptr<Node> node) const

          - NetDeviceContainer WifiHelper::Install (const WifiPhyHelper &phy, std::string nodeName) const
          + NetDeviceContainer WifiHelper::Install (const WifiPhyHelper &phy, const WifiMacHelper &mac, std::string nodeName) const
          ```

          See src/helper/nqos-wifi-mac-helper.h and src/helper/qos-wifi-mac-helper.h for more details.

        - **Remove Mac48Address::IsMulticast**

          This method was considered buggy and unsafe to call. Its replacement is Mac48Address::IsGroup.

## Changed behavior:


---

# Changes from ns-3.3 to ns-3.4

## Changes to build system:

- A major option regarding the downloading and building of ns-3 has been
  added for ns-3.4 -- the ns-3-allinone feature. This allows a user to
  get the most common options for ns-3 downloaded and built with a minimum
  amount of trouble. See the ns-3 tutorial for a detailed explanation of
  how to use this new feature.
- The build system now runs build items in parallel by default. This includes
  the regression tests.

## New API:

- XML support has been added to the ConfigStore in src/contrib/config-store.cc
- The ns-2 calendar queue scheduler option has been ported to src/simulator
- A ThreeLogDistancePropagationLossModel has been added to src/devices/wifi
- ConstantAccelerationMobilityModel in src/mobility/constant-acceleration-mobility-model.h
- A new emulation mode is supported with the TapBridge net device (see
  src/devices/tap-bridge)
- A new facility for naming ns-3 Objects is included (see
  src/core/names.{cc,h})
- Wifi multicast support has been added in src/devices/wifi

## Changes to existing API:

- Some fairly significant changes have been made to the API of the
  random variable code. Please see the ns-3 manual and src/core/random-variable.cc
  for details.
- The trace sources in the various NetDevice classes has been completely
  reworked to allow for a consistent set of trace sources across the
  devices. The names of the trace sources have been changed to provide
  some context with respect to the level at which the trace occurred.
  A new set of trace sources has been added which emulates the behavior
  of packet sniffers. These sources have been used to implement tcpdump-
  like functionality and are plumbed up into the helper classes. The
  user-visible changes are the trace source name changes and the ability
  to do promiscuous-mode pcap tracing via helpers. For further information
  regarding these changes, please see the ns-3 manual
- StaticMobilityModel has been renamed ConstantPositionMobilityModel
  StaticSpeedMobilityModel has been renamed ConstantVelocityMobilityModel
- The Callback templates have been extended to support more parameters.
  See src/core/callback.h
- Many helper API have been changed to allow passing Object-based parameters
  as string names to ease working with the object name service.
- The Config APIs now accept path segments that are names defined by the
  object name service.
- Minor changes were made to make the system build under the Intel C++ compiler.
- Trace hooks for association and deassociation to/from an access point were
  added to src/devices/wifi/nqsta-wifi-mac.cc

## Changed behavior:

- The tracing system rework has introduced some significant changes in the
  behavior of some trace sources, specifically in the positioning of trace sources
  in the device code. For example, there were cases where the packet transmit
  trace source was hit before the packet was enqueued on the device transmit quueue.
  This now happens just before the packet is transmitted over the channel medium.
  The scope of the changes is too large to be included here. If you have concerns
  regarding trace semantics, please consult the net device documentation for details.
  As is usual, the ultimate source for documentation is the net device source code.

---

# Changes from ns-3.2 to ns-3.3

## New API:

- ns-3 ABORT macros in src/core/abort.h
  Config::MatchContainer
  ConstCast and DynamicCast helper functions for Ptr casting
  StarTopology added to several topology helpers
  NetDevice::IsBridge ()
- 17-11-2008; changeset
  4c1c3f6bcd03

- The PppHeader previously defined in the point-to-point-net-device code has been
  made public.

- 17-11-2008; changeset
  16c2970a0344

- An emulated net device has been added as enabling technology for ns-3 emulation
  scenarios. See src/devices/emu and examples/emu-udp-echo.cc for details.

- 17-11-2008; changeset
  4222173d1e6d

- Added method InternetStackHelper::EnableAsciiChange to allow allow a user to
  hook ascii trace to the drop trace events in Ipv4L3Protocol and ArpL3Protocol.

## Changes to existing API:

- NetDevice::MakeMulticastAddress() was renamed to NetDevice::GetMulticast()
  and the original GetMulticast() removed
- Socket API changes:
  - return type of SetDataSentCallback () changed from bool to void
  - Socket::Listen() no longer takes a queueLimit argument- As part of the Wifi Phy rework, there have been several API changes
    at the low level and helper API level.
  - At the helper API level, the WifiHelper was split to three classes:
    a WifiHelper, a YansWifiChannel helper, and a YansWifiPhy helper. Some
    functions like Ascii and Pcap tracing functions were moved from class
    WifiHelper to class YansWifiPhyHelper.- At the low-level API, there have been a number of changes to
      make the Phy more modular:
    - composite-propagation-loss-model.h is removed
    - DcfManager::NotifyCcaBusyStartNow() has changed name
    - fragmentation related functions (e.g. DcaTxop::GetNFragments()) have
      changed API to account for some implementation changes
    - Interference helper and error rate model added
    - JakesPropagationLossModel::GetLoss() moved to PropagationLoss() class
    - base class WifiChannel made abstract
    - WifiNetDevice::SetChannel() removed
    - a WifiPhyState helper class added
    - addition of the YansWifiChannel and YansWifiPhy classes- 17-11-2008; changeset
    dacfd1f07538
  - Change attribute "RxErrorModel" to "ReceiveErrorModel" in CsmaNetDevice for
    consistency between devices.

## changed behavior:

- 17-11-2008; changeset
  ed0dfce40459

- Relax reasonableness testing in Ipv4AddressHelper::SetBase to allow the
  assignment of /32 addresses.

- 17-11-2008; changeset
  756887a9bbea

- Global routing supports bridge devices.

---

# Changes from ns-3.1 to ns-3.2

## New API:

- 26-08-2008; changeset
  5aa65b1ea001

- Add multithreaded and real-time simulator implementation. Allows for emulated
  net devices running in threads other than the main simulation thread to schedule
  events. Allows for pacing the simulation clock at 1x real-time.

- 26-08-2008; changeset
  c69779f5e51e

- Add threading and synchronization primitives. Enabling technology for
  multithreaded simulator implementation.

## New API in existing classes:

- 01-08-2008; changeset
  a18520551cdf

- class ArpCache has two new attributes: MaxRetries
  and a Drop trace. It also has some new public methods but these are
  mostly for internal use.

## Changes to existing API:

- 05-09-2008; changeset
  aa1fb0f43571

- Change naming of MTU and packet size attributes in CSMA and Point-to-Point devices  
  After much discussion it was decided that the preferred way to think about
  the different senses of transmission units and encapsulations was to call the
  MAC MTU simply MTU and to use the overall packet size as the PHY-level attribute
  of interest. See the Doxygen of CsmaNetDevice::SetFrameSize and
  PointToPointNetDevice::SetFrameSize for a detailed description.

- 25-08-2008; changeset
  e5ab96db540e

- bug 273: constify packet pointers.  
  The normal and the promiscuous receive callbacks of the NetDevice API
  have been changed from:

  ```
  Callback<bool,Ptr<NetDevice>,Ptr<Packet>,uint16_t,const Address &>
  Callback<bool,Ptr<NetDevice>, Ptr<Packet>, uint16_t,
           const Address &, const Address &, enum PacketType >
  ```

  to:

  ```
  Callback<bool,Ptr<NetDevice>,Ptr<const Packet>,uint16_t,const Address &>
  Callback<bool,Ptr<NetDevice>, Ptr<const Packet>, uint16_t,
           const Address &, const Address &, enum PacketType >
  ```

  to avoid the kind of bugs reported in
  bug 273.
  Users who implement a subclass of the NetDevice base class need to change the signature
  of their SetReceiveCallback and SetPromiscReceiveCallback methods.

- 04-08-2008; changeset
  cba7b2b80fe8

- Cleanup of MTU confusion and initialization in CsmaNetDevice  
  The MTU of the CsmaNetDevice defaulted to 65535. This did not correspond with
  the expected MTU found in Ethernet-like devices. Also there was not clear
  documentation regarding which MTU was being set. There are two MTU here, one
  at the MAC level and one at the PHY level. We split out the MTU setting to make
  this more clear and set the default PHY level MTU to 1500 to be more like
  Ethernet. The encapsulation mode defaults to LLC/SNAP which then puts the
  MAC level MTU at 1492 by default. We allow users to now set the encapsulation
  mode, MAC MTU and PHY MTU while keeping the three values consistent. See the
  Doxygen of CsmaNetDevice::SetMaxPayloadLength for a detailed description of the
  issues and solution.

- 21-07-2008; changeset
  99698bc858e8

- class NetDevice has added a pure virtual method that
  must be implemented by all subclasses:

  ```
  virtual void SetPromiscReceiveCallback (PromiscReceiveCallback cb) = 0;
  ```

  All NetDevices must support this method, and must call this callback
  when processing packets in the receive direction (the appropriate place
  to call this is device-dependent). An approach to stub this out
  temporarily, if you do not care about immediately enabling this
  functionality, would be to add this to your device:

  ```
  void
  ExampleNetDevice::SetPromiscReceiveCallback
  (NetDevice::PromiscReceiveCallback cb)
  { 
    NS_ASSERT_MSG (false, "No implementation yet for
  SetPromiscReceiveCallback");
  }
  ```

  To implement this properly, consult the CsmaNetDevice for examples of
  when the m\_promiscRxCallback is called.

- 03-07-2008; changeset
  d5f8e5fae1c6

- Miscellaneous cleanup of Udp Helper API, to fix bug 234

  ```
  class UdpEchoServerHelper
  {
  public:
  - UdpEchoServerHelper ();
  - void SetPort (uint16_t port); 
  + UdpEchoServerHelper (uint16_t port);
  + 
  + void SetAttribute (std::string name, const AttributeValue &value);
  ApplicationContainer Install (NodeContainer c);

  class UdpEchoClientHelper
  {
  public:
  - UdpEchoClientHelper ();
  + UdpEchoClientHelper (Ipv4Address ip, uint16_t port);
  - void SetRemote (Ipv4Address ip, uint16_t port);
  - void SetAppAttribute (std::string name, const AttributeValue &value);
  + void SetAttribute (std::string name, const AttributeValue &value);
  ApplicationContainer Install (NodeContainer c);
  ```

- 03-07-2008; changeset
  3cdd9d60f7c7

- Rename all instances method names using "Set..Parameter" to "Set..Attribute"
  (bug 232)
- How to fix your code: Any use of helper API that was using a method
  "Set...Parameter()" should be changed to read "Set...Attribute()". e.g.

  ```
  - csma.SetChannelParameter ("DataRate", DataRateValue (5000000));
  - csma.SetChannelParameter ("Delay", TimeValue (MilliSeconds (2)));
  + csma.SetChannelAttribute ("DataRate", DataRateValue (5000000));
  + csma.SetChannelAttribute ("Delay", TimeValue (MilliSeconds (2)));
  ```

## Changed behavior:

- 07-09-2008; changeset
  5d836ab1523b

- Implement a finite receive buffer for TCP  
  The native TCP model in TcpSocketImpl did not support a finite receive buffer.
  This changeset adds the following functionality in this regard:
  - Being able to set the receiver buffer size through the attributes system.
  - This receiver buffer size is now correctly exported in the TCP header as the
    advertised window. Prior to this changeset, the TCP header advertised window
    was set to the maximum size of 2^16 bytes.
    window
  - The aforementioned window size is correctly used for flow control, i.e. the
    sending TCP will not send more data than available space in the receiver's
    buffer.
  - In the case of a receiver window collapse, when a advertised zero-window
    packet is received, the sender enters the persist probing state in which
    it sends probe packets with one payload byte at exponentially backed-off
    intervals up to 60s. The receiver will continue to send advertised
    zero-window ACKs of the old data so long as the receiver buffer remains full.
    When the receiver window clears up due to an application read, the TCP
    will finally ACK the probe byte, and update its advertised window appropriately.See
   bug 239  for
  more.

- 07-09-2008; changeset
  7afa66c2b291

- Add correct FIN exchange behavior during TCP closedown  
  The behavior of the native TcpSocketImpl TCP model was such that the final
  FIN exchange was not correct, i.e. calling Socket::Close didn't send a FIN
  packet, and even if it had, the ACK never came back, and even if it had, the
  ACK would have incorrect sequence number. All these various problems have been
  addressed by this changeset. See
   bug 242  for
  more.

- 28-07-2008; changeset
  6f68f1044df1
  - OLSR: HELLO messages hold time changed to 3\*hello
    interval from hello interval. This is an important bug fix as
    hold time == refresh time was never intentional, as it leads to
    instability in neighbor detection.
