# Supplementary material for: An empirical evaluation of link quality utilization in ETX routing for VANETs
Source: PeerJ Comput Sci. 2024 Sep 6;10:e2259. doi: 10.7717/peerj-cs.2259 (PMC11419655; doi:10.7717/peerj-cs.2259)
Supplement: Supplemental Information 2 [file peerj-cs-10-2259-s002.zip › bonnmotion-3.0.1/doc/README.pdf]

BonnMotion

A Mobility Scenario Generation and Analysis Tool

Documentation

Version: February 18, 2016

Copyright ©2016 University of Osnabrück

Contents

1 Legal notice **4**

2 Contact information **4**

3 Introduction **4**

4 Installation **4**
4.1 Installation on UNIX operating systems . . . 5
4.2 Installation on Microsoft Windows operating systems . . . 5

5 Running **5**

6 Scenario generation **5**
6.1 Boundless Simulation Area Model ("Boundless") . . . 6
6.2 Chain Model ("ChainScenario") . . . 7
6.3 Column Mobility Model ("Column") . . . 8
6.4 Disaster Area model ("DisasterArea") . . . 8
6.5 Gauss-Markov models . . . 8
6.5.1 The original Gauss-Markov model ("OriginalGaussMarkov") . . . 8
6.5.2 The Gauss-Markov model ("GaussMarkov") . . . 8
6.6 The Manhattan Grid model ("ManhattanGrid") . . . 9
6.7 Map-based Models . . . 9
6.7.1 General Usage . . . 9
6.7.2 Installation of Open Source Routing Machine . . . 10
6.7.3 Random Street ("RandomStreet") . . . 11
6.7.4 Map-based Self-similar Least Action Walk ("MSLAW") . . . 12
6.8 Nomadic Community Mobility Model ("Nomadic") . . . 12
6.9 Probabilistic Random Walk Model ("ProbRandomWalk") . . . 12
6.10 Pursue Mobility Model ("Pursue") . . . 12
6.11 Random Direction Model ("RandomDirection") . . . 13
6.12 Random Walk Model ("RandomWalk") . . . 13
6.13 The Random Waypoint model ("RandomWaypoint") . . . 13
6.14 The Reference Point Group Mobility model ("RPGM") . . . 14
6.15 Self-similar Least Action Walk ("SLAW") . . . 14
6.16 SMOOTH Model ("SMOOTH") . . . 14
6.17 Static scenarios ("Static") . . . 15
6.18 Static scenarios with drift ("StaticDrift") . . . 15
6.19 Steady-State Random Waypoint Model ("SteadyStateRandomWaypoint") . . . 15
6.20 Small World In Motion ("SWIM") . . . 16
6.21 Tactical Indoor Mobility Model ("TIMM") . . . 16
6.22 Truncated Lévy Walk ("TLW") . . . 17

7 Converting scenarios to other formats **17**
7.1 CSVFile . . . 18
7.2 Cut . . . 18
7.3 Glomosim / Qualnet . . . 18

7.4 IntervalFormat . . . 18
7.5 ns-2 . . . 19
7.6 ns-3 . . . 20
7.7 The ONE . . . 20
7.8 WiseML . . . 20
7.9 XML . . . 21

8 Importing other formats **21**
8.1 GPXImport . . . 21

9 Scenario analysis **22**
9.1 The Dwelltime application . . . 22
9.2 InRangePrinter . . . 22
9.3 The LinkDump application . . . 23
9.4 The Statistics application . . . 24

10 Scenario visualisation **25**

11 Validation **25**
11.1 Setup . . . 25
11.2 Usage . . . 25
11.3 Determination . . . 26
11.4 Validation . . . 27
11.5 Determination of a BonnMotion app . . . 27
11.6 Validation of a BonnMotion app . . . 28

12 Acknowledgments **28**

References **29**

1 Legal notice

This program is free software; you can redistribute it and/or modify it under the terms of the GNU General Public License as published by the Free Software Foundation; either version 2 of the License, or (at your option) any later version.

This program is distributed in the hope that it will be useful, but WITHOUT ANY WARRANTY; without even the implied warranty of MERCHANTABILITY or FITNESS FOR A PARTICULAR PURPOSE. See the GNU General Public License for more details.

You should have received a copy of the GNU General Public License along with this program; if not, write to the Free Software Foundation, Inc., 59 Temple Place, Suite 330, Boston, MA 02111-1307 USA

2 Contact information

eMail:
info@bonnmotion.net
URL:
`http://bonnmotion.net/`
Postal address:
Nils Aschenbruck
Institute of Computer Science, University of Osnabrück
Albrechtstraße 28, 49076 Osnabrück, Germany

3 Introduction

BonnMotion is a Java software which creates and analyzes mobility scenarios and is most commonly used as a tool for the investigation of mobile ad hoc network characteristics. The scenarios can also be exported for several network simulators, such as ns-2, ns-3, GloMoSim/QualNet, COOJA, MiXiM, and ONE. BonnMotion is being jointly developed by the Communication Systems group at the University of Bonn, Germany, the Toilers group at the Colorado School of Mines, Golden, CO, USA, and the Distributed Systems group at the University of Osnabrück, Germany.

4 Installation

To use this software, you need to have a JDK or JRE installed. It has been compiled and tested with Java `v1.8.0_25`.

During the installation, a few other shell scripts / batch files are created in the `bin` folder, which you can move and rename as you like:

- `bm` is a wrapper that starts the BonnMotion application. Starting it without command line parameters prints a detailed help message.
- `compile` compiles the sources. By default, only applies to files that were changed after the last compilation. To re-compile all sources, use: `compile all`.

- `makedoc` uses javadoc to create a source code documentation.

Since this distribution does not include pre-compiled class files, the `compile` script needs to be executed before the first usage of BonnMotion.

4.1 Installation on UNIX operating systems

If you are using a UNIX platform, simply unpack the archive and run the `install` script. You will then be asked for the location of your Java binary path, i.e. the directory containing the Java interpreter and possibly the Java compiler and the javadoc utility.

NOTE: We have not yet run our shell scripts on other platforms than Linux or Cygwin. We would be happy if you informed us about changes necessary to run them under other operating systems so we can incorporate them into our distribution.

4.2 Installation on Microsoft Windows operating systems

NOTE: The batch files for MS Windows have not yet been thoroughly tested.

If you are using Microsoft Windows, please edit the variables `JAVAPATH` and `BONNMOTION` in the `install.bat` and then execute it.

5 Running

All applications described below are started via the `bm` wrapper script. The syntax is as follows:

```
$ ./bm <parameters> <application> <application parameters>
```

The application can be a mobility model or e.g. the Statistics application used to analyze scenario characteristics.

Starting the script without command line parameters prints further help. Usage examples are given in the sections below.

6 Scenario generation

Currently, there are several mobility models available, which are introduced later on with some comments on implementation details. For further details on the models themselves, please refer to the literature. Various synthetic models were proposed during the last decade. There have been several general surveys [9, 6, 4, 22] as well as some specific ones for vehicular models [13].

There are two possibilities to feed input parameters into the scenario generation: The first is to enter the parameters on the command line, and the second is to have a file containing the parameters. These two methods can also be combined; in this case, the command line parameters override those given in the input file.

The scenario generator writes all parameters used to create a certain scenario to a file. In this way, settings are saved and particular scenario parameters can be varied without the need to re-enter all other parameters.

Important parameters used with all models are the following: The node number is set with `-n`, the scenario duration (in seconds) with `-d` and the `-i` parameter specifies how many

additional seconds at the beginning of the scenario should be skipped. With `-x` and `-y`, the width and height (in meters) of the simulation area are set. Some models (e.g., Random Waypoint) support 3D movements. Specifying `-z` sets the depth (in meters) of the simulation area and results in 3D movement generation. In this case, the output also defaults to 3D. Otherwise, it defaults to 2D. However, note that the output dimension can be controlled independently by providing `-J <2D|3D>` which will overwrite the output dimension according to the argument provided in any case. With `-R`, the random seed can be set manually.

Cutting off the initial phase is an important feature and therefore, `-i` has a high default value: It has been observed that with the Random Waypoint model, nodes have a higher probability of being near the center of the simulation area, while they are initially uniformly distributed over the simulation area. In our implementation of the Manhattan Grid model, all nodes start at (0,0) for simplicity.

Usage examples:

```
$ ./bm -f scenario1 RandomWaypoint -n 100 -d 900 -i 3600
```

This creates a Random Waypoint scenario with 100 nodes and a duration of 900 seconds. An initial phase of 3600 seconds is cut off.

A scenario is saved in two files: The first, with the suffix `.params`, contains the complete set of parameters used for the simulation. The second, with the suffix `.movements.gz` contains the (gzipped) movement data.

Now, you can take a look at the `scenario1.params` file to see all parameters used for the simulation, and if you wanted to create a similar scenario, only with a higher mobility, you can do this with

```
$ ./bm -f scenario2 -I scenario1.params RandomWaypoint -h 5.0
```

which takes the whole parameter set from scenario1.params and overrides the maximum speed with 5 m/s.

6.1 Boundless Simulation Area Model ("Boundless")

Creates scenarios according to [9]. A model that converts a 2D rectangular simulation area into a torus-shaped simulation area - where no boundaries exist. In addition, this model's nodes have some special relationships between previous direction of travel and velocity with its current direction of travel and velocity. In this model, both the velocity vector and position are updated every Δt time steps according to the following formulas:

$$v(t + \Delta t) = min[max(v(t) + \Delta v, 0), V_{max}];$$

$$\theta(t + \Delta t) = \theta(t) + \Delta\theta;$$

$$x(t + \Delta t) = x(t) + v(t) * cos\theta(t);$$

$$y(t + \Delta t) = y(t) + v(t) * sin\theta(t);$$

where V_{max} is the maximum velocity defined in the simulation, Δv is the change in velocity which is uniformly distributed between $[-A_{max} * \Delta t, A_{max} * \Delta t]$, A_{max} is the maximum acceleration of a given MN, $\Delta\theta$ is the change in direction which is uniformly distributed

between $[-\alpha * \Delta t, \alpha * \Delta t]$, and α is the maximum angular change in the direction an MN is traveling.

6.2 Chain Model ("ChainScenario")

The Chain model is not a model itself but a concatenation of implemented models described in this document. In some cases it is necessary to model scenarios in which mobile nodes behave in different ways depending on time and position. With the Chain model, the mobile nodes' final position of the N-1-th scenario is linked to the initial position of the N-th scenario.

The Chain model works in the following way: it permits to specify a few known models (e.g., Random Waypoint, Manhattan, RPGM, etc.), each one with its own set of parameters as they are defined in this documentation. Therefore, it is possible to define the behaviour of each model separately. Each model that is part of the Chain must be enclosed by quotation marks ("") in order to be distinguished from the rest.

In addition to the individual models, Chain model accepts two parameters: `-m <mode>` specifies if the initial positions of the N-th scenario have a delay of 2 seconds (mode = 1) or not (mode = 0, default). The optional parameter `-P` enables the generation of a set of files for each individual scenario, in addition to the whole chain scenario.

There are two parameters which must be coordinated along all individual models. The number of mobile nodes must be the same for all scenarios, otherwise chain scenario generation will fail. The simulation area may differ between scenarios, but if the final positions of the N-1-th scenario are out of the scope of the N-th scenario's simulation area, generation will also fail.

The following example generates a chain scenario named chainTest and concatenates a RandomWaypoint and a ManhattanGrid scenario. The complete chain scenario will have a duration of 400 seconds (200 sec. for each model), and 10 mobile nodes (remember that this number must be the same for all models). Since the `-m` parameter is defined as 1, a delay of 2 seconds between the two scenarios will be introduced:

```
$ ./bm -f chainTest ChainScenario -m 1 -P "RandomWaypoint -d 200 -n 10 -x 500 -y
    500 -o 3" "ManhattanGrid -d 200 -n 10 -x 500 -y 500"
```

An example of when chain model could help to model a scenario from reality, consider a city with a campus (could be a factory, university, etc.). It could be interesting to model mobility inside the campus, and after some time, to see what happens when students move from campus to their homes.

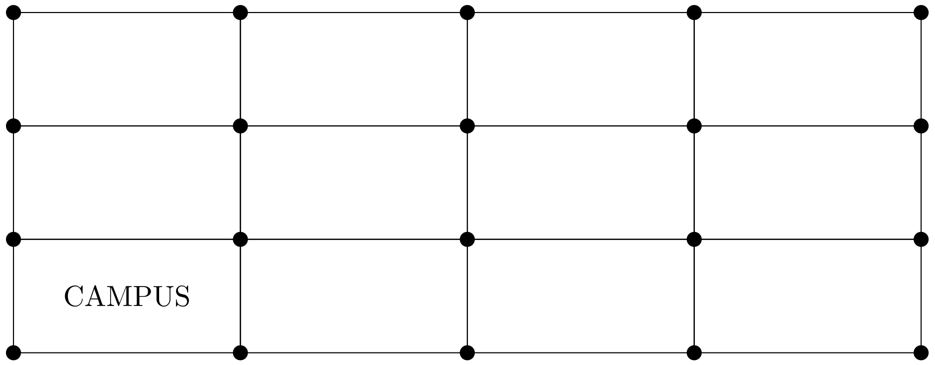

The following line generates a chain scenario for this:

```
$ ./bm -f campus ChainScenario -m 1 "RandomWaypoint -d 200 -n 10 -x 100 -y 100 -o
    3" "ManhattanGrid -d 200 -n 10 -x 400 -y 300 -u 4 -v 3"
```

6.3 Column Mobility Model ("Column")

Creates scenarios according to [9]. A group mobility model where the set of MNs form a line and are uniformly moving forward in a particular direction. In this model the user will specify the number of node and number of groups. The number of nodes must be evenly divisible by the number of groups, i.e., all node group must be completely filled - no lone nodes. The column of reference points picks a random orientation angle and random movement vector. The nodes follow their individual reference point across the map. They have a parameter, `-s <maxDist>`, that determines how far away their random movements around their reference point may be - exactly like the Nomadic model.

6.4 Disaster Area model ("DisasterArea")

Creates scenarios according to [2].

- Tactical areas can be defined with the `-b` parameter following a list of coordinates for the area (seperated by "`,`"). The last three values in this list are type, wanted groups and transportgroups.
- Type can be 0 for incident location, 1 for patients waiting for treatment area, 2 for casualties clearing station, 3 for technical operational command and 4 for ambulance parking point.
- For every specified incident location there must be a patients waiting for treatment area specified, for every patients waiting for treatment area a casualties clearing station and for every ambulance parking point a casualties clearing station.

6.5 Gauss-Markov models

6.5.1 The original Gauss-Markov model ("OriginalGaussMarkov")

This implementation of the Gauss-Markov model follows the publication [17]. In this implementation, the mean velocity vector mu is not specified directly; instead, the norm is specified using `-a` and a random vector with this norm is assigned to each station. Of course, a norm of 0 yields only the vector (0,0). The implementation also allows the user to specify a maximum speed. A velocity vectors with a larger norm will be multiplied with an appropriate scalar to reduce the speed to the maximum speed.

The model has been adapted to deal with scenario borders in the following way: If a station moves onto the border, its velocity vector as well as its expected velocity vector are "mirrored".

6.5.2 The Gauss-Markov model ("GaussMarkov")

This is the implementation of the Gauss-Markov model, which rather follows the description in [9], though it is not the same. The main commonalites are that for each mobile node, two seperate values are maintained instead of one speed vector: The mobile's speed and

its direction of movement. Also the default method of handling mobile nodes that move out of the simulation area is closely related to [9]: Nodes may continue to walk beyond the area boundary, which causes the next movement vector update not to be based on the prior angle, but on an angle that brings the nodes back onto the field. Therefore, the field size is automatically adapted to the node movements after scenario generation.

The main difference to [9] is that new speed and direction of movement are simply chosen from a normal distribution with a mean of the respective old value (the standard deviation is specified on the command line using `-a` and `-s`). Speed values are constrained to a certain interval that can be specified on the command line using `-m` and `-h`: If a newly chosen speed value is outside of this interval, it is changed to the closest value inside of the interval (which is either the minimum or the maximum value).

The behaviour described above can be modified with several command line switches: Using `-b`, the size of the simulation area is fixed and nodes simply "bounce" at the area boundaries. Using `-u`, the speed values outside of the valid speed interval are adapted in a way that leads to a uniform distribution of node speeds (instead of peaks around the interval boundaries).

6.6 The Manhattan Grid model ("ManhattanGrid")

The Manhattan Grid model is introduced in [10]. In this model, nodes move only on predefined paths. The arguments `-u` and `-v` set the number of blocks between the paths. As an example, `-u 3 -v 2` places the following paths on the simulation area:

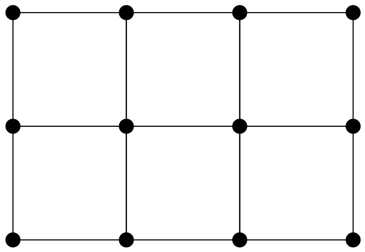

Our implementation contains some (reasonable) modifications of the Manhattan Grid model:

1) An additional parameter we introduce is the minimum speed of a mobile node. This is helpful because the speed of a mobile can be arbitrarily close to 0 and since the model defines that the speed is to be updated in _distance_ intervals, there can be very long periods of very slow node movement without this parameter.

2) The possibility to have nodes pause was added with help of two additional parameters: The pause probability (if a node does not change its speed, it will pause with that probability) and the maximum pause time.

Note that it is especially important to cut off an initial phase with this model, because in our implementation, all nodes start at the same position (0,0).

6.7 Map-based Models

6.7.1 General Usage

Map-based mobility models in BonnMotion require **(i)** an OpenStreetMap (OSM) map file (in `pbf` format[1]) and **(ii)** a URL to a server running an instance of Open Source Routing

[1] `http://wiki.openstreetmap.org/wiki/PBF_Format`

Machine (OSRM)[2], dedicated to computing optimal routes on the road network. OSM map extracts can be downloaded from, e.g., Geofabrik[3]. For validation purposes, we set up an OSRM (v4.8.1) instance with a small map extract. However, in order to start productively using a map-based model, you need to set up an instance of OSRM yourself. Instructions on how to accomplish this can be found in Section 6.7.2. Note that bigger map extracts result in bigger graphs and require a fair amount of computing resources. Smaller-sized map extracts should, however, pose no problem for user-grade computers.

As described in [29], the way geographic restrictions are modeled in BonnMotion raises the question of how to cope with routes which cross the border of the bounding box (simulation area). The -C parameter controls the clipping method used in these cases. Currently, there are three options (cf. [29]):

CLIP_RESIZE_BB: Do not clip routes but resize the bounding box instead after the mobility generation has finished to include all routes ('-C 0').

CLIP_USE_NEW_LENGTH: Clip routes and use the length of the (new) segments connecting the clipping points ('-C 1').

CLIP_USE_OLD_LENGTH: Clip routes and use the total length of the (old) route part outside the bounding box ('-C 2').

Another distinctive feature of map-based models in BonnMotion is the generation of a .movements.geo.gz file in addition to the .movements.gz file. This output file includes all waypoints as lon/lat pairs in decimal degrees (see [29] for the reasoning behind this). All Euclidean (x, y)-coordinates in the .movements.gz file are projections of the lon/lat-coordinates using the Web Mercator projection (EPSG:3857). Furthermore, a .road_network.dat file is generated, containing data about the road network graph defined by the input OSM map extract and the bounding box.

All map-based models share the following parameters:

```
-B <left> <bottom> <right> <top>   : GPS coordinates (lon/lat in decimal degrees)
    of scenario bounding box. Must be inside the OSM map extract.
[-C <clipping method>]             : A number {0,1,2} indicating the clipping
    method to use for routes crossing the border of the bounding box. Default is 0
    for CLIP_RESIZE_BB.
[-m <route service metric string>] : A string ("Pedestrian" or "Car") defining the
    metric used by the routing service for computing optimal routes. Currently has
    no effect since the OSRM URL already defines the metric used by that instance.
    Default is "Pedestrian".
-o <OSM (.pbf) input file>         : OSM map extract defining the road network.
    Must be identical to or a subset of the input map for OSRM.
-u <route service URL>             : URL to the OSRM routing service instance.
```

6.7.2 Installation of Open Source Routing Machine

As mentioned in Section 6.7.1, it is necessary to set up OSRM. All map-based models have been tested with OSRM v4.8.1, so we recommend to use this version. However, newer versions might also work. **NOTE**: For validation purposes **only**, we have already set up an OSRM server and you only need to install your own instance if you want to start productively using a map-based model. The necessary steps for installing your own OSRM instance are as follows:

[2] http://project-osrm.org/
[3] http://download.geofabrik.de/

1. Get OSRM v4.8.1

```
$ wget https://github.com/Project-OSRM/osrm-backend/archive/v4.8.1.tar.gz
$ tar xvfz v4.8.1.tar.gz
$ cd osrm-backend-4.8.1
```

2. Copy source code changes (for minimal JSON responses)

```
$ cp -a $BM/doc/OSRM/osrm-backend-4.8.1/descriptors/* descriptors/
```

3. Compile[4]

```
$ mkdir -p build; cd build; cmake ..; make
```

4. Create symlink[5]

```
$ ln -s ../profiles/lib lib
```

5. Copy lua profile files and scripts to build-dir[6]

```
$ cp -a $BM/doc/OSRM/osrm-backend-4.8.1/build/* .
```

6. Prepare OpenStreetMap PBF file (e.g., extract from `planet.osm.pbf`)

```
$ ./prepare_pbf.sh maps/map_extract
```

7. Run OSRM server instance

```
$ ./start_routed.sh maps/map_extract.osrm 5001 12
```

8. Use same (or subset of) map extract for your map-based mobility model

6.7.3 Random Street ("RandomStreet")

Creates scenarios according to [3]. Note that in BonnMotion versions prior to 3.0.0, an additional input parameter file was necessary. Since the re-write of map-related code for version 3.x, all input parameters can be set directly on the command line.

```
RandomStreet parameters:
   -p <maxPause>            : Maximum pause time in seconds.
   -s <minSpeed> <maxSpeed> : Minimum and maximum speed in m/s.
```

[4] `https://github.com/Project-OSRM/osrm-backend/wiki/Building%20OSRM`
[5] `https://github.com/Project-OSRM/osrm-backend/wiki/Running-OSRM`
[6] `https://github.com/sosm/cbf-routing-profiles/`

6.7.4 Map-based Self-similar Least Action Walk ("MSLAW")

Creates scenarios according to [28]. Note that in BonnMotion versions prior to 3.0.0, an additional input parameter file was necessary. Since the re-write of map-related code for version 3.x, all input parameters can be set directly on the command line.

```
MSLAW parameters:
   -w <Number of waypoints> : Number of waypoints to generate during initialization.
   -p <Minimum pause time>  : Minimum pause time in seconds.
   -P <Maximum pause time>  : Maximum pause time in seconds.
   -b <Levy exponent>       : Levy exponent for pause time.
   -h <Hurst parameter>     : Hurst parameter for self-similarity of waypoints.
   -l <distance weight>     : Distance weight used by LATP algorithm.
   -r <clustering range>    : Clustering range in meters.
   -Q <Cluster ratio>       : Cluster ratio as divisor (ratio is then 1/divisor).
   -W <waypoint ratio>      : Waypoint ratio as divisor (ratio is then 1/divisor).
   -s <minSpeed> <maxSpeed> : Minimum and maximum speed in m/s.
   -D <distance metric>     : Metric to use for distance calculations. One of
       ["Route"|"Flight"] (default is "Route").
```

6.8 Nomadic Community Mobility Model ("Nomadic")

Creates scenarios according to [9]. A group mobility model where a set of MNs move together from one location to another. An example scenario this model simulates would be a guided tour of a city or museum. The tour guide and tourists move from spot to spot and they would all roam around each particular location individually.

Each group of mobile nodes has an invisible reference node that they follow around the simulation. Once the reference point changes, all of the mobile nodes travel to the new location and begin roaming. Their roaming is defined by picking random locations within some predefined roaming radius of the reference point. This maximum roaming distance is defined by the `-r` flag.

6.9 Probabilistic Random Walk Model ("ProbRandomWalk")

Creates scenarios described in [9]. A model that utilizes a set of probabilities to determine the next position of an MN. The model utilizes a probability matrix that defines the probabilities of a node moving forwards, backwards, or remaining still in both the x and y direction. Once the direction of travel has been determined, the node will travel with a fixed speed (as per the Toilers' code) for a specified allotment of time. This amount of time is set with the `-t` flag. The desired time should follow the flag after a space, e.g., `-t 15`, for 15 seconds.

6.10 Pursue Mobility Model ("Pursue")

Creates scenarios according to [9]. This model attempts to represent nodes tracking a single targeted node. This model could represent police forces chasing down a criminal on the run. The model uses Random Waypoint with no pauses to move the pursued target. The model uses one equation to update the position of each pursuing node:

$$new\_position = old\_position + acceleration(target - old\_position) + random\_vector$$

Where $acceleration(target - old\_position)$ is information on the movement of the node being pursued and $random\_vector$ is a random offset for each node. The $random\_vector$ is

a vector in a random direction with a configurable magnitude by using the `-m` flag. You will want to keep this magnitude low (0 - 10) to ensure the pursuing nodes maintain effective tracking of the target.

6.11 Random Direction Model ("RandomDirection")

Creates scenarios described in [9]. A model that forces MNs to travel to the edge of the simulation area before changing direction and speed. This model does not suffer from the density waves in the center of the simulation space that Random Waypoint model does. In this model, MNs choose a random direction in which to travel similar to the Random Walk Mobility Model. An MN then travels to the border of the simulation area in that direction. Once the simulation boundary is reached, the MN pauses for a specified time, chooses another angular direction (between 0 and 180 degrees) and continues the process.

6.12 Random Walk Model ("RandomWalk")

Creates scenarios described in [9]. A simple mobility model based on random directions and speeds. In this mobility model, an MN moves from its current location to a new location by randomly choosing a direction and speed in which to travel. The new speed and direction are both chosen from predefined ranges, $[speedmin, speedmax]$ and $[0, 2\pi]$ respectively. If an MN which moves according to this model reaches a simulation boundary, it "bounces" off the simulation border with an angle determined by the incoming direction. The MN then continues along this new path. This model can be configured such that the nodes continue along their path for a set amount of time or a set distance. This can be set by the `-t` flag for time limited mode, or by the `-s` for distance limited mode. The desired time or length should follow the flag after a space e.g., `-t 10`.

6.13 The Random Waypoint model ("RandomWaypoint")

If you provide a depth, i.e., the `-z` parameter is provided, BonnMotion assumes you want to create a 3D scenario. Our implementation includes a feature to restrict the mobiles' movements: With `-d 1`, nodes move only along the x-axis, with `-d 2`, nodes move either along the x- or along the y-axis (with a probability of 0.5 each) and with the default `-d 3`, it is the classical 2D Random Waypoint model as you know it. For 3D scenarios, there are two more options: With `-d 4`, nodes move either on x or y or z and with `-d 5` you get the classical 3D Random Waypoint behavior.

Another feature is the `-c` switch that causes positions to be chosen from a circle that fits into the simulation area rather than from the simulation area itself.

Instead of choosing new destinations uniformly distributed from the simulation area, "attraction points" can be defined with the `-a` parameter, followed by the data characterizing the attraction points. Each attraction point is defined by five floating point numbers, in this order: $< coord_x >, < coord_y >, < intensity >, < stddev_x >, < stddev_y >$.

- The coordinates $< coord_x >, < coord_y >$ give the attraction point's position.
- The intensity levels weight the attraction points: A point with an intensity x times as high as another point's will also attract a node with a probability which is x times as high.

- The last two parameters are the standard deviations of the Gaussian distribution with mean 0 that is used to determine the nodes' distances to the attraction point on dimension x and y, respectively.

The parameters for several attraction points are simply concatenated, seperated by commas. For example, to place two attraction points on the simulation area, the first at (100,100) with intensity 1 and both standard deviations 20, the second at (350,200) with intensity 1.5 and standard deviations 31 and 35, use: `-a 100,100,1,20,20,350,200,1.5,31,35`.

6.14 The Reference Point Group Mobility model ("RPGM")

The implementation of this model is based on the design given in [12]. Since Hong *et al.* did not specify a concrete pausing behavior, we based our implementation loosely on [9]: Whenever the fictional group leader is pausing, all the member nodes of the respective group are pausing for the same amount of time, as well.

The model does also include the possibility to have "dynamic" groups: When a node comes into the area of another group, it changes to this new group with a probability that can be set with `-c <probability>`. Deactivate this feature with `-c 0`. Note that when this feature is activated, "empty" groups may be moving along the simulation area and nodes coming into their areas may change their memberships to these.

6.15 Self-similar Least Action Walk ("SLAW")

Creates scenarios according to [16]. The implementation is loosely based on the Matlab code provided by the authors [25].

6.16 SMOOTH Model ("SMOOTH")

Creates scenarios described in [21]. SMOOTH presents a mobility model that is both realistic and simple by creating traces that match human movements in a manner that is simple to use. The movements that are generated by SMOOTH follow power-law distribution, and the movements of the mobile nodes are generated by a predetermined probability. As SMOOTH takes into account the fact that node movements can be predicted to some extent, the movements generated are also influenced by their previous movements.

SMOOTH initially places each node near a cluster, with more nodes being placed near clusters with a higher popularity. These clusters are distributed evenly about the simulation area, and far enough apart so that no cluster is in the transmission range of another. When a node changes location, the node transitions to either a new location, or a previously-visited location, based on predetermined probabilities. These movements, as well as the time that a node pauses at a location, are power-law distributed. The implementation is based on C code provided by [30].

```
Parameters for SMOOTH:
   -g <range>          : Transmission range of mobile nodes.
   -h <clusters>       : Total number of clusters in the network.
   -k <alpha>          : Alpha value for the flight distribution.
   -l <f_min>          : Minimum value for the flight distribution.
   -m <f_max>          : Maximum value for the flight distribution.
   -o <beta>           : Beta value for the pause-time distribution.
   -p <p_min>          : Minimum value for the pause-time distribution.
   -q <p_max>          : Maximum value for the pause-time distribution.
```

```
[-s <print>]          : (optional) Specifies what variable is to be printed to
                        the command line. All options will be printed to output
                        file in all cases, or if the option is not specified.
                        Valid options are: 'CN', 'CT', 'ICT' or 'ALL'.
[-t <max_locations>] : (optional) Specifies the maximum number of locations for
                        the scenario (default: 20000).
```

6.17 Static scenarios ("Static")

By default, nodes in static scenarios are homogeneously distributed over the simulation area. There are two possibilities for non-homogeneous node distributions:

- Attraction points can be defined with the `-a` parameter; a detailed explanation of this feature is given in the "Random Waypoint" section.
- With the `-l` parameter, the simulation area can be divided into several areas with different node densities along its x-axis. Given the number n of density leves, each of the n areas will contain a fraction of approximately $2 * k/(n * (n + 1)), 1 \leq k \leq n$, of the nodes. (The density decreases from left to right.)

The following example shall illustrate this: Distributing 150 nodes on a 300m x 300m field with 3 density levels, approx. 75 nodes will lie within the rectangle area with the corners (0,0) and (100,300), approx. 50 nodes will lie within (100,0) and (200,300) and approx. 25 nodes will lie within (200,0) and (300,300).

6.18 Static scenarios with drift ("StaticDrift")

In this model the user has to provide an input file with the initial positions of all nodes. The positions are drifted along ajustable intervals and saved as a BonnMotion scenario. The format of the input file has to be:

```
x1 y1 z1
x2 y2 z2
...
```

Each row of the file belongs to the position of one node.

Δx and Δy are defined with the `-X` and `-Y` parameters . If Δx and Δy should be equal the value can be set with the parameter `-B`. The final position of one node n is $[x_n - \Delta x, x_n + \Delta x], [y_n - \Delta y, y_n + \Delta y]$. There is no movement in this model.

6.19 Steady-State Random Waypoint Model ("SteadyStateRandomWaypoint")

The Steady-State Random Waypoint Mobility Model [23, 24] uses the random waypoint model, where a node picks a random point on the simulation area and a random speed and then travels to that point at the chosen speed. Once it arrives, the node pauses for a randomly chosen pause time, and then repeats the process until the simulation ends. The Steady-State Random Waypoint Mobility Model picks initial node positions, speeds, and pause times according to the steady-state distributions of the random waypoint model, so that no time is required to let the distributions of position and speed settle at the beginning of the simulation.

```
Parameters for SteadyStateRandomWaypoint:
   -o <speed mean>  : Specifies mean speed (must be > 0).
   -p <speed delta> : Specifies speed delta (must be >= 0).
   -k <pause mean>  : Specifies mean pause time (must be >= 0).
   -l <pause delta> : Specifies pause delta (must be >= 0).
```

NOTES: The minimum speed must be positive, because the steady-state distribution is degenerate when the minimum speed is 0. An end time of zero only gives the initial configuration of the nodes without any movement.

This implementation is based on mobgen written by Jeff Boleng <jeff@boleng.com> (Ph.D. 2002 from the Colorado School of Mines) and mobgen-ss by Nick Bauer (M.S. 2004 from the Colorado School of Mines).

6.20 Small World In Motion ("SWIM")

Creates scenarios according to [18]. The implementation is based on the C++ code provided by the authors [19].

6.21 Tactical Indoor Mobility Model ("TIMM")

Creates scenarios according to [1]. Tactical Indoor Mobility Model (TIMM) models the node movement according to a small ruleset (cf. Figure 1) with an underling graph representing the building.

`Building graph`

The building graph file contains the graph representation of the underling building/scenario. Lines starting with `#` are ignored, starting with `Pos=` are interpreted as vertexes. The syntax is:

```
Pos=<NodeName>,x,y,NeighborNode1;NeighborNode2;...;NeighborNodeN,<Type>
```

Valid types are `ROOM`, `DOOR`, `STRUCT`, and `DISTANCE`. The model does not distinguish between `ROOM`, `STRUCT`, and `DISTANCE`. However, the intention is to use `ROOM` for vertexes representing rooms and `STRUCT` for vertexes, which are required to model the building structure. `DISTANCE` vertexes should not be inserted into the building graph file. These are created by the model if necessary, due to the configured maximal distance between vertexes in the graph. Exactly one vertex must be labeled as `StartVertex`. An example building graph file is located in the `doc/TIMM_example` folder.

`Door opening time`

This parameter is used to determine how long it takes to secure a room. This includes opening the door, looking in the room, and taking necessary steps.

Keep in mind, that movements in tactical and non-tactical scenarios are completely different! An example parameter file is located in the `doc/TIMM_example` folder.

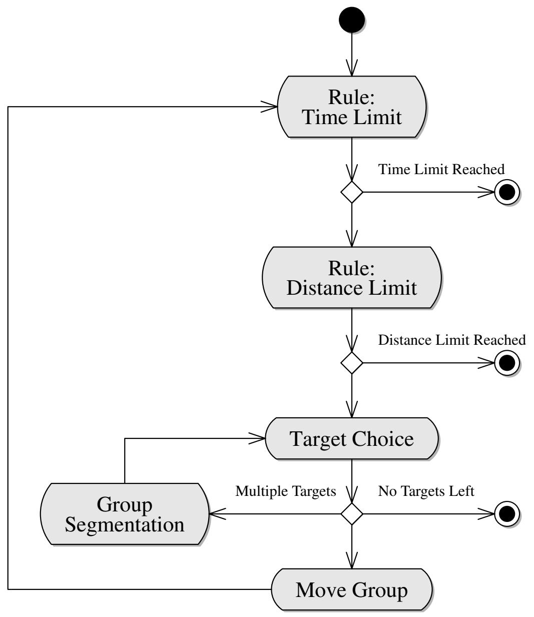

Figure 1: Schematic view of the TIMM ruleset

6.22 Truncated Lévy Walk ("TLW")

Creates scenarios according to [27]. The implementation is based on the MATLAB code written by Kyunghan Lee (KAIST) [25]. The TLW model uses a specific speed model combined with a simple random walk model in which a walker follows a sequence of *steps*. A step contains the direction, length and duration of a flight followed by a pause time. At the beginning of each step, a walker chooses a random direction within $[0°, 360°]$, a finite random flight time and values from a bundle of randomly generated flight lengths and pause times, which are Lévy distributions with coefficients *falpha* and *palpha*. The flight length coefficient *falpha* and the pause time coefficient *palpha* have to be in $[0.1, 2]$. With the parameter `-c`, you can set the flight length scale factor (the pause time scale factor is always set to 1). With the `-t` and `-p` parameters, you can change the truncation points for the flight lengths and the pause times.

```
Parameters for TLW:
   -a <falpha>   :  flight length alpha
   -b <palpha>   :  pause time alpha
   -c <scale>    :  flight length scale factor
   -t <f_trunc>  :  flight length truncation (meters)
   -p <p_trunc>  :  pause time truncation (seconds)
```

7 Converting scenarios to other formats

The native format in which BonnMotion saves the movement traces is node-by-line waypoint based. This means that there is one line for each node. This line contains all the waypoints. A waypoint is a position at which the movement of a node (e.g. direction, velocity) changes. A waypoint consists of:

- the simulation time in seconds at which the waypoint is reached by the node
- the x and y coordinates of the position of the waypoint.

MiXiM (`http://mixim.sourceforge.net/`) is a simulation framework for wireless and mobile networks, which can handle the BonnMotion trace format without converting.

The Simulation platform COOJA (`http://www.contiki-os.org/`) can be used with BonnMotion traces, using the WiseML format (see: section 7.8).

7.1 CSVFile

When processing a generated mobility scenario with third-party tools, a general CSV-formatted file might be more useful than the native Bonnmotion format. The "CSVFile" app converts a mobility scenario to a comma-separated value file (suffix `.csv`), where each line contains the tuple (i, t, x, y), where i is the node ID, t the simulation time, and (x, y) the position of the waypoint. If the scenario is 3D, there is also a 5th value (z-coordinate).

```
Parameters for CSVFile:
   -f <filename>     : Specifies scenario name.
   [-d <delimiter>]  : (optional) Specifies delimiter (default: " ").
   [-h]              : (optional) Print header (default: no header).
```

7.2 Cut

The "Cut" application reads an existing scenario and extracts the movement within a time interval given by start (`-b`) and end time (`-e`) parameters. This extract is written to a new scenario with corresponding `.movements.gz` and `.params` files. Note that the scenario names are base names and may not contain any file suffixes.

```
Parameters for Cut:
   -f <filename>     : Specifies scenario name (input).
   -d <filename>     : Specifies name of new scenario (output).
   -b <starttime>    : Start time of interval.
   -e <endtime>      : End time of interval.
```

7.3 Glomosim / Qualnet

The "GlomoFile" application creates files with two suffixes. The first is `.glomo_nodes` and the second one is `.glomo_mobility`. They can be used with Glomosim (2.0.3) and Qualnet (3.5.1). Use the `-q` switch for Qualnet: This causes nodes to be numbered starting at 1, not at 0.

7.4 IntervalFormat

The native format implies that during the simulations for each event the current node positions have to be calculated based on the waypoints. If there are many events, this may have a negative impact on the runtime of a simulation. An alternative is to use an interval based approach. The nodes are regarded as stationary for an interval. The positions of the nodes are updated periodically after each interval by a specific position update event. By doing so, the current node positions do not have to be calculated for each event. However, the number events is increased, which may also influence the runtime of a simulation negatively. A factor

that has a major impact in this context is the interval length. Smaller intervals yield higher accuracy but also more events. Overall, it is a trade-off between the number of events and the runtime per event.

Trace files in the BonnMotion's native trace format can be transformed to an interval-based format using the IntervalFormat application. The interval length can be specified using the -l option. The default value is one second. The interval trace format is an interval-by-line based. This means that there is one line for each interval of each node. A line consists of:

- the node number
- the simulation time in seconds (in intervals)
- the x and y coordinates of the position of the node for the interval

The IntervalFormat application prints the waypoints (ordered by node and time) for every interval step.

- The used interval can be specified using the `-l` switch.
- Using the `-s` switch the header can be skipped

7.5 ns-2

The "NSFile" application is used to generate two files that can be integrated into a TCL script to start an ns-2 simulation via the "source" command.

The file with the suffix `.ns_params` sets some variables needed to set up the simulation in an array named `val`: the keys `x` and `y` refer to width and height of the simulation area, `nn` refers to the number of nodes and `duration` to the duration of the simulation.

The file with the suffix `.ns_movements` schedules the movements of the node objects that are expected to be in an array named "`node_`", numbered starting at 0. The simulator object is expected to be in the variable "`ns_`".

As a side note, the "NSFile" application places an additional margin around the simulation area, because ns-2 versions up to 2.34 regularly crash when nodes move at the border of the simulation area. (Actually, this has only been observed with the Manhattan Grid model up to now, but this procedure is generally carried out just to play it safe.)

Usage example:

```
$ ./bm NSFile -f scenario1
```

This creates the two files `scenario1.ns_params` and `scenario1.ns_movements`.

The NSFile command line switches:

```
Parameters for NSFile:
   -f <filename> : BonnMotion scenario to convert.
   [-b <margin>] : (optional) Adds an additional margin around the simulation area,
                   because ns-2 versions up to 2.34 crash when nodes move at the
                   border of the simulation area (default: 10).
   [-d]          : (optional) Disables the usage of module specific converters
                   (e.g. for DisasterArea) and uses the standard converter instead.
```

7.6 ns-3

The "NSFile" (cf. Section 7.5) application can be used to generate movements for ns-3 as it supports ns-2-movementfiles. As ns-3 does not crash if nodes move at the border, the additional margin can be disabled with the parameter `-b`.

An example script for ns-3 is given in `doc/ns3`.

7.7 The ONE

The "TheONEFile" application creates a file with the suffix `.one`, which can be used with the ONE simulator [15]. Use the `-l` parameter to set the sampling interval between two positions of the same node (the default is $1s$).

Usage example:

```
$ ./bm TheONEFile -f scenario1 -l 5
```

This creates the file `scenario1.one` with a sampling interval of $5s$.

7.8 WiseML

WiseML [31] is a description format allowing a standardized storage of traces of experiments. It is based on GraphML and used within the WISEBED Project (`http://www.wisebed.eu/`). Each experiment trace is stored within one file and contains all needed information to identify and reproduce a simulation trace. This BonnMotion application allows the conversion of BonnMotion's native format into WiseML.

The WiseML app has two basic modes of operation. One mode is interval-based, where the positions of all nodes of the scenario are printed at each interval (needs to be provided with the `-L` switch). In contrast, in the contact-based mode the position of all nodes are printed only whenever any node enters or leaves the communication radius of another node. The contact-based mode is activated by providing a transmission range with the `-r` switch. By setting the `-o` switch only positions of nodes, which links change, are printed. If the `-e` switch is used it is also printed which links were disabled or enabled. This information is given by `<enableLink source=nodeid target=nodeid \>` and `<disableLink source=nodeid target=nodeid \>` tags. Note that you can also combine the two modes by using both the `-L` and `-r` switch.

The dimension of the output of the WiseML conversion depends on the presence of a depth in the scenario you want to convert. In other words if the parameter file contains a z-value, the dimension of the output of WiseML will be 3D. Else it will be 2D.

The WiseML converter offers various command line switches to tune the output to the desired format.

```
Parameters for WiseML:
   -f <filename>            : BonnMotion scenario to convert.
   -L <double>              : Interval length between two timestamps
                              (interval-based mode).
   -r <double>              : Transmission range (contact-based mode).
   [-a <altitude>]          : (optional) Default z value (BonnMotion 1.5 does not
                              support 3D traces).
   [-c <compressionlevel>]  : (optional) Compression of the output (0 = NONE,
                              1 = No tabs, 2 = No tabs, no newlines).
   [-F <path to file>]      : (optional) Path to XML footer to include in output.
   [-H <path to file>]      : (optional) Path to XML header to include in output.
```

```
[-I]                    : (optional) Output integer values for the timestamps
                          instead of doubles.
[-N <path to file>]     : (optional) Path to nodeId names. Each row should
                          contain one name.
[-e]                    : (optional) Print which links were enabled/disabled
                          (contact-based mode).
[-o]                    : (optional) Print only positions of nodes, which links
                          are changing (contact-based mode)
```

7.9 XML

The “SPPXml” application is used to generate mobility files in XML format according to the XML schema proposed by Horst Hellbrück as a standardised mobility file format for the research program “Schwerpunktprogramm 1140” (`http://www.tm.uka.de/forschung/SPP1140/`) of the DFG (Deutsche Forschungsgemeinschaft).

SPPXml has an additional parameter `-r` that allows to specify a uniform radio range for all nodes. If this parameter is omitted, a radio range of 250m is used. BonnMotion does not use this radio range in any way. It is however required by the XML schema.

The XML schema corresponding to the XML files generated by “SPPXml” is defined in:
`http://www.i-u.de/schools/hellbrueck/ansim/xml/sppmobtrace.xsd`

The contrib directory furthermore includes a python program `sppmob2bonnmotion.py` that convertw XML mobility traces to the BonnMotion format. It has been tested with Python 2.3.

8 Importing other formats

8.1 GPXImport

GPXImport can be used to import multiple Global Positioning System (GPS) Exchange Format (GPX) files [11] and convert them into one BonnMotion mobility scenario (native format). In default mode, GPXImport only imports the longitude and latitude values of all tracks in all input files. GPX waypoints (note that a GPX does not necessarily have the same semantics as a BonnMotion waypoint) and routes have to be imported explicitly with `-w` and `-r`, respectively. In general, GPX trackpoints, routepoints, and waypoints need to be associated with a timestamp, otherwise they will be ignored.

Tracks or routes *with* `src` tags will be assigned to one node per `src` tag in the mobility scenario. In particular, tracks and/or routes associated with the same `src` tag are assumed to belong to a single node. Tracks or routes *without* `src` tags will be assigned to an extra node, each. Furthermore, all GPX waypoints of one input file are assumed to belong to a single extra node.

Although there are GPS receivers which support a position logging frequency of more than 1Hz, GPX does not support time resolution beyond seconds. Therefore, multiple positions might have the same timestamp associated with them. In this case, the positions are equally distributed within that one second, i.e., n positions with timestamp t are converted to the same positions with times t, $t + \frac{1}{n}$, ..., $t + \frac{n-1}{n}$, respectively.

Since GPS uses World Geodetic System (WGS)84 as its Coordinate Reference System (CRS), the longitude and latitude values first need to be projected to a Cartesian coordinate system (cf. `-p` parameter). In order to make the projection as precise as possible, a best-match projected CRS has to be selected for the GPX input file(s) (see [8, 26]). For example, if

you would like to use Universal Transverse Mercator (UTM) zone 32N (cf., e.g., [20]) as your projected CRS, the parameter value should be "epsg:32632" (European Petroleum Survey Group (EPSG) code 32632).

The command line parameters of GPXImport are as follows (also see `bm -ha GPXImport`):

```
Parameters for GPXImport:
   -f <GPX file> : Input file name(s).
   -p <CRS name> : Name of the projected CRS for coordinate transformation.
   -F <filename> : Output file name (mandatory in case of multiple input files).
   [-c]          : (optional) If set, the created movement file will be compressed.
   [-h]          : (optional) If set, the z-coordinate (altitude/elevation) of the
                   positions is also imported and a 3D scenario is created.
   [-H]          : (optional) Sets default z-coordinate for positions with an
                   invalid/missing value (default: 0).
   [-r]          : (optional) If set, the GPX routes are imported.
   [-w]          : (optional) If set, the GPX waypoints are imported.
```

Usage examples:

```
   $ ./bm GPXImport -f path/to/BoiseFront.gpx -p epsg:32611 -c -w -r
```

This imports all tracks, routes, and waypoints from the GPX file `path/to/BoiseFront.gpx` and transforms the positions using EPSG code 32611 (UTM zone 11N). The output files are `BoiseFront.param` and `BoiseFront.movements.gz` in folder `path/to/`.

```
   $ ./bm GPXImport -f BoiseFront.gpx clementine_loop.gpx -p epsg:32611 -F Test -w -r
```

This imports all tracks, routes, and waypoints from both GPX files and transforms the positions using UTM zone 11N. The output files are `Test.param` and `Test.movements`.

9 Scenario analysis

9.1 The Dwelltime application

The "Dwelltime" application creates statistics on how long nodes stay in a certain area (small square cells) of the simulation area. Based on the cumulative dwell time of the nodes in each cell, a two-dimensional histogram is created (cf. [7]). Let c be a cell and $d_n(c)$ the dwell time of node $n \in N$ in c for the whole simulation time s, then the dwell time $d(c)$ is defined as:

$$d(c) := \frac{\sum_{n \in N} d_n(c)}{|N| \cdot s}$$

Two output files are created by this app: One file with suffix `.bettstetter_statistics` containing one line for each cell with values $(x, y, d(c))$. The second file has the suffix `.bettstetter_statistics2` and contains the same values, but only for cells with $d(c) > 0$.

```
Parameters for Dwelltime:
   -f <filename>    : Specifies scenario name.
   -m <cell length> : Specifies cell length (default: 0.5m).
   -t <time step>   : Specifies discrete time step (default: 0.5s).
```

9.2 InRangePrinter

The InRangePrinter app can be used to get information about the connectivity of nodes in the scenario. It has two modes:

- **Interval mode:** This mode is used to print periodically information about distances to the other nodes. Giving a range changes the output into a binary format indicating nodes in range with 1, out of range nodes with 0.

- **Range mode:** The range mode prints the network topology whenever a node leaves or enter the range of another mode

The app uses a line based format. All lines start with a timestamp followed by per node information in blocks starting and ending with square brackets ("[" and "]"). Each entry in these blocks gives the distance or the indication of in-/out-of-range for a node. The node itself is omitted.

Example for a two node scenario where all nodes are in range at the beginning and leave the range after 1s simulation time:

```
0.0 [ 1 ][ 1 ]
1.0 [ 0 ][ 0 ]
```

Usage examples:

```
$ ./bm -f IRP RandomWaypoint -n 3
$ ./bm InRangePrinter -f IRP -l 1.0
```

9.3 The LinkDump application

The LinkDump application prints information about every single link within a certain time span of a scenario. This information can e.g. be used for a simulator that does not directly model mobility at all. To use the LinkDump application, setting the transmission range using the `-r` parameter is necessary.

Using the `-d` switch, only the link durations are printed. This offers more insight into the distribution of link durations than the averaged value calculated by the Statistics application. In this case, the `-w` switch prevents that wrong durations are printed out due to links that go up before simulation begin or go down after simulation end.

In addition to the link durations, the inter-contact times can be obtained using the `-j` parameter. Additionally you can use the `-t` parameter to show the "start time" for each inter-contact time. Since version 3.x, in compliance with the new map-based model code, the `-g` parameter has been introduced to compute contacts for geo-positions generated by BonnMotion.

```
Parameters for LinkDump:
   -b <begin of time span> : Specifies begin of time span.
   -d                      : Compute link durations (and save to file).
   -e <end of time span>   : Specifies end of time span.
   -f <scenario name>      : Specifies name of the scenario.
   -g                      : Use GPS (geo) coordinates.
   -i                      : Print inter contact times to console.
   -j                      : Compute inter contact times (and save to file).
   -t                      : Print start time of inter-contact times.
   -r <transmission range> : Specifies transmission range.
   -w                      : Print only links that go up and down after begin
                               and before end of time span.
```

9.4 The Statistics application

The "Statistics" application is used to analyze a given scenario. There are two modes of operation: The default is to calculate "overall" statistics (averaged over the simulation time) and the other mode is to calculate "progressive" statistics (values of metrics for certain points in time).

In its default mode, the application creates a file with the suffix `.stats` containing the following information:

- Metrics *independent* of transmission range:
  1. The *relative mobility* is calculated according to [14].
  2. The *average node speed* is the speed averaged over all nodes.
  3. The *average degree of temporal dependence* is a measure for the dependence of a node's velocity on its velocity at some previous time (IMPORTANT framework metric [5]).
- Metrics *dependent* of transmission range (sorted by row, one column per transmission range):
  1. *Average degree of spatial dependence*: How much are the nodes' movements influenced by each other (IMPORTANT framework metric [5])?
  2. *Average node degree*: To how many of the other nodes is one node connected (directly, i.e., over 1 hop)?
  3. *Average number of partitions*: 1 means the network is connected at all times, values larger than 1 indicate that this is not the case.
  4. *Partitioning degree*: How likely is it that two randomly chosen nodes are not within the same connected component at a randomly chosen point in time?
  5. *Average time to link break*: Average time it takes for a link to break off. Only links that go up after the simulation start and go down before the simulation end are taken into account.
  6. *Standard deviation of time to link break* (see above).
  7. *Link breaks*: The total number of links that go up after the simulation start and go down before the simulation end.
  8. *Average link duration*: Similar to time to link break, but in this case all links are counted (IMPORTANT framework metric [5]).
  9. *Total number of links.*
  10. *Average relative speed* (described in [14]) between all nodes in the network.
  11. *Average path availability*: The value of path availability averaged over all node pairs. Path availability is the fraction of time during which a path is available between two nodes i and j (IMPORTANT framework metric [5]).
  12. *Average number of link changes*: The number of link changes averaged over all node pairs. Number of link changes for a pair of nodes i and j is the number of times the link between them transitions from "down" to "up" (IMPORTANT framework metric [5]).

Alternatively to the statistics which are averaged over the whole simulation time, the devolution of certain characteristics can be calculated (progressive mode). See the command line help and the following examples.

Usage examples:

```
$ ./bm Statistics -f scenario1 -r 50,75,100
```

This writes the averaged statistics to `scenario1.stats` for transmission ranges of 50, 75, and 100 meters.

```
$ ./bm Statistics -f scenario1 -r 75 -P
```

This creates a file `scenario1.stats_75.part`, which gives the number of partitions each time it changes.

```
$ ./bm Statistics -f scenario1 -r 50,100 -N -M 10
```

This creates `scenario1.stats_50.nodedeg` and `scenario1.stats_100.nodedeg` that show the devolution of the node degrees. Furthermore, the files `scenario1.stats_50.mincut` and `scenario1.stats_100.mincut` show the minimal cut of the topology every 10 seconds. It is reasonable to specify such a time span for computations that cost much CPU time.

10 Scenario visualisation

"Visplot" is a very simple application that writes those positions to a file where a mobile changes its speed or direction. This file can simply be visualised using e.g. gnuplot.

11 Validation

After a code change it is hard to verify if the change affected the repeatability of past scenarios. For this purpose this script has been developed. It is located in the folder `validate`.

The validate script is a two phase script: In the determination phase the script runs BonnMotion with various parameters and saves hashes and used parameters of the given BonnMotion output in a SQLite database. In the validation phase the script reads the saved parameters and hashes from the database, runs the BonnMotion version to validate with the parameters again, generates hashes of the new output and compares them with the saved hashes.

11.1 Setup

To be able to run the script you have to adjust the values of the BonnMotion paths in the config file: `validate.cfg`.

11.2 Usage

```
$ ./validate.py

    -m, --determine-model [-d, --delete] <filename/folder>
    write hash values into database
        [-d, --delete]    : (optional) delete saved hash values of this model
```

```
                         from the database
    <filename/folder> : filename of modeltest file, or folder with one or
                         more modeltest files

-M, --validate-model [modelname]
validate BonnMotion with hash values saved in database
    [modelname]       : (optional) name of the model to validate if no
                         model provided all saved models will be validated

-a, --determine-app [-d, --delete] <filename/folder>
write hash values into database
    [-d, --delete]    : (optional) delete saved hash values of this app
                         from the database
    <filename/folder> : filename of apptest file, or folder with one or
                         more apptest files

-A, --validate-app [appname]
validate BonnMotion with hash values saved in database
    [appname]         : (optional) name of the app to validate if no
                         app provided all saved apps will be validated
```

11.3 Determination

In the determination phase the script expects a modified BonnMotion parameter file as command line input. In this file parameter values can be defined as constant values, variable steps and sets. Variable steps have the format: {`begin:end:step`}. For instance {`1.0:2.0:0.5`} would result in the parameters 1.0, 1.5 and 2.0. Sets have the format {`element1;element2;...;elementn`}. Example for a model testparameter file:

```
model=RandomWaypoint
ignore=3200.0
randomSeed=1276176155501
x=200.0
y=200.0
duration={100.0:300.0:100.0}
nn={10;100;1000}
circular=false
dim=3
minspeed=1.0
maxspeed=1.5
maxpause=60.0
```

Running the script with this parameter file it would run BonnMotion with these parameter combinations:

duration	nn
100.0	10
200.0	10
300.0	10
100.0	100
100.0	1000

The other parameters would be constant.

If you have to test several sets of parameters which have to be consistent (e.g. number of nodes have to match groupsizes) than you can provide additional parameters with the `[testcase]` keyword. The first parameters in the modeltest file are the default ones. Addi-

tional testcases only have to set the parameters which are different from the default ones.

Example (appended to the parameters above):

```
[testcase]
x=1000.0
y=1000.0
```

This would create a run with all parameter combinations from above and also with a scenario-size of 1000x1000.

The hashes are saved in the specified database in a table named after the model.

Note: The parameter `-d, --delete` erases all saved hashes of the model specified before saving the generated hashes, without further inquiry!

The string {$} is replaced with the current directory path. This can be used to test models which need additional input files (for example RandomStreet)

11.4 Validation

When validating, the script catches parameters and hashes from the database, runs BonnMotion with the parameters, computes hashes from the output and compares the saved hashes with the new ones. If no table name is provided all saved models are validated, otherwise the script expects a table name which exists in the database. After running, the script either prints that the hashes are identical or it prints an error message with the parameters resulting different hashes.

11.5 Determination of a BonnMotion app

For validating a BonnMotion app, the script needs a parameter file with four constant parameters. These parameters must be provided:

app	the name of the app
paramsfile	is the filename of a model parameter file or the path of a folder with several modeltest files
extensions	is a set of the suffixes the app creates
appparams	the parameters of the app

Sample apptest:

```
app=TheONEFile
paramsfile=randomwaypoint.testparams
extensions={one}
appparams=-l 2
```

The script runs BonnMotion with the specified testparams file (like with model determination) and then runs the app with the app-parameters over the output (in this case `-l 2`) and saves hashes (with BonnMotion-parameters and app-parameters). The script expects the app to accept a `-f` parameter to define the input file. The table name in which the hashes will be saved is the appname.

If you need to test multiple parameter values or parameters combinations several test cases can be defined. A test case can overwrite the `extensions` and `appparams` values.

Sample apptest with testcases:

```
app=LinkDump
paramsfile=modeltests-basic
extensions={ict_50.0;ld_50.0}
appparams=-r 50 -d -j

[testcase]
extensions={ict_100.0;ld_100.0}
appparams=-r 100 -d -j
```

11.6 Validation of a BonnMotion app

Validation of a BonnMotion app is similar to model validation but you have to specify an app name. All apps will be validated if no app name is provided.

12 Acknowledgments

The first versions of this software were designed and implemented by Michael Gerharz and Christian de Waal. Further extensions have been designed and implemented by Nils Aschenbruck, Jan-Hendrik Bolte, Alexander Bothe, Tracy Camp (CSM), Michael Coughlin (CSM), Raphael Ernst, Elmar Gerhards-Padilla, Christian Heiden, Tim Heinrich, Karina Meyer, Aarti Munjal (CSM), Patrick Peschlow, Gary Scheid (CSM), Florian Schmitt, Matthias Schwamborn, and Chris Walsh (CSM). Moreover, contributions to code and this documentation have been made by Juan G. Barros, Yue Cao, Elmano Ramalho Cavalcanti, Nuno Cruz, and Martin Giachino.

The development of the first versions was supported in part by the German Federal Ministry of Education and Research (BMBF) as part of the IPonAir project.

Since 2008, BonnMotion is partially supported by CONET, the Cooperating Objects Network of Excellence, funded by the European Commission under FP7 with contract number FP7-2007-2-224053.

The project has also been partially supported by the National Science Foundation (under CNS-0905513 and CNS-0848130). Any opinions, findings and conclusions, or recommendations expressed in this material do not necessarily reflect those of the National Science Foundation.

References

[1] N. Aschenbruck, R. Ernst, and P. Martini, "Indoor mobility modeling," in *Proceedings of the IEEE Global Communication Conference Workshop on Mobile Computing and Emerging Communication Networks MCECN, Miami, Florida, USA*, 2010.

[2] N. Aschenbruck, E. Gerhards-Padilla, M. Gerharz, M. Frank, and P. Martini, "Modelling Mobility in Disaster Area Scenarios," *Proc. 10th ACM-IEEE Int. Symposium on Modeling, Analysis and Simulation of Wireless and Mobile Systems (MSWIM)*, pp. 4–12, 2007.

[3] N. Aschenbruck and M. Schwamborn, "Synthetic map-based mobility traces for the performance evaluation in opportunistic networks," in *Proceedings of the 2nd International Workshop on Mobile Opportunistic Networking, MobiOpp 2010, Pisa, Italy, February 22-23, 2010.* ACM, 2010, pp. 143–146.

[4] F. Bai and A. Helmy, "A survey of mobility models," 2004, http://nile.usc.edu/~helmy/important/Modified-Chapter1-5-30-04.pdf.

[5] F. Bai, N. Sadagopan, and A. Helmy, "IMPORTANT: A framework to systematically analyze the Impact of Mobility on Performance of RouTing protocols for Adhoc NeTworks," in *Proc. of the IEEE Infocom*, 2003, pp. 825–835.

[6] C. Bettstetter, "Mobility modeling in wireless networks: categorization, smooth movement, and border effects," *ACM SIGMOBILE Mobile Computing and Communications Review*, vol. 5, no. 3, pp. 55–66, 2001.

[7] C. Bettstetter and C. Wagner, "The Spatial Node Distribution of the Random Waypoint Mobility Model," in *Proc. of the 1st German Workshop on Mobile Ad-Hoc Networks (WMAN'02)*, 2002, pp. 41–58.

[8] H. Butler, C. Schmidt, D. Springmeyer, and J. Livni, "Spatial Reference," 2013, http://spatialreference.org/.

[9] T. Camp, J. Boleng, and V. Davies, "A Survey of Mobility Models for Ad Hoc Network Research," *Wireless Communication and Mobile Computing (WCMC): Special issue on Mobile Ad Hoc Networking: Research, Trends and Applications*, vol. 2, no. 5, pp. 483–502, Sep. 2002.

[10] *Universal Mobile Telecommunicatios System (UMTS) - Selection procedures for the choice of radio transmission technologies of the UMTS*, Umts 30.03 version 3.2.0, tr 101 112 ed., European Telecommunications Standards Institute (ETSI), 1998.

[11] GPX Developers, "GPX 1.1 Schema Documentation," 2012, http://www.topografix.com/GPX/1/1/.

[12] X. Hong, M. Gerla, G. Pei, and C.-C. Chiang, "A Group Mobility Model for Ad Hoc Wireless Networks," *Proc. of the ACM Int. Workshop on Modelling and Simulation of Wireless and Mobile Systems (MSWiM)*, pp. 53–60, 1999.

[13] S. P. Hoogendoorn and P. H. L. Bovy, "State-of-the-art of vehicular traffic flow modelling," *Journal of Systems and Control Engineering - Special Issue on Road Traffic Modelling and Control*, vol. 215, no. 4, pp. 283–304, 2001.

[14] P. Johansson, T. Larsson, N. Hedman, B. Mielczarek, and M. Degermark, "Scenario-based Performance Analysis of Routing Protocols for Mobile Ad-hoc Networks," *Proc. of the Mobicom*, pp. 195–206, 1999.

[15] A. Keränen, J. Ott, and T. Kärkkäinen, "The one simulator for dtn protocol evaluation," in *Proceedings of the 2nd International Conference on Simulation Tools and Techniques, SIMUTools 2009, Rome, Italy, March 2-6, 2009.* ICST, 2009, pp. 1–10.

[16] K. Lee, S. Hong, S. J. Kim, I. Rhee, and S. Chong, "Slaw: A mobility model for human walks," in *Proc. of the 28th Int. Conference on Computer Communications (INFOCOM '09)*, 2009, pp. 855–863.

[17] B. Liang and Z. J. Haas, "Predictive distance-based mobility management for PCS networks," *Proc. of the IEEE Infocom*, pp. 1377–1384, 1999.

[18] A. Mei and J. Stefa, "SWIM: A simple model to generate small mobile worlds," *Proceedings - IEEE INFOCOM*, no. 215923, pp. 2106–2113, 2009.

[19] A. Mei, J. Stefa, and S. Kosta, "Swim: Small world in motion - a mobility model for human movements," 2014, http://swim.di.uniroma1.it/.

[20] A. Morton, "DMAP: UTM Grid Zones of the World," 2013, http://www.dmap.co.uk/utmworld.htm.

[21] A. Munjal, T. Camp, and W. C. Navidi, "Smooth: A simple way to model human walks," in *Proceedings of the 14th ACM International Conference on Modeling, Analysis and Simulation of Wireless and Mobile Systems (MSWiM)*, 2011.

[22] M. Musolesi and C. Mascolo, "Mobility models for systems evaluation," *State of the Art on Middleware for Network Eccentric and Mobile Applications (MINEMA)*, 2008.

[23] W. Navidi and T. Camp, "Stationary distributions for the random waypoint mobility model," *IEEE Transactions on Mobile Computing*, vol. 3, no. 1, pp. 99–108, Jan-Feb 2004.

[24] W. Navidi, T. Camp, and N. Bauer, "Improving the Accuracy of Random Waypoint Simulations through Steady-State Initialization," in *Proceedings of the 15th International Conference on Modeling and Simulation*, 2004, pp. 319–326.

[25] NCSU Networking Research Lab, "Human mobility models download," 2011, http://research.csc.ncsu.edu/netsrv/?q=content/human-mobility-models-download-tlw-slaw.

[26] OGP Surveying and Positioning Committee, "EPSG Geodetic Parameter Registry," 2013, http://www.epsg-registry.org/.

[27] I. Rhee, M. Shin, S. Hong, K. Lee, and S. Chong, "On the levy-walk nature of human mobility," in *Proceeding of the 27th Conference on Computer Communications (INFOCOM 2008)*, April 2008, pp. –.

[28] M. Schwamborn and N. Aschenbruck, “Introducing Geographic Restrictions to the SLAW Human Mobility Model,” in *Proc. of the 21st Int. Symposium on Modeling, Analysis and Simulation of Computer and Telecommunication Systems (MASCOTS ’13)*, San Francisco, CA, USA, 2013, pp. 264–272.

[29] ——, “On Modeling and Impact of Geographic Restrictions for Human Mobility in Opportunistic Networks,” in *Proc. of the 23rd Int. Symposium on Modeling, Analysis and Simulation of Computer and Telecommunication Systems (MASCOTS ’15)*, Atlanta, GA, USA, 2015, pp. 178–187.

[30] TOILERS, “Smooth: A simple way to model human mobility,” 2012, http://toilers.mines.edu/Public/Code/smooth.html.

[31] WISEBED Project, “Deliverable d4.1: First set of well-designed simulations, experiments and possible benchmarks,” 2009, http://www.wisebed.eu/images/stories/deliverables/d4.1.pdf.
