# Supplementary material for: An empirical evaluation of link quality utilization in ETX routing for VANETs
Source: PeerJ Comput Sci. 2024 Sep 6;10:e2259. doi: 10.7717/peerj-cs.2259 (PMC11419655; doi:10.7717/peerj-cs.2259)
Supplement: Supplemental Information 1 [file peerj-cs-10-2259-s001.bz2 › ns-allinone-3.29/ns-3.29/src/spectrum/doc/spectrum-channel-phy-interface.pdf]

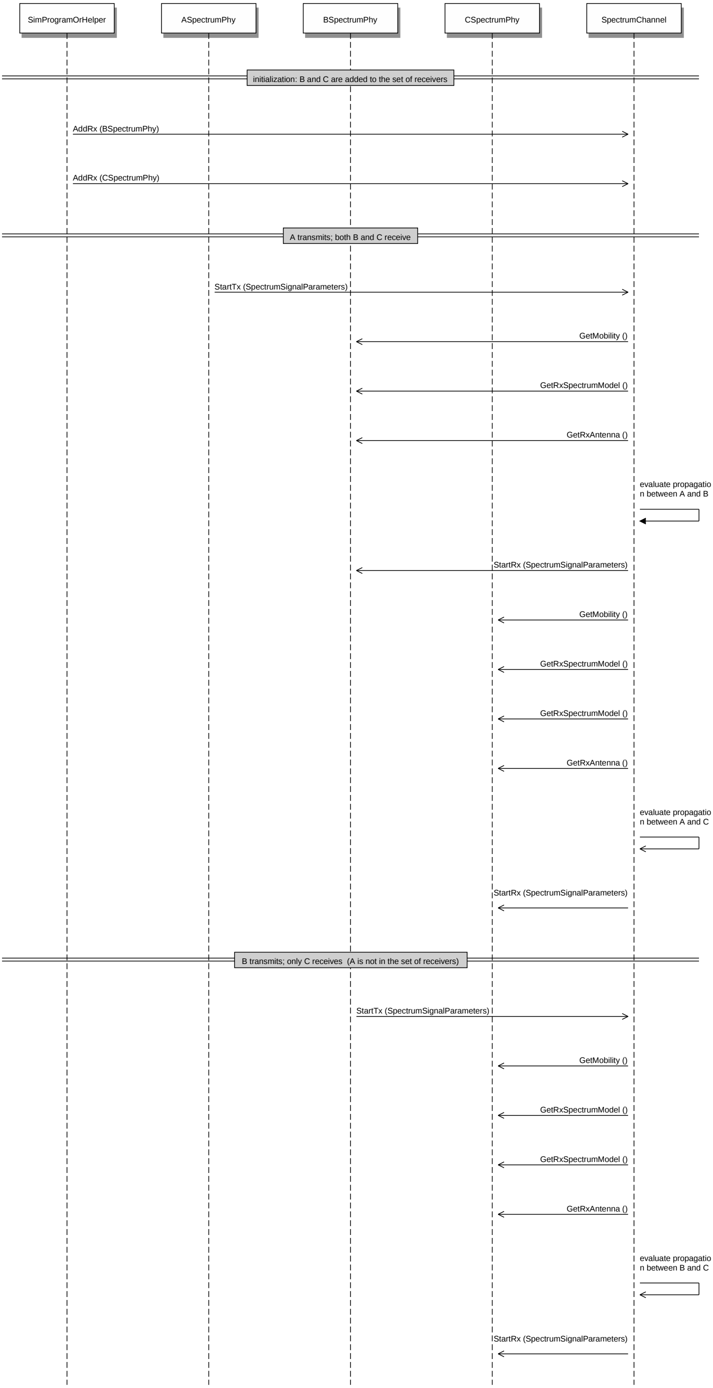
SimProgramOrHelper
ASpectrumPhy
BSpectrumPhy
CSpectrumPhy
SpectrumChannel
initialization: B and C are added to the set of receivers
AddRx (BSpectrumPhy)
AddRx (CSpectrumPhy)
A transmits; both B and C receive
StartTx (SpectrumSignalParameters)
GetMobility ()
GetRxSpectrumModel ()
GetRxAntenna ()
evaluate propagatio
n between A and B
StartRx (SpectrumSignalParameters)
GetMobility ()
GetRxSpectrumModel ()
GetRxSpectrumModel ()
GetRxAntenna ()
evaluate propagatio
n between A and C
StartRx (SpectrumSignalParameters)
B transmits; only C receives (A is not in the set of receivers)
StartTx (SpectrumSignalParameters)
GetMobility ()
GetRxSpectrumModel ()
GetRxSpectrumModel ()
GetRxAntenna ()
evaluate propagatio
n between B and C
StartRx (SpectrumSignalParameters)
