# Supplementary material for: An empirical evaluation of link quality utilization in ETX routing for VANETs
Source: PeerJ Comput Sci. 2024 Sep 6;10:e2259. doi: 10.7717/peerj-cs.2259 (PMC11419655; doi:10.7717/peerj-cs.2259)
Supplement: Supplemental Information 1 [file peerj-cs-10-2259-s001.bz2 › ns-allinone-3.29/ns-3.29/src/lte/test/reference/lte-mcs-index.pdf]

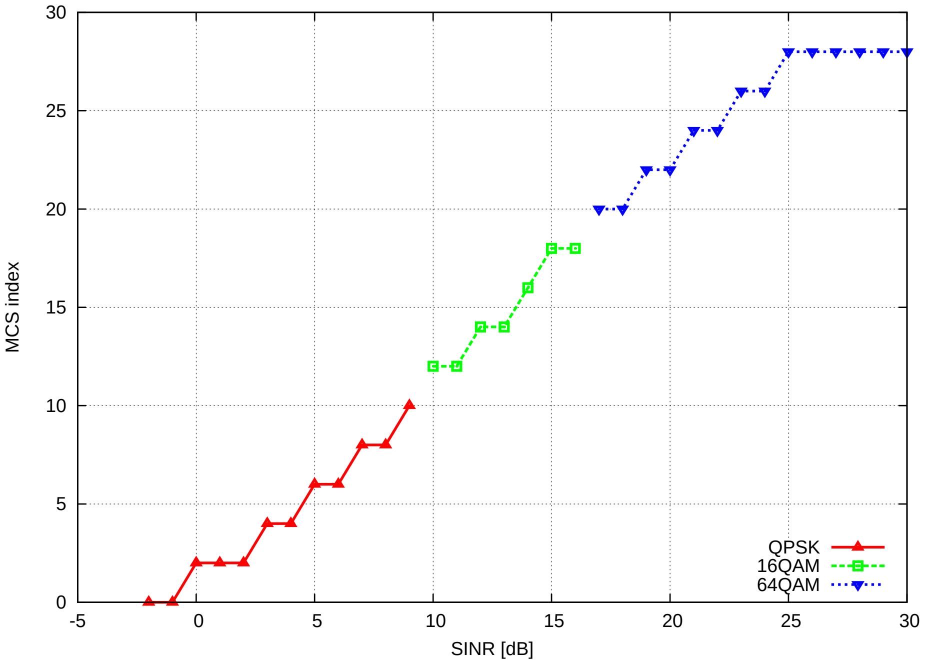

MCS index
SINR [dB]
QPSK
16QAM
64QAM
0
5
10
15
20
25
30
-5
0
5
10
15
20
25
30
