# Supplementary material for: An empirical evaluation of link quality utilization in ETX routing for VANETs
Source: PeerJ Comput Sci. 2024 Sep 6;10:e2259. doi: 10.7717/peerj-cs.2259 (PMC11419655; doi:10.7717/peerj-cs.2259)
Supplement: Supplemental Information 1 [file peerj-cs-10-2259-s001.bz2 › ns-allinone-3.29/ns-3.29/src/lte/doc/source/figures/nas-attach.pdf]

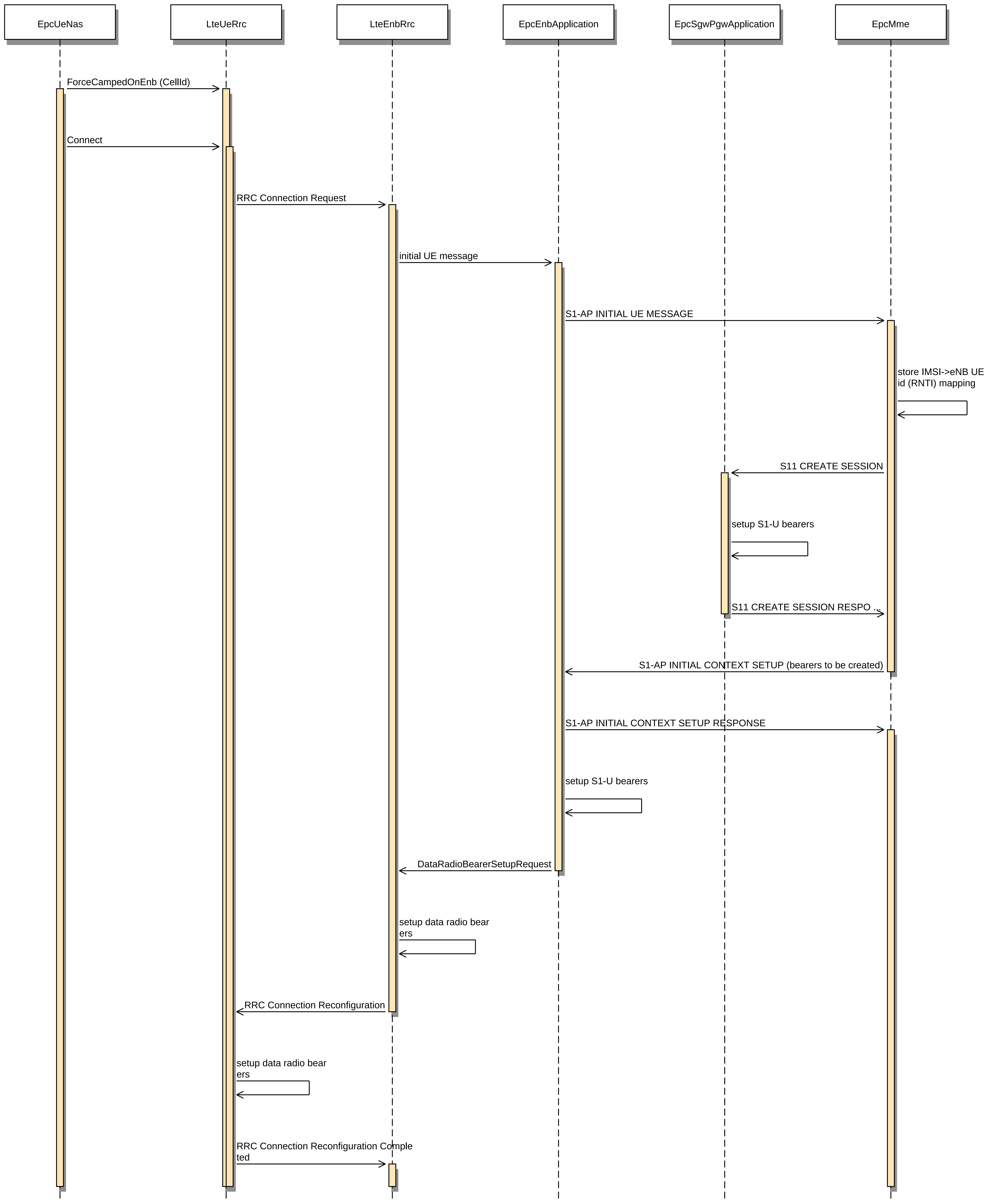
EpcUeNas
LteUeRrc
LteEnbRrc
EpcEnbApplication
EpcSgwPgwApplication
EpcMme
ForceCampedOnEnb (CellId)
Connect
RRC Connection Request
initial UE message
S1-AP INITIAL UE MESSAGE
store IMSI->eNB UE id (RNTI) mapping
S11 CREATE SESSION
setup S1-U bearers
S11 CREATE SESSION RESPO ...
S1-AP INITIAL CONTEXT SETUP (bearers to be created)
S1-AP INITIAL CONTEXT SETUP RESPONSE
setup S1-U bearers
DataRadioBearerSetupRequest
setup data radio bear ers
RRC Connection Reconfiguration
setup data radio bear ers
RRC Connection Reconfiguration Comple ted
