# Supplementary material for: An empirical evaluation of link quality utilization in ETX routing for VANETs
Source: PeerJ Comput Sci. 2024 Sep 6;10:e2259. doi: 10.7717/peerj-cs.2259 (PMC11419655; doi:10.7717/peerj-cs.2259)
Supplement: Supplemental Information 1 [file peerj-cs-10-2259-s001.bz2 › ns-allinone-3.29/ns-3.29/src/lte/doc/source/figures/MCS_12_test.pdf]

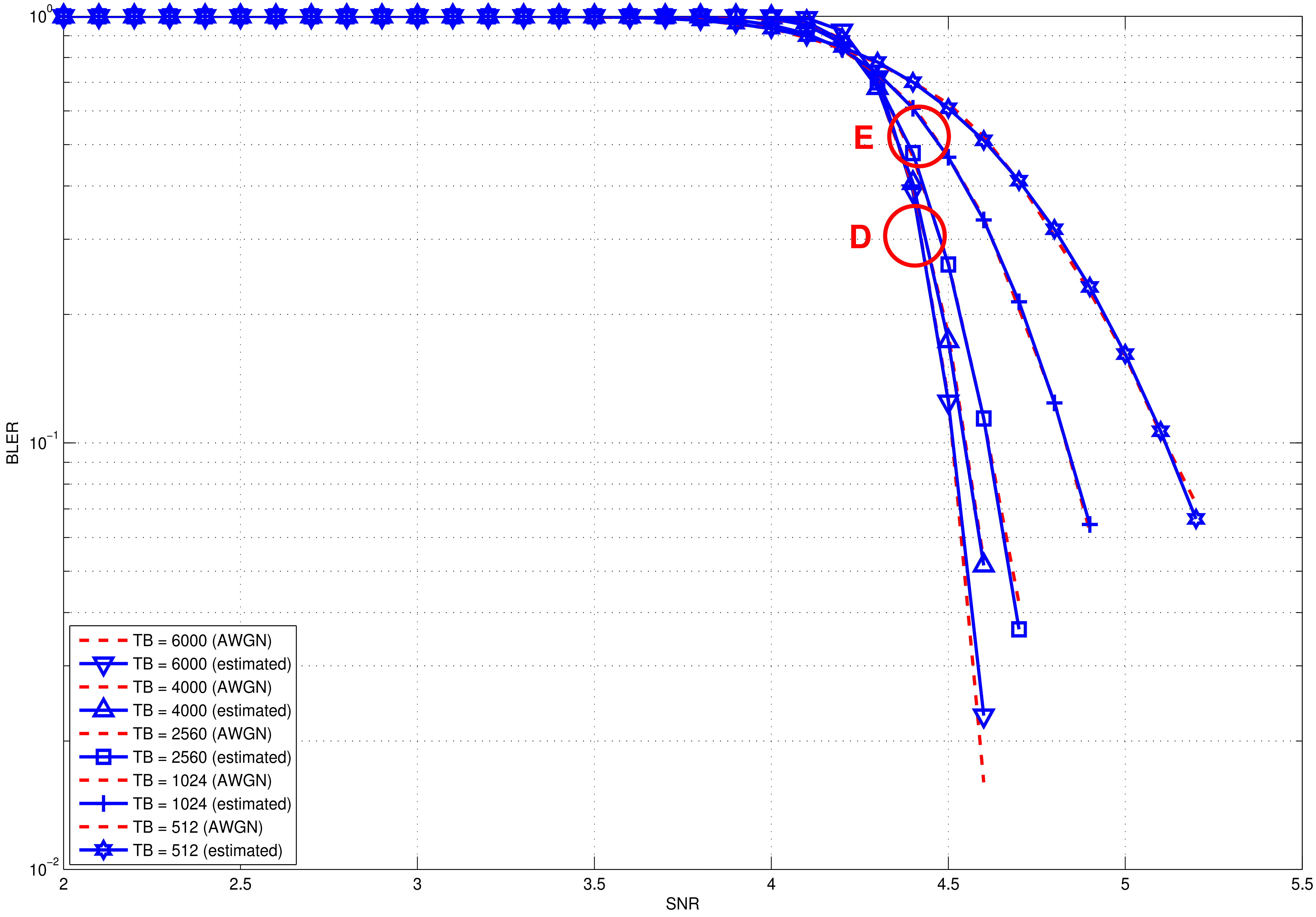

BLER
SNR
10^0
10^{-1}
10^{-2}
2
2.5
3
3.5
4
4.5
5
5.5
E
D
TB = 6000 (AWGN)
TB = 6000 (estimated)
TB = 4000 (AWGN)
TB = 4000 (estimated)
TB = 2560 (AWGN)
TB = 2560 (estimated)
TB = 1024 (AWGN)
TB = 1024 (estimated)
TB = 512 (AWGN)
TB = 512 (estimated)
