# Supplementary material for: An empirical evaluation of link quality utilization in ETX routing for VANETs
Source: PeerJ Comput Sci. 2024 Sep 6;10:e2259. doi: 10.7717/peerj-cs.2259 (PMC11419655; doi:10.7717/peerj-cs.2259)
Supplement: Supplemental Information 1 [file peerj-cs-10-2259-s001.bz2 › ns-allinone-3.29/ns-3.29/src/lte/doc/source/figures/rrc-connection-reconfiguration-handover.pdf]

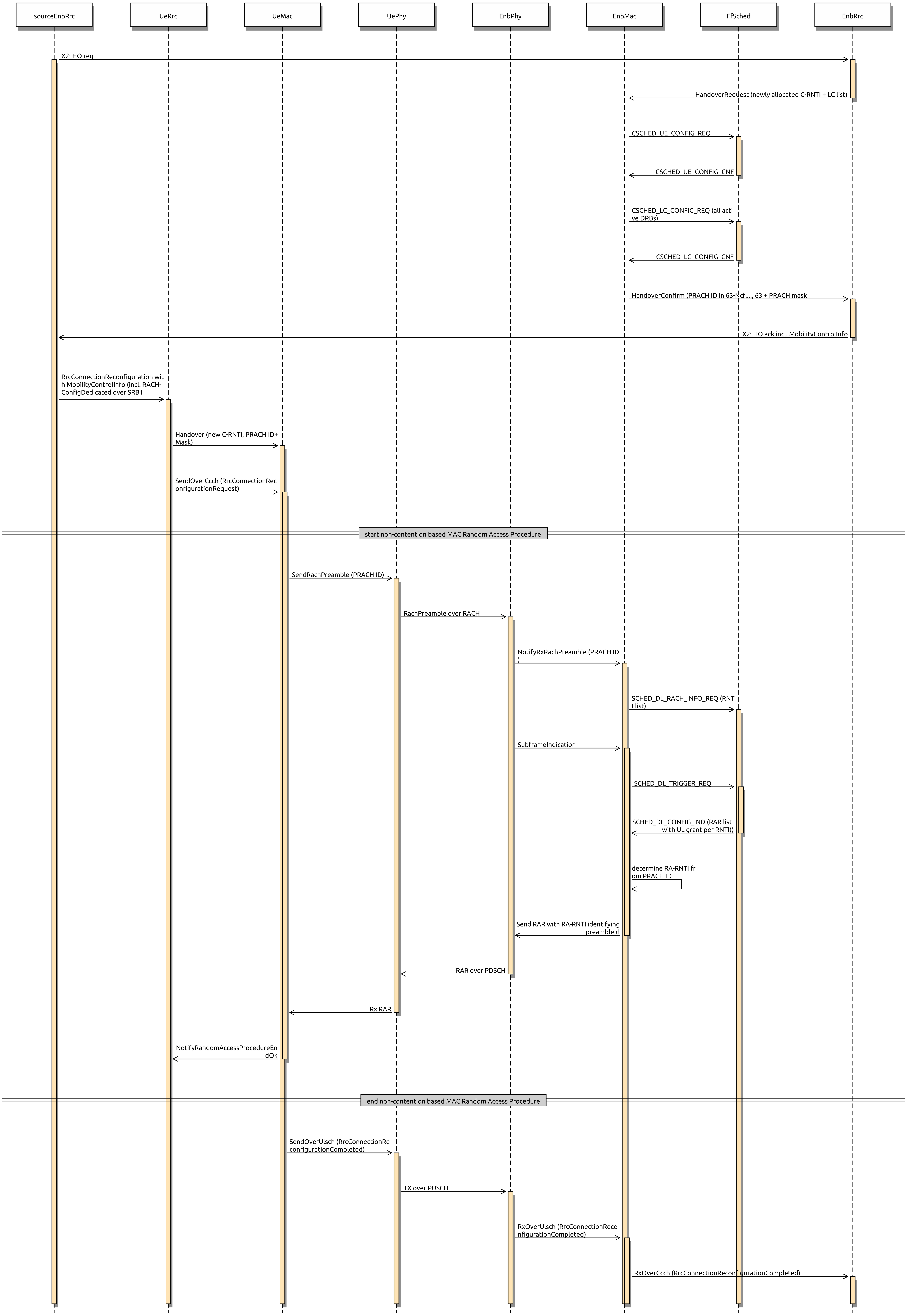
sourceEnbRrc
UeRrc
UeMac
UePhy
EnbPhy
EnbMac
FfSched
EnbRrc
X2: HO req
HandoverRequest (newly allocated C-RNTI + LC list)
CSCHED_UE_CONFIG_REQ
CSCHED_UE_CONFIG_CNF
CSCHED_LC_CONFIG_REQ (all acti
ve DRBs)
CSCHED_LC_CONFIG_CNF
HandoverConfirm (PRACH ID in 63-Ncf,..., 63 + PRACH mask
X2: HO ack incl. MobilityControlInfo
RrcConnectionReconfiguration wit
h MobilityControlInfo (incl. RACH-
ConfigDedicated over SRB1
Handover (new C-RNTI, PRACH ID+
Mask)
SendOverCcch (RrcConnectionRec
onfigurationRequest)
start non-contention based MAC Random Access Procedure
SendRachPreamble (PRACH ID)
RachPreamble over RACH
NotifyRxRachPreamble (PRACH ID
)
SCHED_DL_RACH_INFO_REQ (RNT
I list)
SubframeIndication
SCHED_DL_TRIGGER_REQ
SCHED_DL_CONFIG_IND (RAR list
with UL grant per RNTI))
determine RA-RNTI fr
om PRACH ID
Send RAR with RA-RNTI identifying
preambleId
RAR over PDSCH
Rx RAR
NotifyRandomAccessProcedureEn
dOk
end non-contention based MAC Random Access Procedure
SendOverUlsch (RrcConnectionRe
configurationCompleted)
TX over PUSCH
RxOverUlsch (RrcConnectionReco
nfigurationCompleted)
RxOverCcch (RrcConnectionReconfigurationCompleted)
