# Supplementary material for: An empirical evaluation of link quality utilization in ETX routing for VANETs
Source: PeerJ Comput Sci. 2024 Sep 6;10:e2259. doi: 10.7717/peerj-cs.2259 (PMC11419655; doi:10.7717/peerj-cs.2259)
Supplement: Supplemental Information 1 [file peerj-cs-10-2259-s001.bz2 › ns-allinone-3.29/ns-3.29/src/lte/doc/source/figures/mac-random-access-contention.pdf]

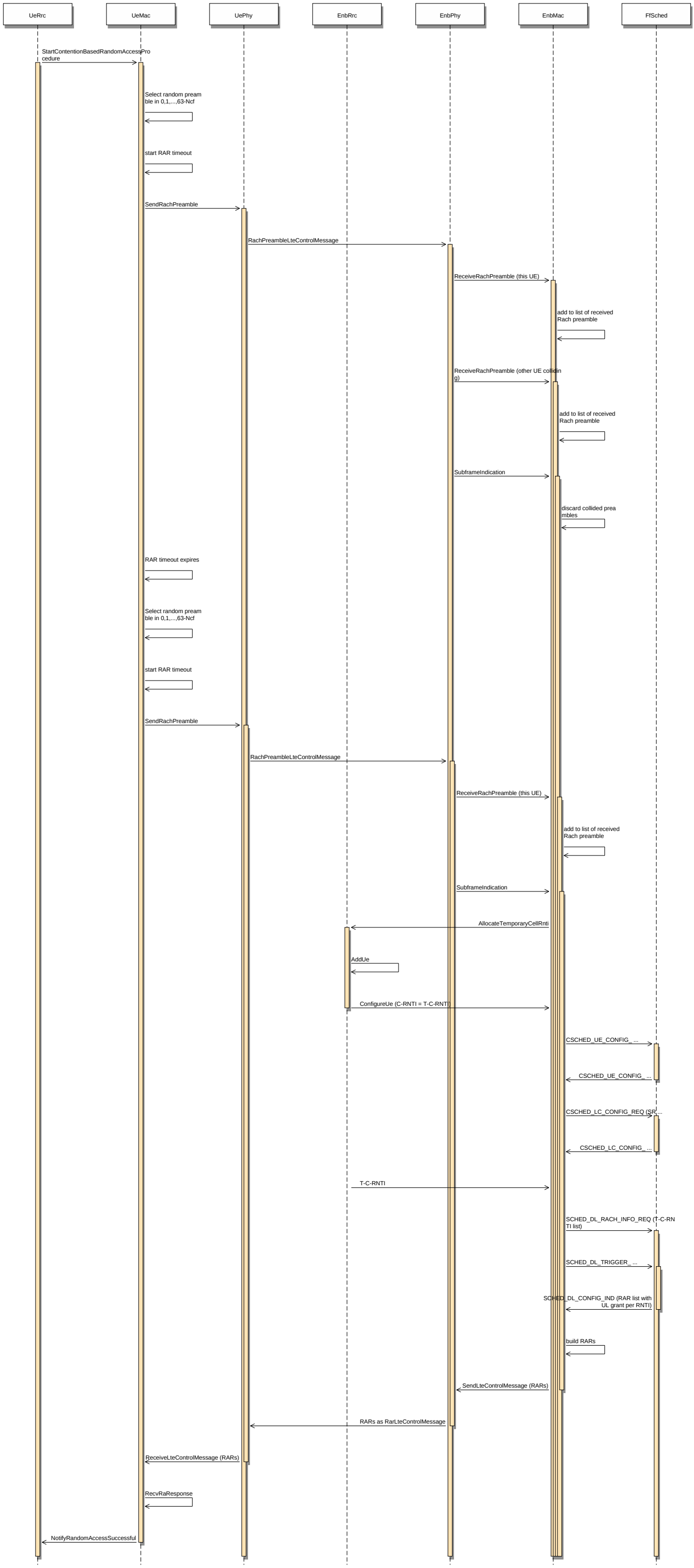
UeRrc
UeMac
UePhy
EnbRrc
EnbPhy
EnbMac
FfSched
StartContentionBasedRandomAccessPro
cedure
Select random pream
ble in 0,1,...,63-Ncf
start RAR timeout
SendRachPreamble
RachPreambleLteControlMessage
ReceiveRachPreamble (this UE)
add to list of received
Rach preamble
ReceiveRachPreamble (other UE collidin
g)
add to list of received
Rach preamble
SubframeIndication
discard collided prea
mbles
RAR timeout expires
Select random pream
ble in 0,1,...,63-Ncf
start RAR timeout
SendRachPreamble
RachPreambleLteControlMessage
ReceiveRachPreamble (this UE)
add to list of received
Rach preamble
SubframeIndication
AllocateTemporaryCellRnti
AddUe
ConfigureUe (C-RNTI = T-C-RNTI)
CSCHED_UE_CONFIG_ ...
CSCHED_UE_CONFIG_ ...
CSCHED_LC_CONFIG_REQ (SR...
CSCHED_LC_CONFIG_ ...
T-C-RNTI
SCHED_DL_RACH_INFO_REQ (T-C-RN
TI list)
SCHED_DL_TRIGGER_ ...
SCHED_DL_CONFIG_IND (RAR list with
UL grant per RNTI)
build RARs
SendLteControlMessage (RARs)
RARs as RarLteControlMessage
ReceiveLteControlMessage (RARs)
RecvRaResponse
NotifyRandomAccessSuccessful
