# Supplementary material for: An empirical evaluation of link quality utilization in ETX routing for VANETs
Source: PeerJ Comput Sci. 2024 Sep 6;10:e2259. doi: 10.7717/peerj-cs.2259 (PMC11419655; doi:10.7717/peerj-cs.2259)
Supplement: Supplemental Information 1 [file peerj-cs-10-2259-s001.bz2 › ns-allinone-3.29/ns-3.29/src/lte/doc/source/figures/lte-ffr-soft-2-spectrum-trace.pdf]

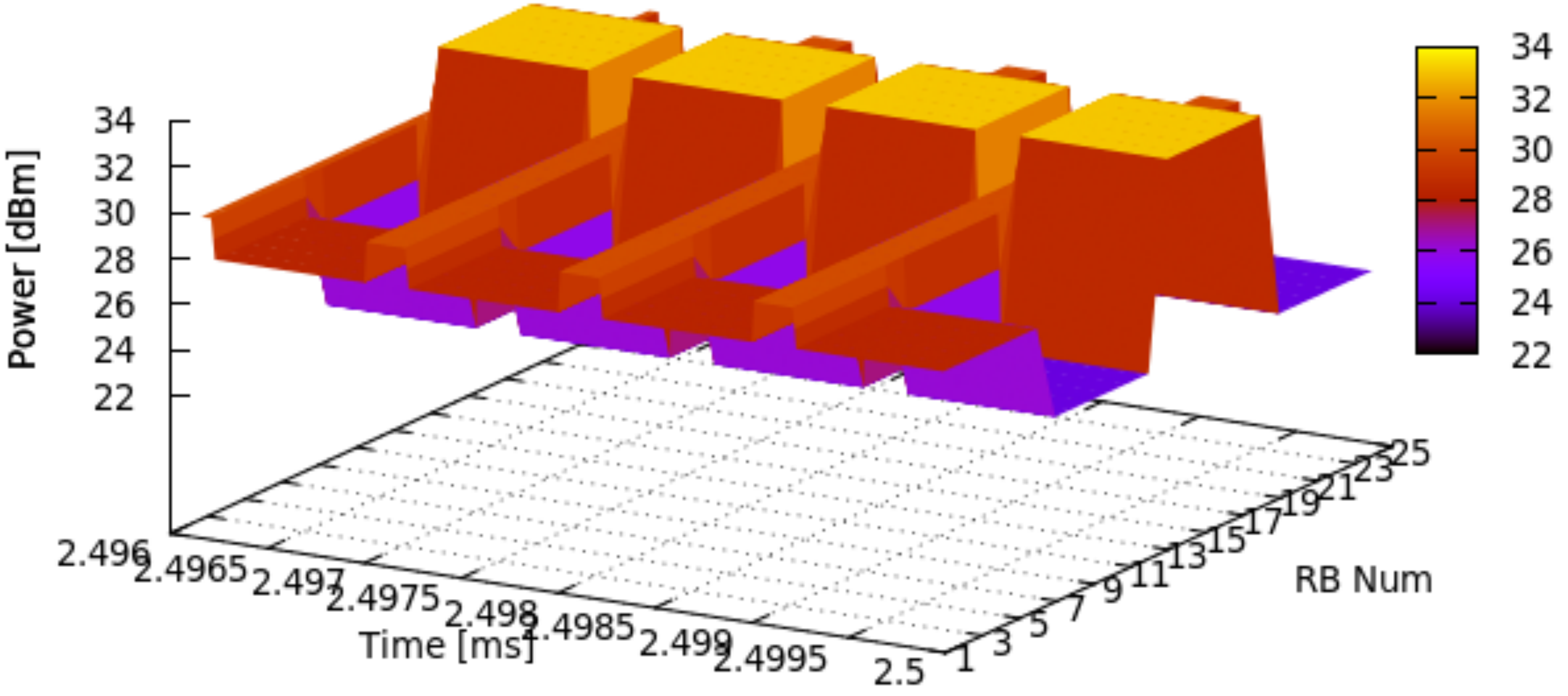
Power [dBm]
34
32
30
28
26
24
22
2.496
2.4965
2.497
2.4975
2.498
2.4985
2.499
2.4995
2.5
Time [ms]
1
3
5
7
9
11
13
15
17
19
21
23
25
RB Num
