# Supplementary material for: An empirical evaluation of link quality utilization in ETX routing for VANETs
Source: PeerJ Comput Sci. 2024 Sep 6;10:e2259. doi: 10.7717/peerj-cs.2259 (PMC11419655; doi:10.7717/peerj-cs.2259)
Supplement: Supplemental Information 1 [file peerj-cs-10-2259-s001.bz2 › ns-allinone-3.29/ns-3.29/src/lte/doc/source/figures/ca-uplink-bsr.pdf]

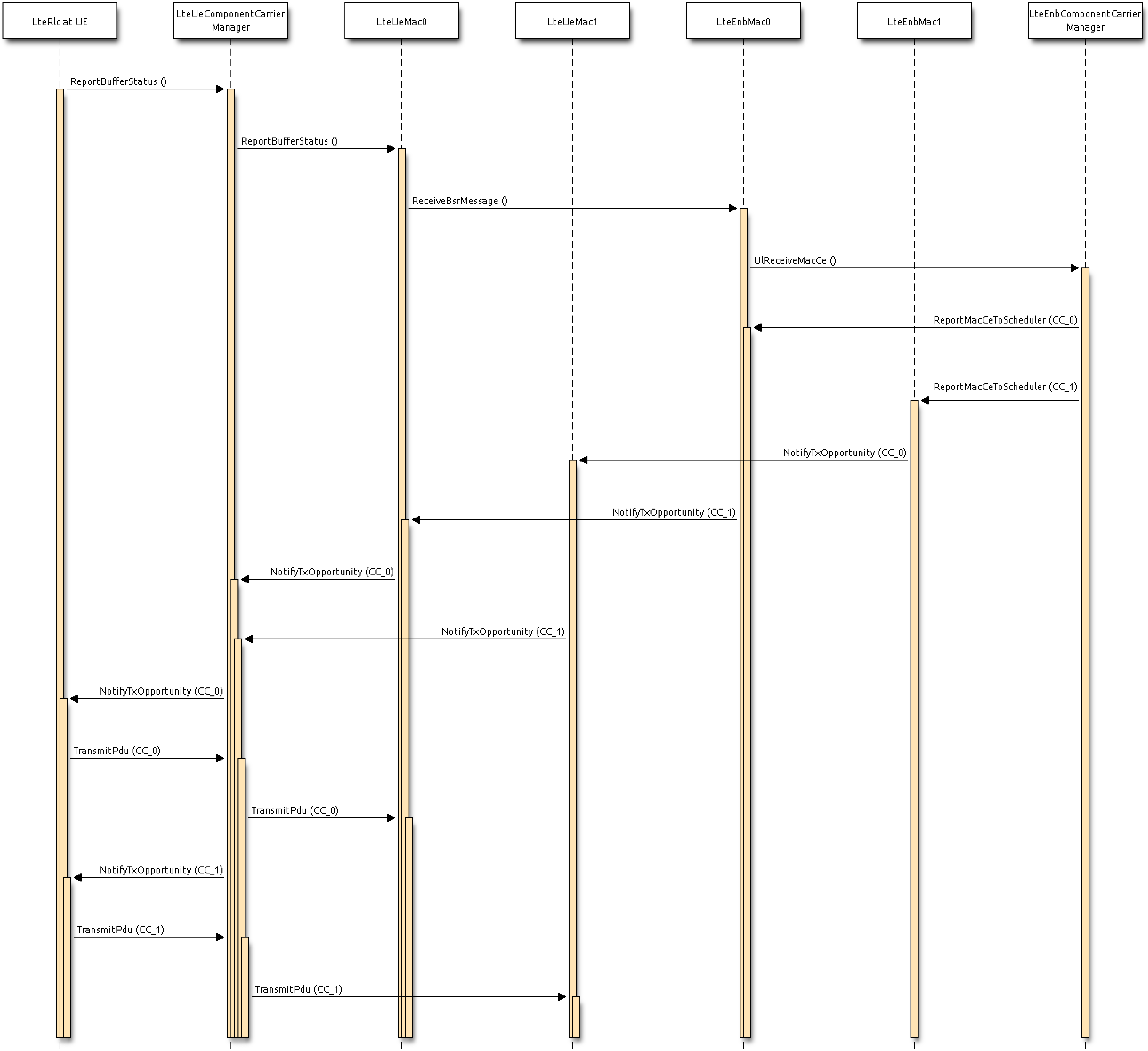

LteRlc at UE
LteUeComponentCarrier Manager
LteUeMac0
LteUeMac1
LteEnbMac0
LteEnbMac1
LteEnbComponentCarrier Manager
ReportBufferStatus ()
ReportBufferStatus ()
ReceiveBsrMessage ()
UlReceiveMacCe ()
ReportMacCeToScheduler (CC_0)
ReportMacCeToScheduler (CC_1)
NotifyTxOpportunity (CC_0)
NotifyTxOpportunity (CC_1)
NotifyTxOpportunity (CC_0)
NotifyTxOpportunity (CC_1)
NotifyTxOpportunity (CC_0)
TransmitPdu (CC_0)
TransmitPdu (CC_0)
NotifyTxOpportunity (CC_1)
TransmitPdu (CC_1)
TransmitPdu (CC_1)
