# Supplementary material for: An empirical evaluation of link quality utilization in ETX routing for VANETs
Source: PeerJ Comput Sci. 2024 Sep 6;10:e2259. doi: 10.7717/peerj-cs.2259 (PMC11419655; doi:10.7717/peerj-cs.2259)
Supplement: Supplemental Information 1 [file peerj-cs-10-2259-s001.bz2 › ns-allinone-3.29/ns-3.29/src/lte/doc/source/figures/lte-fr-hard-2-rem.pdf]

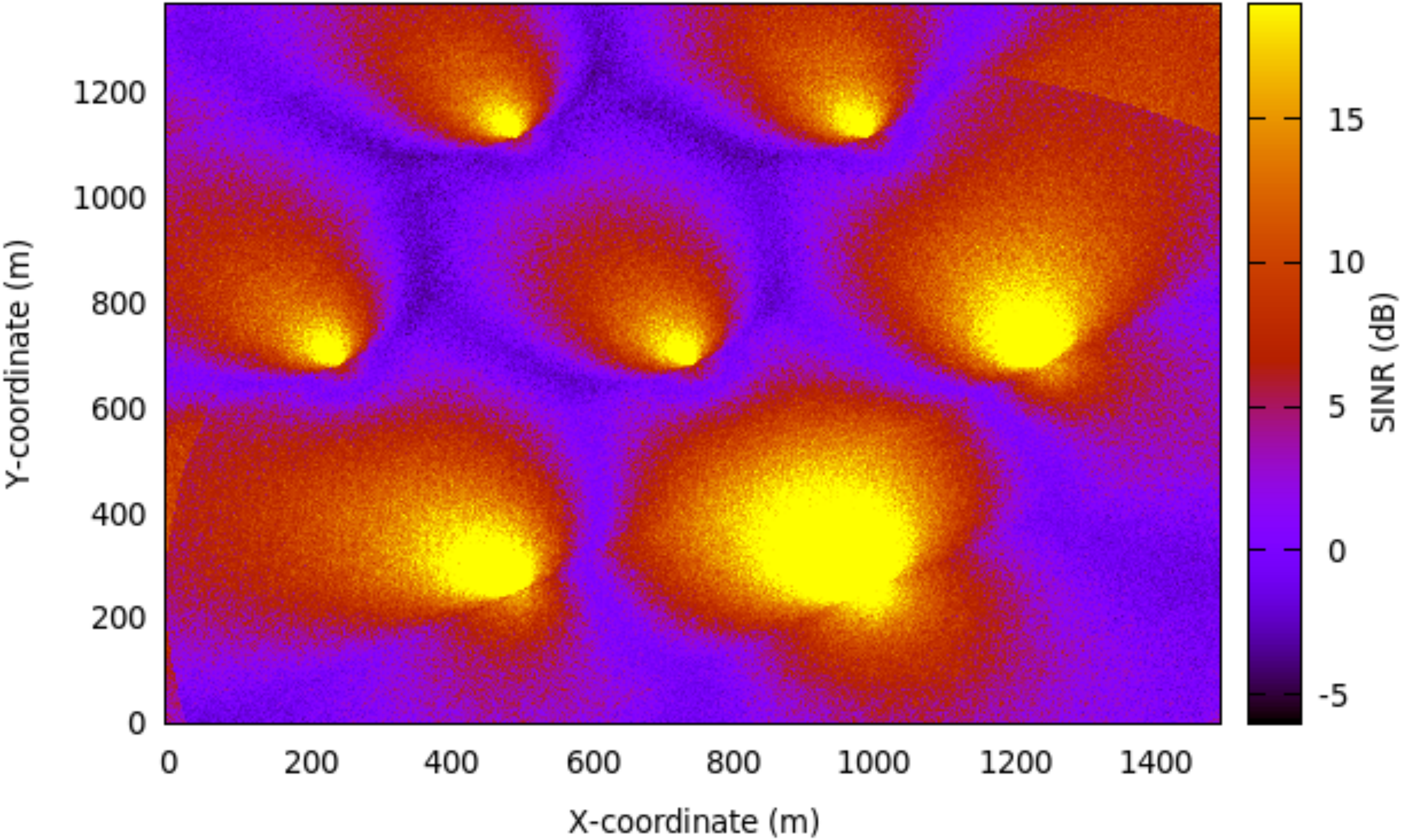

1200
1000
800
600
400
200
0
Y-coordinate (m)
0
200
400
600
800
1000
1200
1400
X-coordinate (m)
15
10
5
0
-5
SINR (dB)
