# Supplementary material for: An empirical evaluation of link quality utilization in ETX routing for VANETs
Source: PeerJ Comput Sci. 2024 Sep 6;10:e2259. doi: 10.7717/peerj-cs.2259 (PMC11419655; doi:10.7717/peerj-cs.2259)
Supplement: Supplemental Information 1 [file peerj-cs-10-2259-s001.bz2 › ns-allinone-3.29/ns-3.29/src/lte/doc/source/figures/miesm_scheme.pdf]

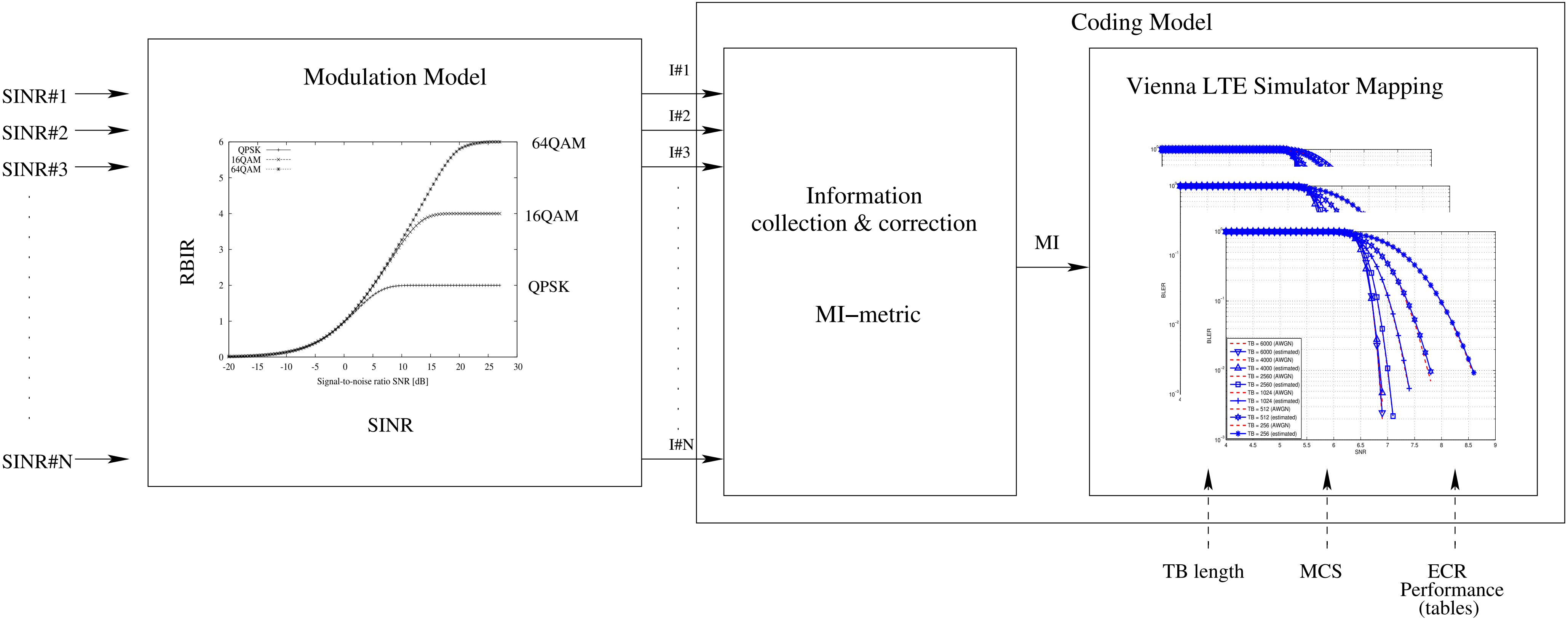
Coding Model
Modulation Model
SINR#1
SINR#2
SINR#3
SINR#N
QPSK
16QAM
64QAM
RBIR
Signal-to-noise ratio SNR [dB]
SINR
I#1
I#2
I#3
I#N
Information
collection & correction
MI–metric
MI
Vienna LTE Simulator Mapping
BLER
SNR
TB = 6000 (AWGN)
TB = 6000 (estimated)
TB = 4000 (AWGN)
TB = 4000 (estimated)
TB = 2560 (AWGN)
TB = 2560 (estimated)
TB = 1024 (AWGN)
TB = 1024 (estimated)
TB = 512 (AWGN)
TB = 512 (estimated)
TB = 256 (AWGN)
TB = 256 (estimated)
TB length
MCS
ECR
Performance
(tables)
