# Supplementary material for: An empirical evaluation of link quality utilization in ETX routing for VANETs
Source: PeerJ Comput Sci. 2024 Sep 6;10:e2259. doi: 10.7717/peerj-cs.2259 (PMC11419655; doi:10.7717/peerj-cs.2259)
Supplement: Supplemental Information 1 [file peerj-cs-10-2259-s001.bz2 › ns-allinone-3.29/ns-3.29/src/lte/doc/source/figures/ca-test-example-dl.pdf]

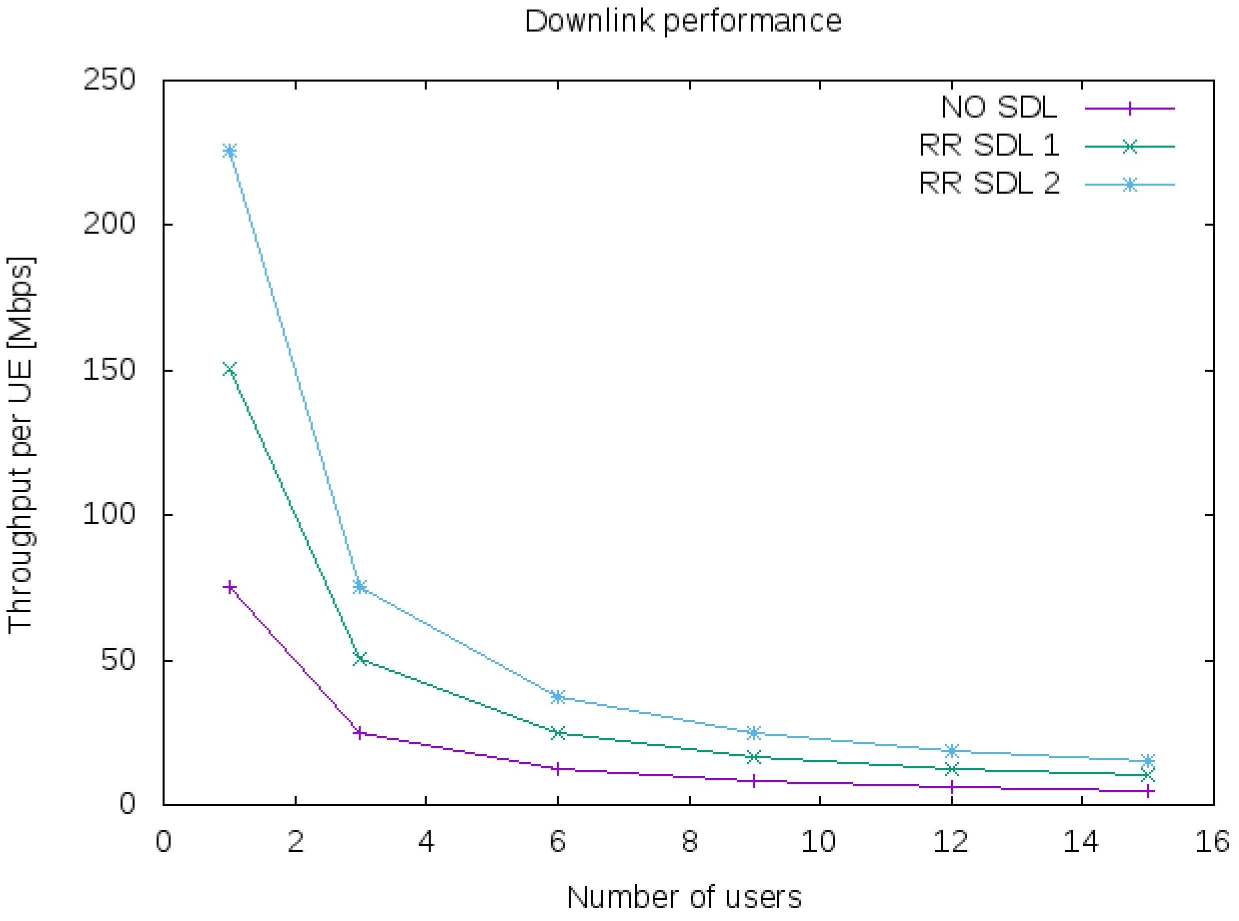

Downlink performance
NO SDL
RR SDL 1
RR SDL 2
Throughput per UE [Mbps]
Number of users
0
50
100
150
200
250
0
2
4
6
8
10
12
14
16
