# Supplementary material for: An empirical evaluation of link quality utilization in ETX routing for VANETs
Source: PeerJ Comput Sci. 2024 Sep 6;10:e2259. doi: 10.7717/peerj-cs.2259 (PMC11419655; doi:10.7717/peerj-cs.2259)
Supplement: Supplemental Information 1 [file peerj-cs-10-2259-s001.bz2 › ns-allinone-3.29/ns-3.29/src/lte/doc/source/figures/carrier-aggregation-mac-impact.pdf]

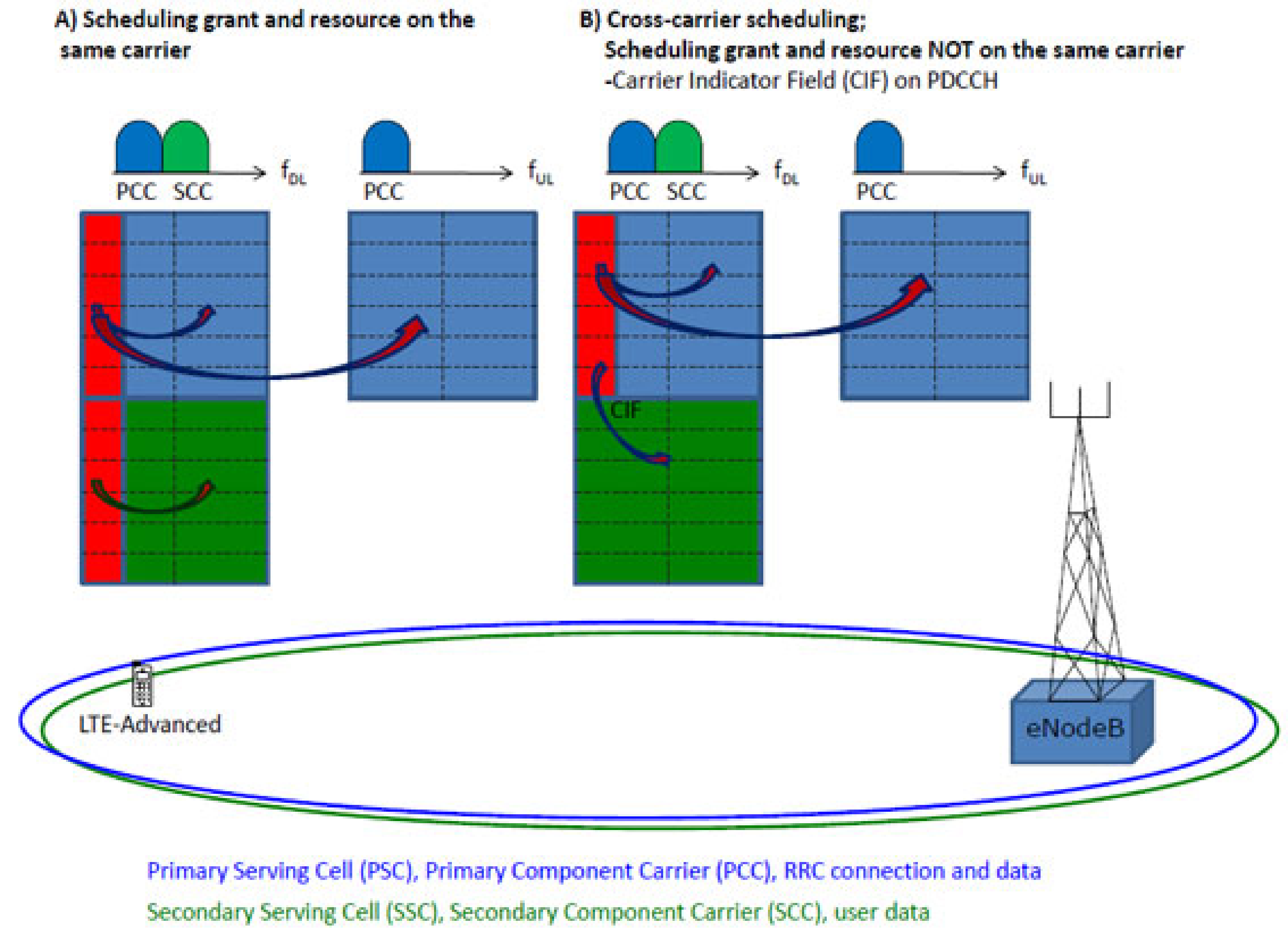
A) Scheduling grant and resource on the same carrier
B) Cross-carrier scheduling;
Scheduling grant and resource NOT on the same carrier
-Carrier Indicator Field (CIF) on PDCCH
PCC SCC
fDL
PCC
fUL
PCC SCC
fDL
PCC
fUL
CIF
LTE-Advanced
eNodeB
Primary Serving Cell (PSC), Primary Component Carrier (PCC), RRC connection and data
Secondary Serving Cell (SSC), Secondary Component Carrier (SCC), user data
