# Supplementary material for: An empirical evaluation of link quality utilization in ETX routing for VANETs
Source: PeerJ Comput Sci. 2024 Sep 6;10:e2259. doi: 10.7717/peerj-cs.2259 (PMC11419655; doi:10.7717/peerj-cs.2259)
Supplement: Supplemental Information 1 [file peerj-cs-10-2259-s001.bz2 › ns-allinone-3.29/ns-3.29/src/lte/doc/source/figures/rrc-connection-establishment.pdf]

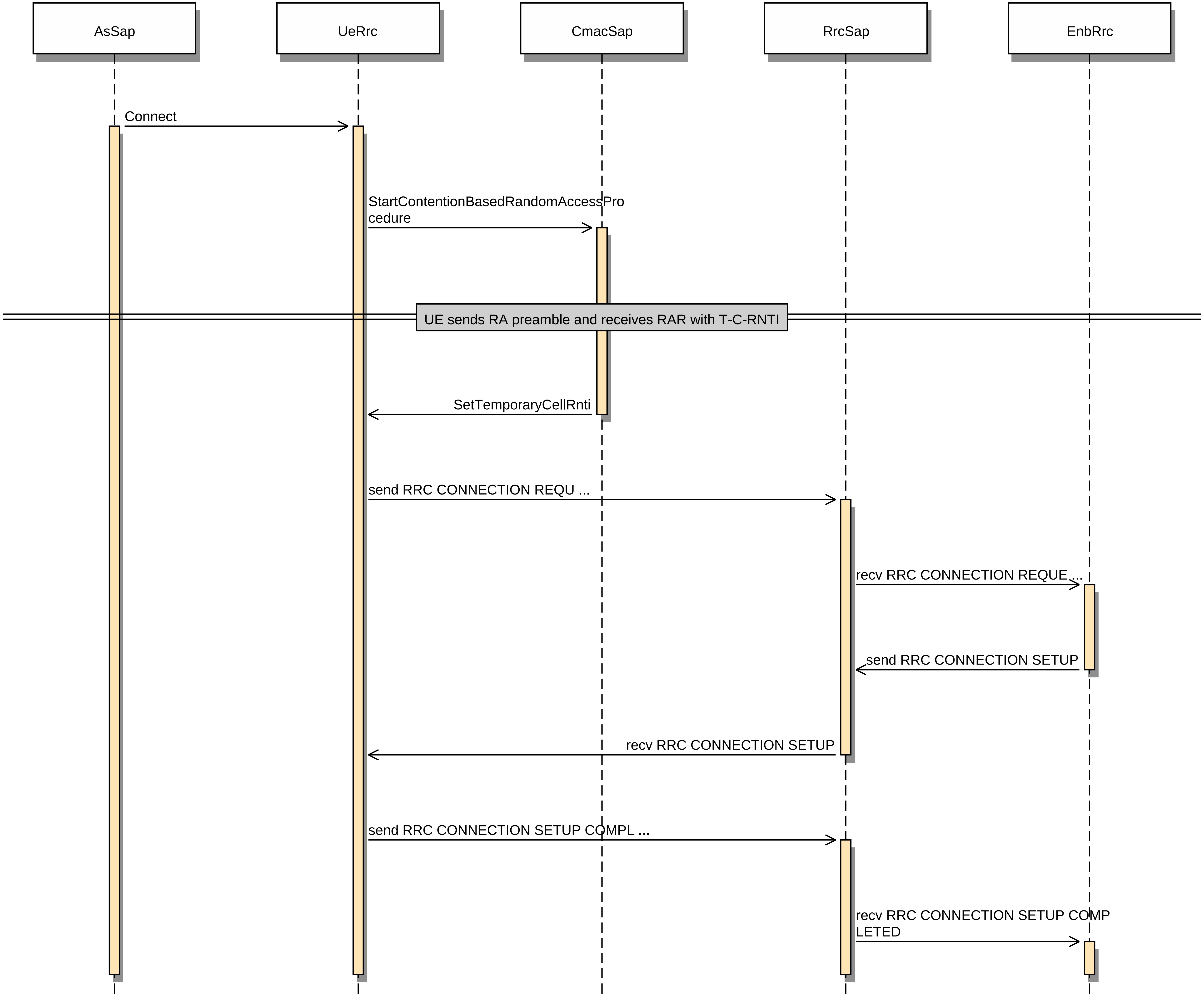
AsSap
UeRrc
CmacSap
RrcSap
EnbRrc
Connect
StartContentionBasedRandomAccessPro
cedure
UE sends RA preamble and receives RAR with T-C-RNTI
SetTemporaryCellRnti
send RRC CONNECTION REQU ...
recv RRC CONNECTION REQUE ...
send RRC CONNECTION SETUP
recv RRC CONNECTION SETUP
send RRC CONNECTION SETUP COMPL ...
recv RRC CONNECTION SETUP COMP
LETED
