# Supplementary material for: An empirical evaluation of link quality utilization in ETX routing for VANETs
Source: PeerJ Comput Sci. 2024 Sep 6;10:e2259. doi: 10.7717/peerj-cs.2259 (PMC11419655; doi:10.7717/peerj-cs.2259)
Supplement: Supplemental Information 1 [file peerj-cs-10-2259-s001.bz2 › ns-allinone-3.29/ns-3.29/src/lte/doc/source/figures/helpers.pdf]

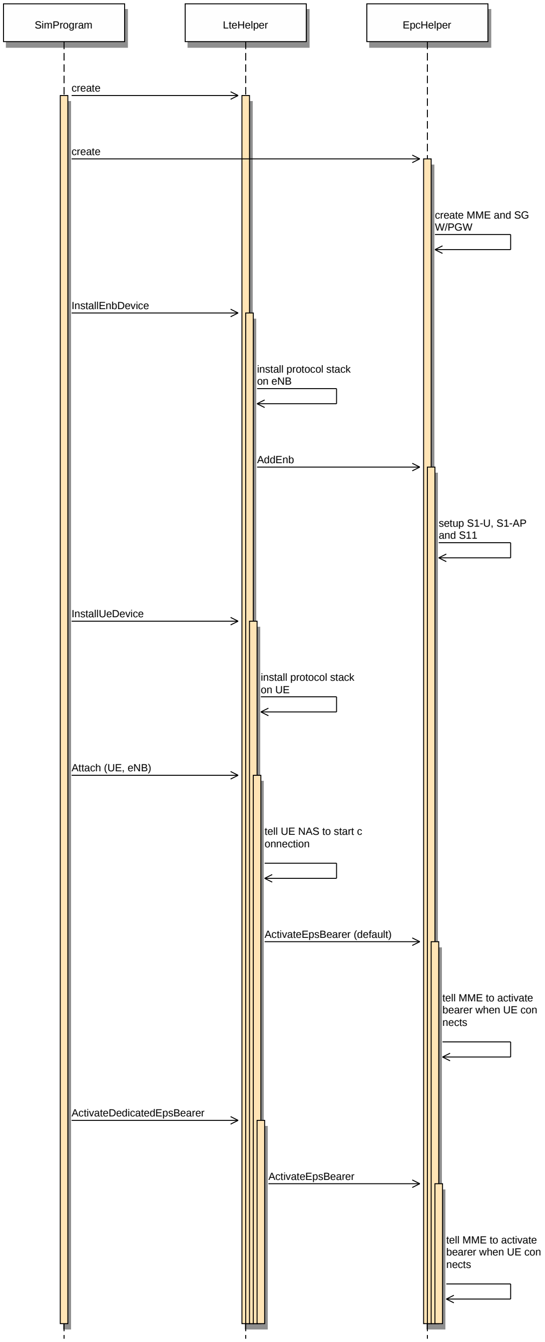
SimProgram
LteHelper
EpcHelper
create
create
create MME and SG
W/PGW
InstallEnbDevice
install protocol stack
on eNB
AddEnb
setup S1-U, S1-AP
and S11
InstallUeDevice
install protocol stack
on UE
Attach (UE, eNB)
tell UE NAS to start c
onnection
ActivateEpsBearer (default)
tell MME to activate
bearer when UE con
nects
ActivateDedicatedEpsBearer
ActivateEpsBearer
tell MME to activate
bearer when UE con
nects
