# Supplementary material for: An empirical evaluation of link quality utilization in ETX routing for VANETs
Source: PeerJ Comput Sci. 2024 Sep 6;10:e2259. doi: 10.7717/peerj-cs.2259 (PMC11419655; doi:10.7717/peerj-cs.2259)
Supplement: Supplemental Information 1 [file peerj-cs-10-2259-s001.bz2 › ns-allinone-3.29/ns-3.29/src/lte/doc/source/figures/lte-ue-rrc-states.pdf]

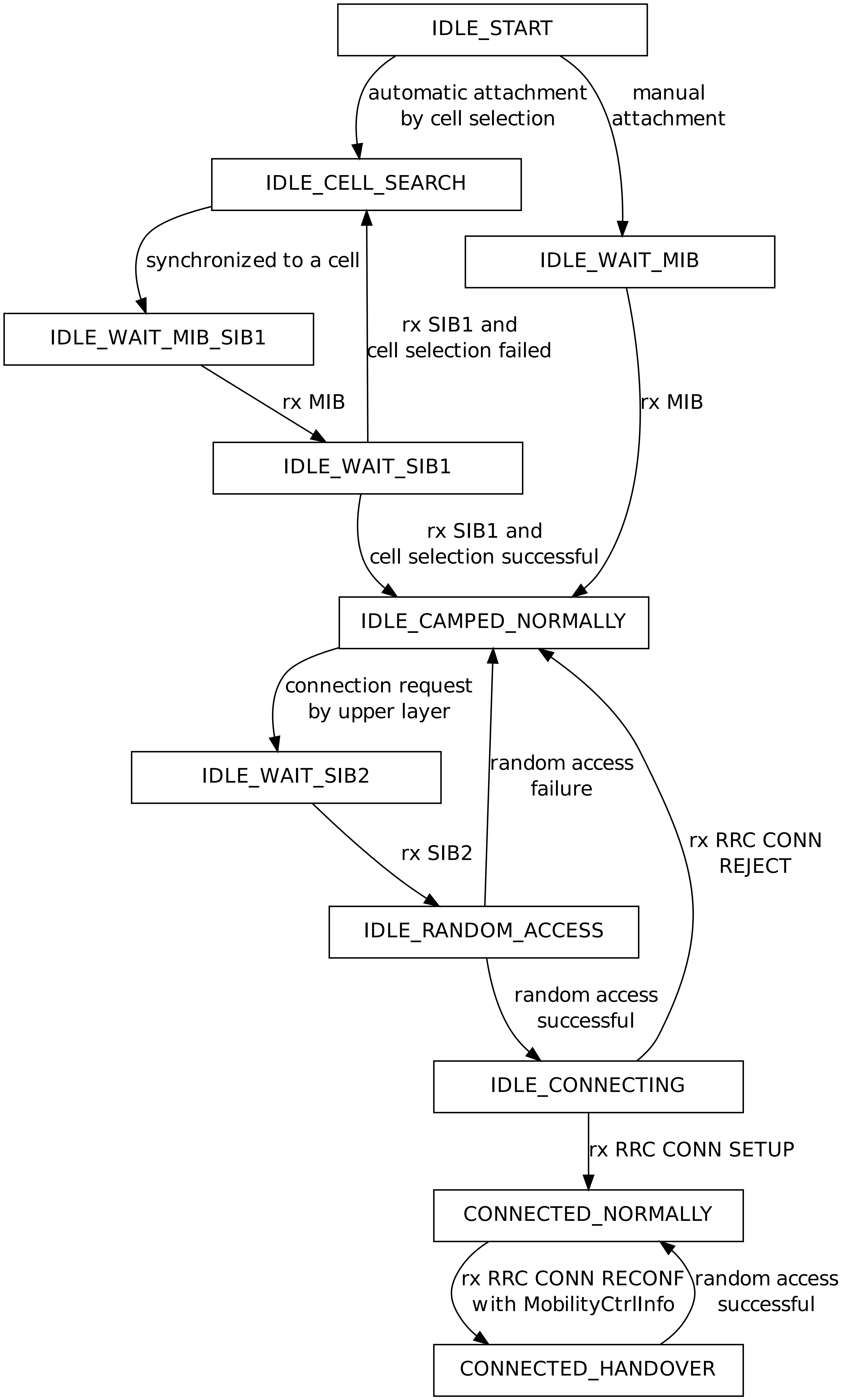
IDLE_START
automatic attachment
by cell selection
manual
attachment
IDLE_CELL_SEARCH
IDLE_WAIT_MIB
synchronized to a cell
IDLE_WAIT_MIB_SIB1
rx SIB1 and
cell selection failed
rx MIB
rx MIB
IDLE_WAIT_SIB1
rx SIB1 and
cell selection successful
IDLE_CAMPED_NORMALLY
connection request
by upper layer
IDLE_WAIT_SIB2
random access
failure
rx RRC CONN
REJECT
rx SIB2
IDLE_RANDOM_ACCESS
random access
successful
IDLE_CONNECTING
rx RRC CONN SETUP
CONNECTED_NORMALLY
rx RRC CONN RECONF
with MobilityCtrlInfo
random access
successful
CONNECTED_HANDOVER
