# Supplementary material for: An empirical evaluation of link quality utilization in ETX routing for VANETs
Source: PeerJ Comput Sci. 2024 Sep 6;10:e2259. doi: 10.7717/peerj-cs.2259 (PMC11419655; doi:10.7717/peerj-cs.2259)
Supplement: Supplemental Information 1 [file peerj-cs-10-2259-s001.bz2 › ns-allinone-3.29/ns-3.29/src/lte/doc/source/figures/lte-enb-rrc-states.pdf]

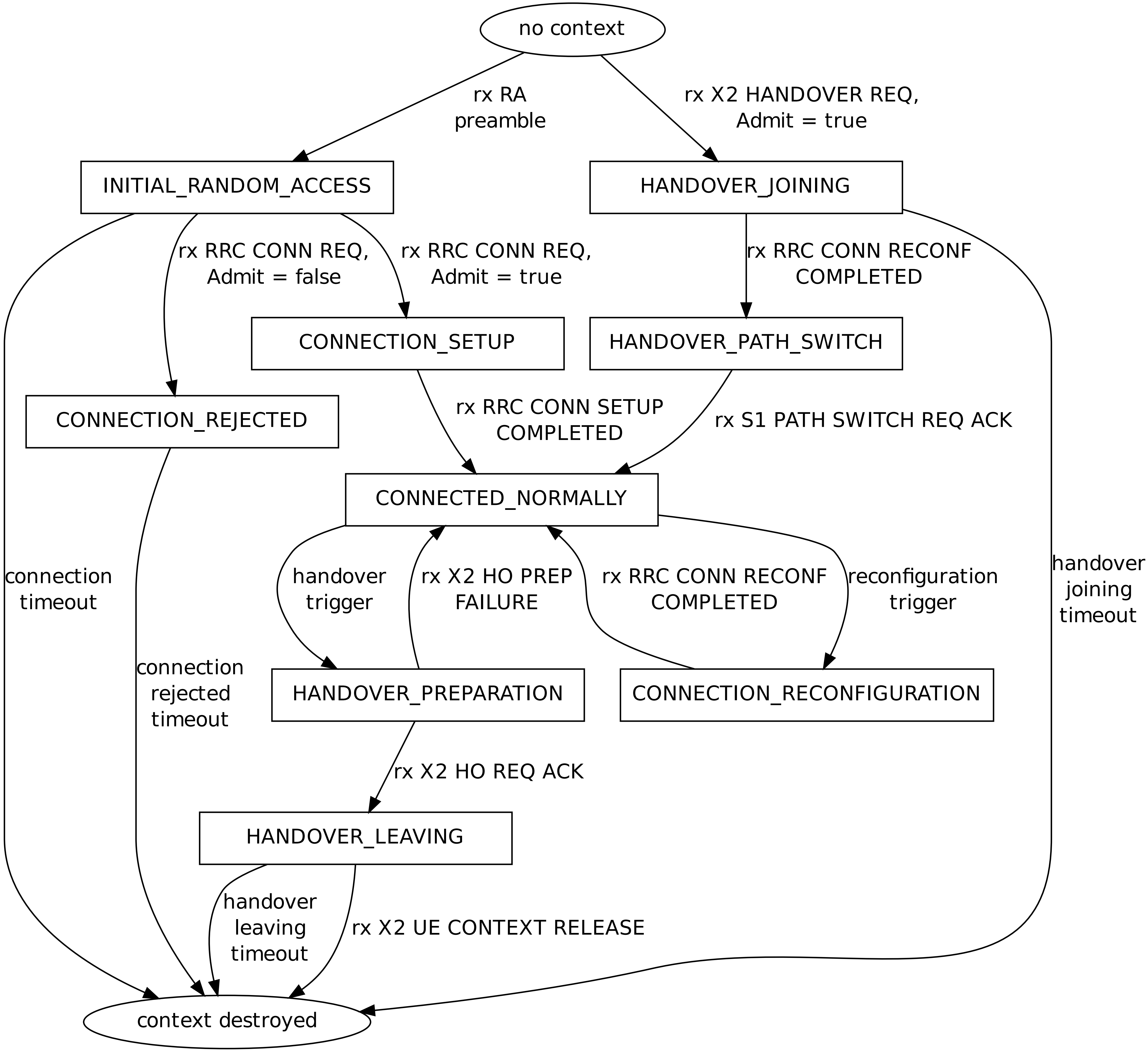

no context
rx RA preamble
rx X2 HANDOVER REQ, Admit = true
INITIAL_RANDOM_ACCESS
HANDOVER_JOINING
rx RRC CONN REQ, Admit = false
rx RRC CONN REQ, Admit = true
rx RRC CONN RECONF COMPLETED
CONNECTION_SETUP
HANDOVER_PATH_SWITCH
CONNECTION_REJECTED
rx RRC CONN SETUP COMPLETED
rx S1 PATH SWITCH REQ ACK
CONNECTED_NORMALLY
connection timeout
handover trigger
rx X2 HO PREP FAILURE
rx RRC CONN RECONF COMPLETED
reconfiguration trigger
handover joining timeout
connection rejected timeout
HANDOVER_PREPARATION
CONNECTION_RECONFIGURATION
rx X2 HO REQ ACK
HANDOVER_LEAVING
handover leaving timeout
rx X2 UE CONTEXT RELEASE
context destroyed
