# Supplementary material for: An empirical evaluation of link quality utilization in ETX routing for VANETs
Source: PeerJ Comput Sci. 2024 Sep 6;10:e2259. doi: 10.7717/peerj-cs.2259 (PMC11419655; doi:10.7717/peerj-cs.2259)
Supplement: Supplemental Information 1 [file peerj-cs-10-2259-s001.bz2 › ns-allinone-3.29/ns-3.29/src/lte/doc/source/figures/rrc-connection-reconfiguration.pdf]

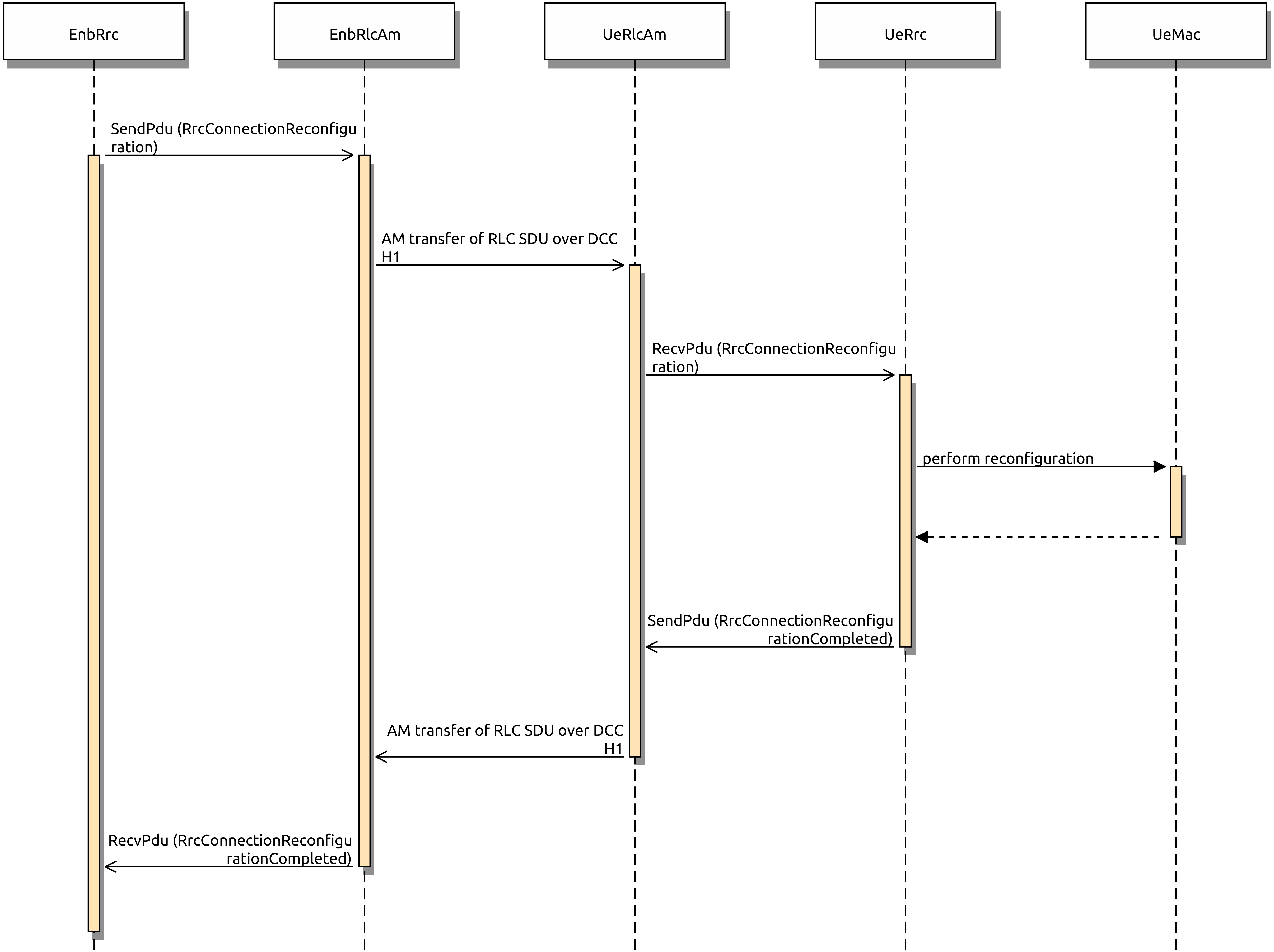
EnbRrc
EnbRlcAm
UeRlcAm
UeRrc
UeMac
SendPdu (RrcConnectionReconfiguration)
AM transfer of RLC SDU over DCCH1
RecvPdu (RrcConnectionReconfiguration)
perform reconfiguration
SendPdu (RrcConnectionReconfigurationCompleted)
AM transfer of RLC SDU over DCCH1
RecvPdu (RrcConnectionReconfigurationCompleted)
