# Supplementary material for: An empirical evaluation of link quality utilization in ETX routing for VANETs
Source: PeerJ Comput Sci. 2024 Sep 6;10:e2259. doi: 10.7717/peerj-cs.2259 (PMC11419655; doi:10.7717/peerj-cs.2259)
Supplement: Supplemental Information 1 [file peerj-cs-10-2259-s001.bz2 › ns-allinone-3.29/ns-3.29/src/lte/doc/source/figures/ca-downlink-bsr.pdf]

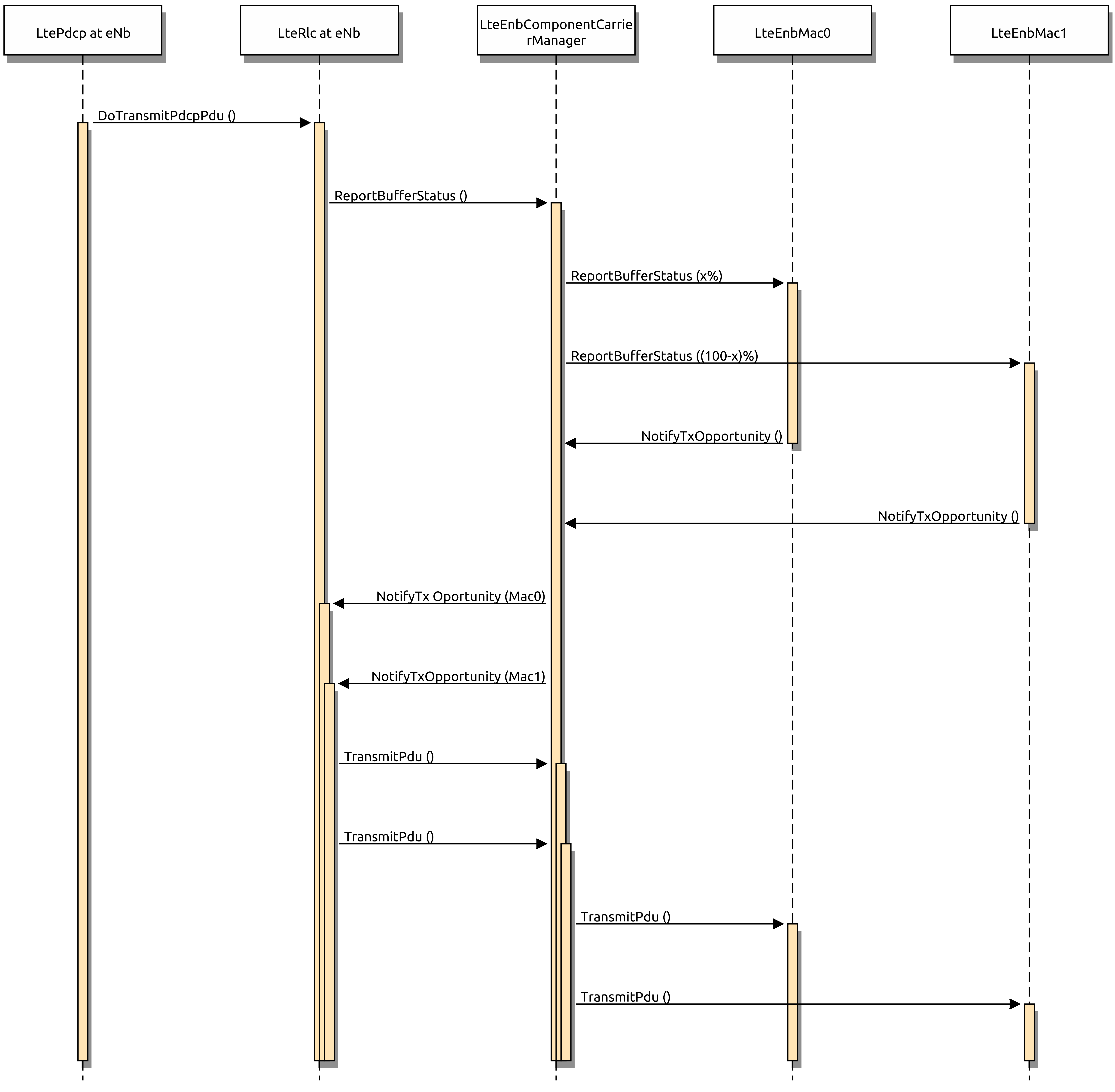
LtePdcp at eNb
LteRlc at eNb
LteEnbComponentCarrierManager
LteEnbMac0
LteEnbMac1
DoTransmitPdcpPdu ()
ReportBufferStatus ()
ReportBufferStatus (x%)
ReportBufferStatus ((100-x)%)
NotifyTxOpportunity ()
NotifyTxOpportunity ()
NotifyTx Oportunity (Mac0)
NotifyTxOpportunity (Mac1)
TransmitPdu ()
TransmitPdu ()
TransmitPdu ()
TransmitPdu ()
