# Supplementary material for: An empirical evaluation of link quality utilization in ETX routing for VANETs
Source: PeerJ Comput Sci. 2024 Sep 6;10:e2259. doi: 10.7717/peerj-cs.2259 (PMC11419655; doi:10.7717/peerj-cs.2259)
Supplement: Supplemental Information 1 [file peerj-cs-10-2259-s001.bz2 › ns-allinone-3.29/ns-3.29/src/lte/doc/source/figures/ca-setup-radio-bearer.pdf]

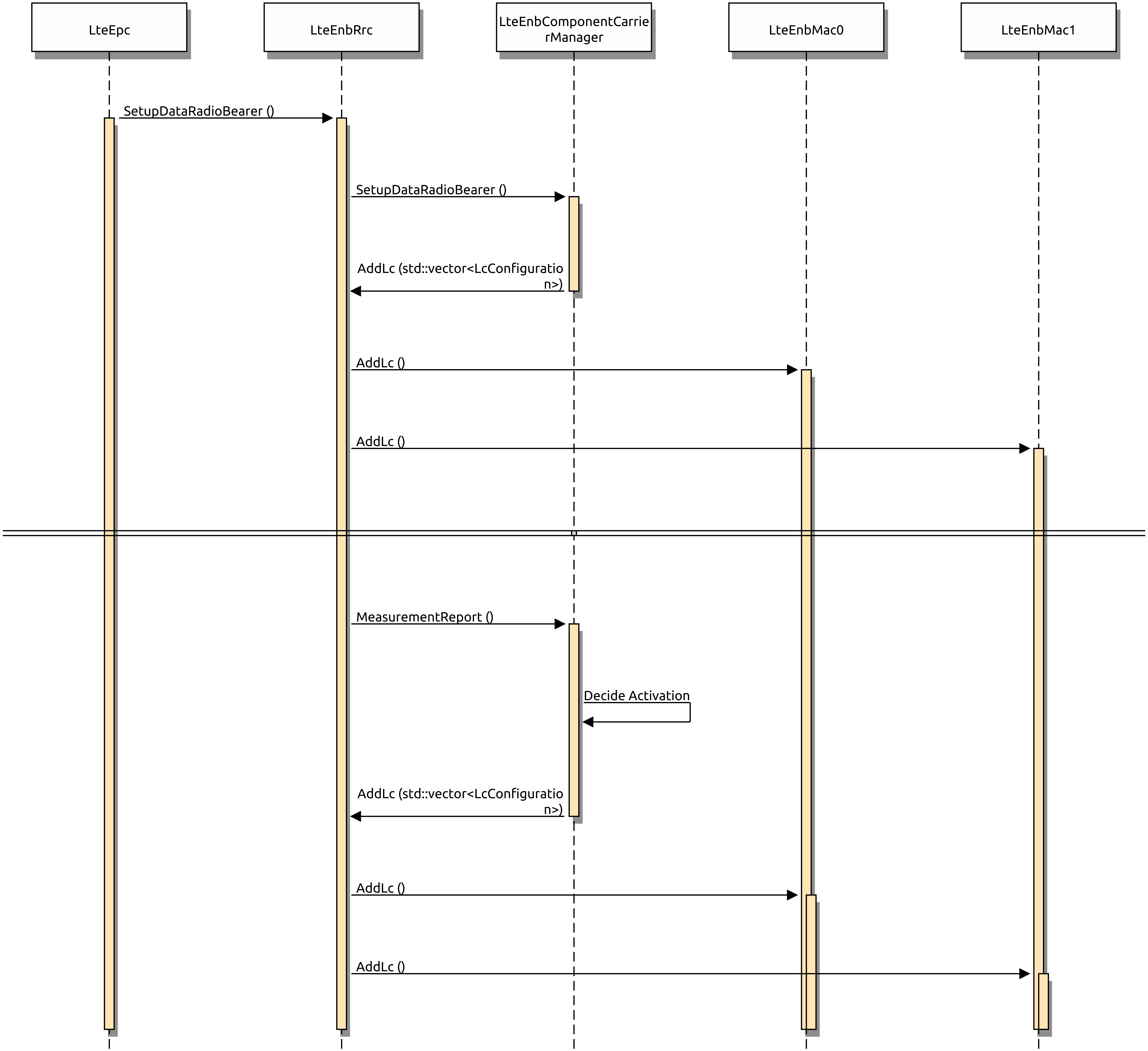

LteEpc
LteEnbRrc
LteEnbComponentCarrierManager
LteEnbMac0
LteEnbMac1
SetupDataRadioBearer ()
SetupDataRadioBearer ()
AddLc (std::vector<LcConfiguration>)
AddLc ()
AddLc ()
MeasurementReport ()
Decide Activation
AddLc (std::vector<LcConfiguration>)
AddLc ()
AddLc ()
