# Supplementary material for: An empirical evaluation of link quality utilization in ETX routing for VANETs
Source: PeerJ Comput Sci. 2024 Sep 6;10:e2259. doi: 10.7717/peerj-cs.2259 (PMC11419655; doi:10.7717/peerj-cs.2259)
Supplement: Supplemental Information 1 [file peerj-cs-10-2259-s001.bz2 › ns-allinone-3.29/ns-3.29/src/lte/doc/source/figures/lte-phy-interference.pdf]

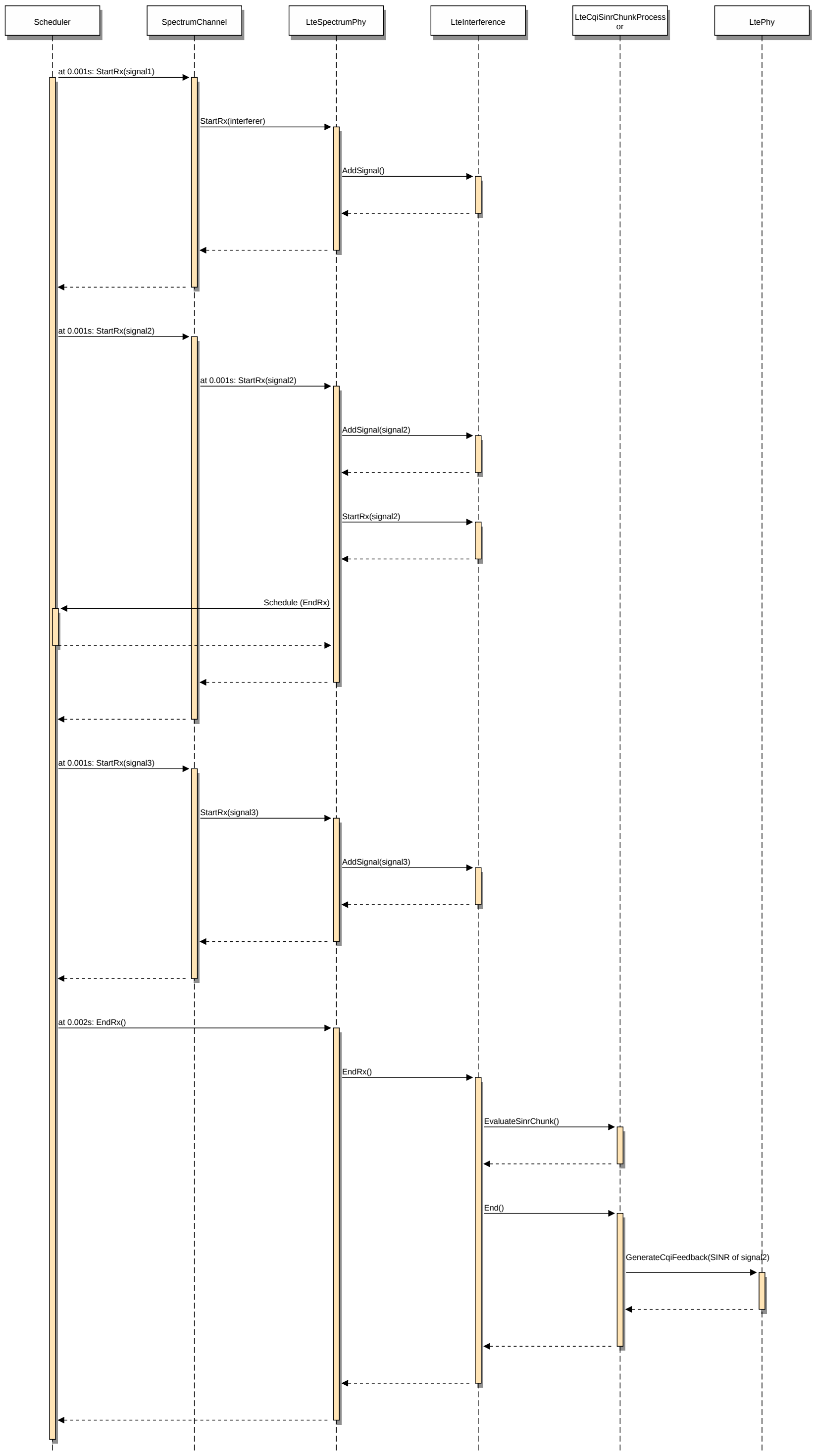
Scheduler
SpectrumChannel
LteSpectrumPhy
LteInterference
LteCqiSinrChunkProcessor
LtePhy
at 0.001s: StartRx(signal1)
StartRx(interferer)
AddSignal()
at 0.001s: StartRx(signal2)
at 0.001s: StartRx(signal2)
AddSignal(signal2)
StartRx(signal2)
Schedule (EndRx)
at 0.001s: StartRx(signal3)
StartRx(signal3)
AddSignal(signal3)
at 0.002s: EndRx()
EndRx()
EvaluateSinrChunk()
End()
GenerateCqiFeedback(SINR of signal2)
