# Supplementary material for: An empirical evaluation of link quality utilization in ETX routing for VANETs
Source: PeerJ Comput Sci. 2024 Sep 6;10:e2259. doi: 10.7717/peerj-cs.2259 (PMC11419655; doi:10.7717/peerj-cs.2259)
Supplement: Supplemental Information 1 [file peerj-cs-10-2259-s001.bz2 › ns-allinone-3.29/ns-3.29/src/lte/doc/source/figures/lte-handover-algorithm.pdf]

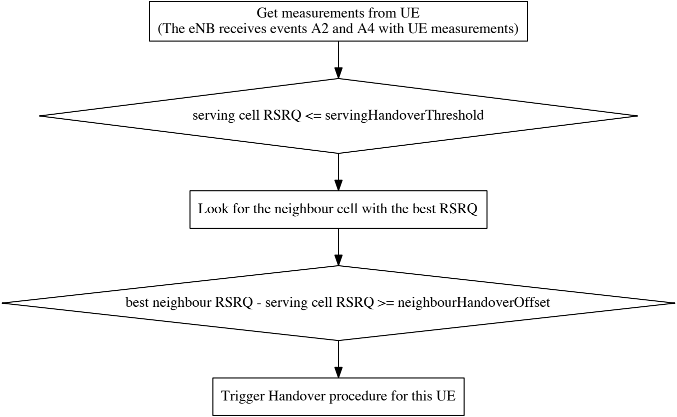
Get measurements from UE
(The eNB receives events A2 and A4 with UE measurements)
serving cell RSRQ <= servingHandoverThreshold
Look for the neighbour cell with the best RSRQ
best neighbour RSRQ - serving cell RSRQ >= neighbourHandoverOffset
Trigger Handover procedure for this UE
