# Supplementary material for: An empirical evaluation of link quality utilization in ETX routing for VANETs
Source: PeerJ Comput Sci. 2024 Sep 6;10:e2259. doi: 10.7717/peerj-cs.2259 (PMC11419655; doi:10.7717/peerj-cs.2259)
Supplement: Supplemental Information 1 [file peerj-cs-10-2259-s001.bz2 › ns-allinone-3.29/ns-3.29/src/lte/doc/source/figures/mac-random-access-noncontention.pdf]

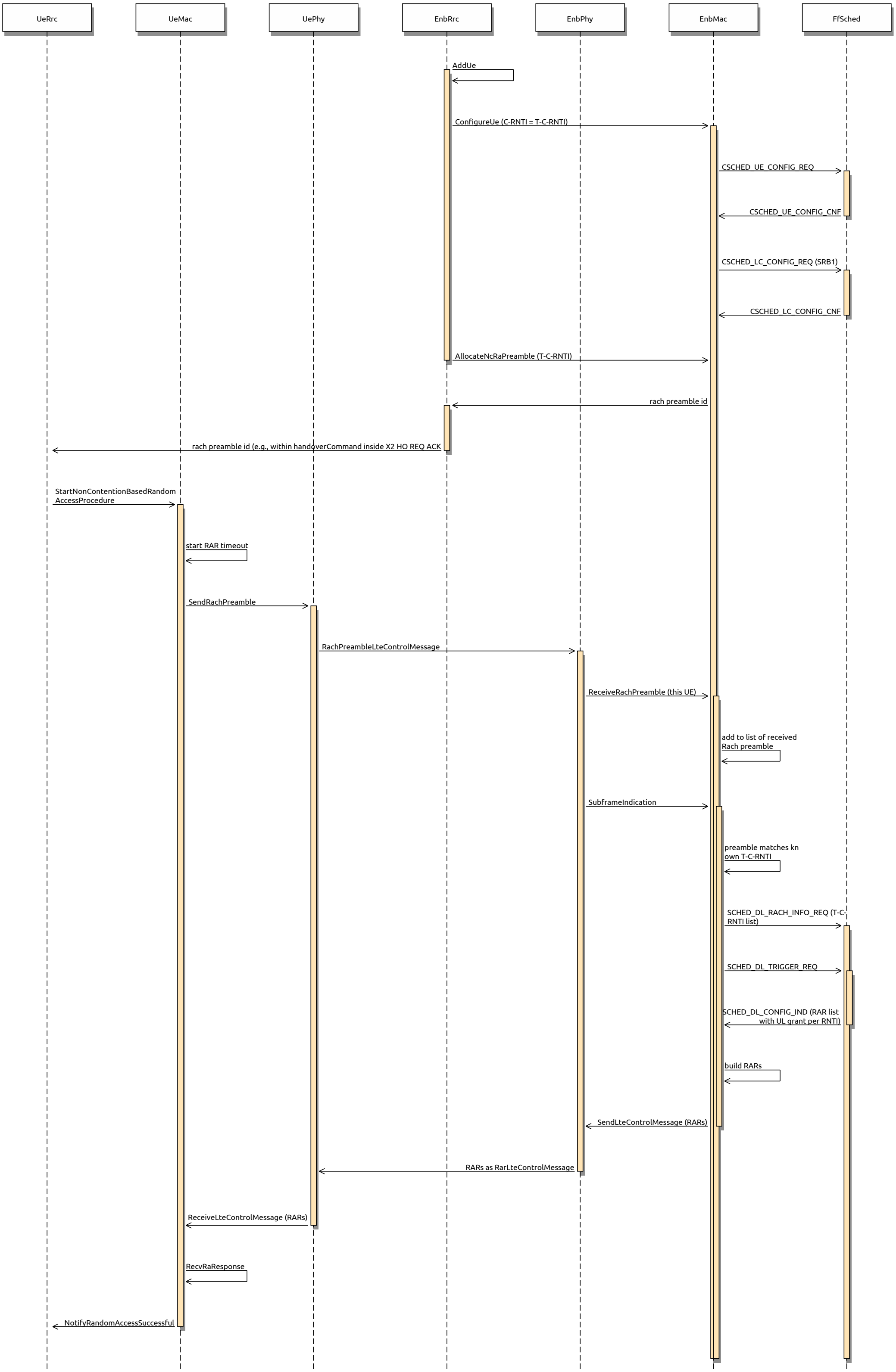

UeRrc
UeMac
UePhy
EnbRrc
EnbPhy
EnbMac
FfSched
AddUe
ConfigureUe (C-RNTI = T-C-RNTI)
CSCHED_UE_CONFIG_REQ
CSCHED_UE_CONFIG_CNF
CSCHED_LC_CONFIG_REQ (SRB1)
CSCHED_LC_CONFIG_CNF
AllocateNcRaPreamble (T-C-RNTI)
rach preamble id
rach preamble id (e.g., within handoverCommand inside X2 HO REQ ACK
StartNonContentionBasedRandom AccessProcedure
start RAR timeout
SendRachPreamble
RachPreambleLteControlMessage
ReceiveRachPreamble (this UE)
add to list of received Rach preamble
SubframeIndication
preamble matches kn own T-C-RNTI
SCHED_DL_RACH_INFO_REQ (T-C-RNTI list)
SCHED_DL_TRIGGER_REQ
SCHED_DL_CONFIG_IND (RAR list with UL grant per RNTI)
build RARs
SendLteControlMessage (RARs)
RARs as RarLteControlMessage
ReceiveLteControlMessage (RARs)
RecvRaResponse
NotifyRandomAccessSuccessful
