# Supplementary material for: An empirical evaluation of link quality utilization in ETX routing for VANETs
Source: PeerJ Comput Sci. 2024 Sep 6;10:e2259. doi: 10.7717/peerj-cs.2259 (PMC11419655; doi:10.7717/peerj-cs.2259)
Supplement: Supplemental Information 1 [file peerj-cs-10-2259-s001.bz2 › ns-allinone-3.29/ns-3.29/src/netanim/doc/figures/NodeCountersTable.pdf]

Counter Tables | Sim Time | Font Size 10 | FlowMon file | Double Counter 1

Export Table

	Time	0	1	2	3	4	5	6	7	8	9	10	11
1	0.1	134.532											
2	0.1						101.387						
3	0.2	164.6											
4	0.2						168.151						
5	0.3	154.444											
6	0.3						115.763						
7	0.4	179.264											
8	0.4						126.946						
9	0.5	144.204											
10	0.5						136.543						
11	0.6	116.857											
12	0.6						159.699						
13	0.7	174.138											
14	0.7						138.387						
15	0.8	101.934											
16	0.8						149.555						
17	0.9	195.25											
18	0.9						192.968						
19	1	132.337											
20	1						114.404						
21	1.1	142.992											
22	1.1						190.218						
23	1.2	122.185											
