# Supplementary material for: An empirical evaluation of link quality utilization in ETX routing for VANETs
Source: PeerJ Comput Sci. 2024 Sep 6;10:e2259. doi: 10.7717/peerj-cs.2259 (PMC11419655; doi:10.7717/peerj-cs.2259)
Supplement: Supplemental Information 1 [file peerj-cs-10-2259-s001.bz2 › ns-allinone-3.29/ns-3.29/src/netanim/doc/figures/PacketStatistics.pdf]

Packet count 11208

From Node Id 1

To Node Id 0

Transmission time >= 0

Apply filter

Select All

DeSelect All

- [ ] All Packets
- [ ] Ethernet
- [ ] Ppp
- [ ] Wifi
- [ ] Arp
- [ ] Ipv4
- [ ] Icmpv4
- [ ] Udp
- [x] Tcp
- [ ] Aodv
- [ ] Olsr
- [ ] Dsdv

	Tx Time	From Node Id	To Node Id	Meta Info
1	0.0301008	1	0	TCP 50000 > 49153 SYN ACK Seq=0 Ack=1 Win=65535
2	0.0711264	1	0	TCP 50000 > 49153 ACK Seq=1 Ack=537 Win=65535
3	0.112581	1	0	TCP 50000 > 49153 ACK Seq=1 Ack=1609 Win=65535
4	0.154035	1	0	TCP 50000 > 49153 ACK Seq=1 Ack=2681 Win=65535
5	0.195027	1	0	TCP 50000 > 49153 ACK Seq=1 Ack=3753 Win=65535
6	0.195952	1	0	TCP 50000 > 49153 ACK Seq=1 Ack=4825 Win=65535
7	0.236482	1	0	TCP 50000 > 49153 ACK Seq=1 Ack=5897 Win=65535
8	0.237406	1	0	TCP 50000 > 49153 ACK Seq=1 Ack=6969 Win=65535
9	0.238331	1	0	TCP 50000 > 49153 ACK Seq=1 Ack=8041 Win=65535
10	0.277936	1	0	TCP 50000 > 49153 ACK Seq=1 Ack=9113 Win=65535
11	0.278861	1	0	TCP 50000 > 49153 ACK Seq=1 Ack=10185 Win=65535
12	0.279786	1	0	TCP 50000 > 49153 ACK Seq=1 Ack=11257 Win=65535
13	0.28071	1	0	TCP 50000 > 49153 ACK Seq=1 Ack=12329 Win=65535
14	0.318928	1	0	TCP 50000 > 49153 ACK Seq=1 Ack=13401 Win=65535
15	0.319853	1	0	TCP 50000 > 49153 ACK Seq=1 Ack=14473 Win=65535
16	0.320778	1	0	TCP 50000 > 49153 ACK Seq=1 Ack=15545 Win=65535
17	0.321702	1	0	TCP 50000 > 49153 ACK Seq=1 Ack=16617 Win=65535
18	0.322627	1	0	TCP 50000 > 49153 ACK Seq=1 Ack=17689 Win=65535
19	0.323552	1	0	TCP 50000 > 49153 ACK Seq=1 Ack=18761 Win=65535
20	0.35992	1	0	TCP 50000 > 49153 ACK Seq=1 Ack=19833 Win=65535
21	0.360845	1	0	TCP 50000 > 49153 ACK Seq=1 Ack=20905 Win=65535
22	0.36177	1	0	TCP 50000 > 49153 ACK Seq=1 Ack=21977 Win=65535
23	0.362694	1	0	TCP 50000 > 49153 ACK Seq=1 Ack=23049 Win=65535
