# Supplementary material for: An empirical evaluation of link quality utilization in ETX routing for VANETs
Source: PeerJ Comput Sci. 2024 Sep 6;10:e2259. doi: 10.7717/peerj-cs.2259 (PMC11419655; doi:10.7717/peerj-cs.2259)
Supplement: Supplemental Information 1 [file peerj-cs-10-2259-s001.bz2 › ns-allinone-3.29/ns-3.29/src/netanim/doc/figures/NetAnim_3_105.pdf]

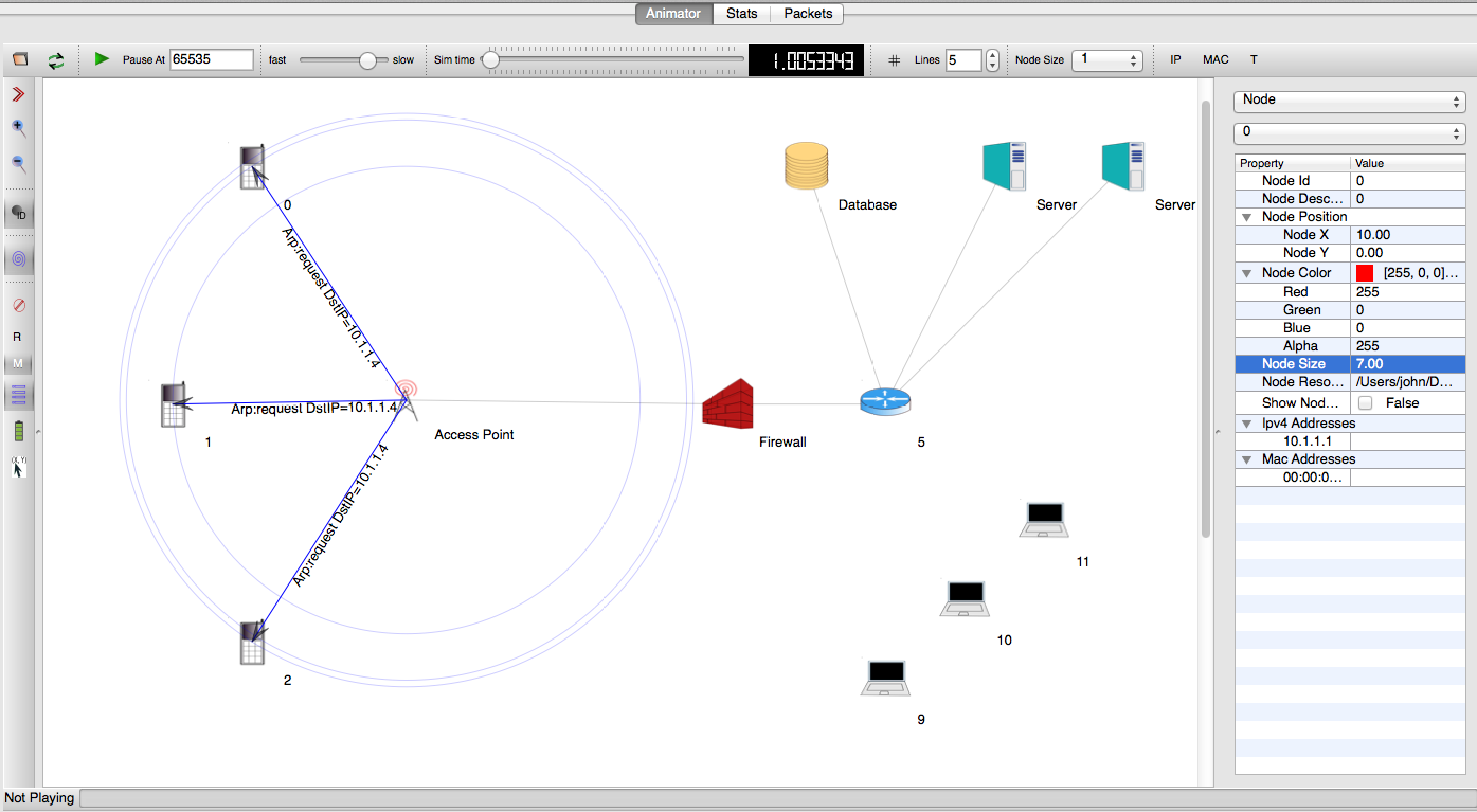
Animator
Stats
Packets
Pause At
65535
fast
slow
Sim time
1.0053349
Lines
5
Node Size
1
IP
MAC
T
R
M
Arp:request DstIP=10.1.1.4
Arp:request DstIP=10.1.1.4
Arp:request DstIP=10.1.1.4
0
1
2
Access Point
Firewall
5
Database
Server
Server
11
10
9
Node
0
Property
Value
Node Id
0
Node Desc...
0
Node Position
Node X
10.00
Node Y
0.00
Node Color
[255, 0, 0]...
Red
255
Green
0
Blue
0
Alpha
255
Node Size
7.00
Node Reso...
/Users/john/D...
Show Nod...
False
Ipv4 Addresses
10.1.1.1
Mac Addresses
00:00:0...
Not Playing
