# Supplementary material for: An empirical evaluation of link quality utilization in ETX routing for VANETs
Source: PeerJ Comput Sci. 2024 Sep 6;10:e2259. doi: 10.7717/peerj-cs.2259 (PMC11419655; doi:10.7717/peerj-cs.2259)
Supplement: Supplemental Information 1 [file peerj-cs-10-2259-s001.bz2 › ns-allinone-3.29/ns-3.29/src/netanim/doc/figures/PacketTimeline.pdf]

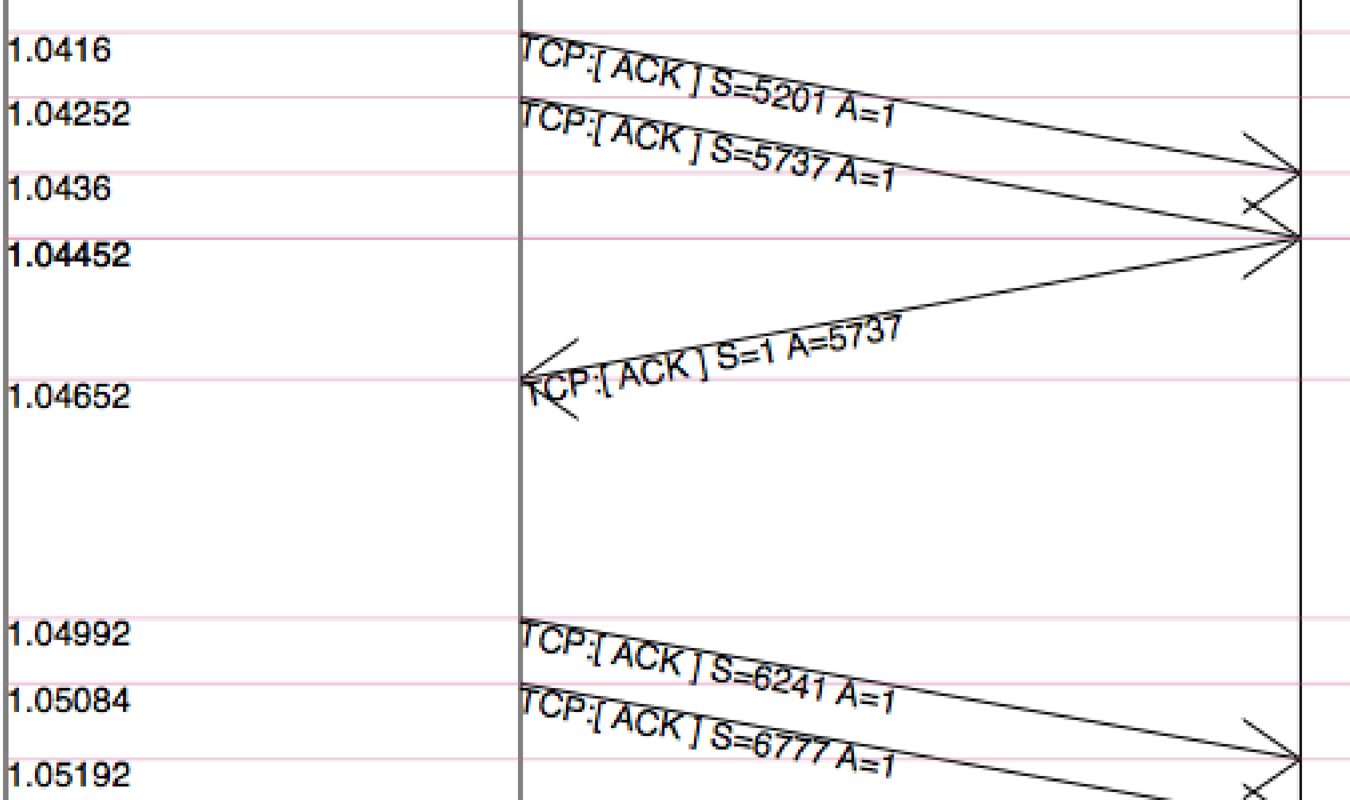
1.0416
1.04252
1.0436
1.04452
1.04652
1.04992
1.05084
1.05192
TCP:[ACK] S=5201 A=1
TCP:[ACK] S=5737 A=1
TCP:[ACK] S=1 A=5737
TCP:[ACK] S=6241 A=1
TCP:[ACK] S=6777 A=1
