# Supplementary material for: An empirical evaluation of link quality utilization in ETX routing for VANETs
Source: PeerJ Comput Sci. 2024 Sep 6;10:e2259. doi: 10.7717/peerj-cs.2259 (PMC11419655; doi:10.7717/peerj-cs.2259)
Supplement: Supplemental Information 1 [file peerj-cs-10-2259-s001.bz2 › ns-allinone-3.29/ns-3.29/src/netanim/doc/figures/RoutingTables.pdf]

```
Node: 0 Time: 0s Ipv4ListRouting table
  Priority: 0 Protocol: ns3::Ipv4StaticRouting
Destination   Gateway    Genmask          Flags Metric Ref   Use Iface
127.0.0.0     0.0.0.0    255.0.0.0        U     0      -     -   0
10.1.3.0      0.0.0.0    255.255.255.0    U     0      -     -   1
  Priority: -10 Protocol: ns3::Ipv4GlobalRouting
Destination   Gateway    Genmask          Flags Metric Ref   Use Iface
10.1.1.1      10.1.3.21  255.255.255.255  UH    -      -     -   1
10.1.1.2      10.1.3.21  255.255.255.255  UH    -      -     -   1
10.1.3.0      0.0.0.0    255.255.255.0    U     -      -     -   1
10.1.2.0      10.1.3.21  255.255.255.0    UG    -      -     -   1
127.0.0.0     10.1.3.2   255.0.0.0        UG    -      -     -   1
127.0.0.0     10.1.3.3   255.0.0.0        UG    -      -     -   1
127.0.0.0     10.1.3.4   255.0.0.0        UG    -      -     -   1
127.0.0.0     10.1.3.5   255.0.0.0        UG    -      -     -   1
127.0.0.0     10.1.3.6   255.0.0.0        UG    -      -     -   1
127.0.0.0     10.1.3.7   255.0.0.0        UG    -      -     -   1
127.0.0.0     10.1.3.8   255.0.0.0        UG    -      -     -   1
127.0.0.0     10.1.3.9   255.0.0.0        UG    -      -     -   1
127.0.0.0     10.1.3.10  255.0.0.0        UG    -      -     -   1
127.0.0.0     10.1.3.11  255.0.0.0        UG    -      -     -   1
127.0.0.0     10.1.3.12  255.0.0.0        UG    -      -     -   1
127.0.0.0     10.1.3.13  255.0.0.0        UG    -      -     -   1
127.0.0.0     10.1.3.14  255.0.0.0        UG    -      -     -   1
127.0.0.0     10.1.3.15  255.0.0.0        UG    -      -     -   1
127.0.0.0     10.1.3.16  255.0.0.0        UG    -      -     -   1
127.0.0.0     10.1.3.17  255.0.0.0        UG    -      -     -   1
127.0.0.0     10.1.3.18  255.0.0.0        UG    -      -     -   1
127.0.0.0     10.1.3.19  255.0.0.0        UG    -      -     -   1
127.0.0.0     10.1.3.20  255.0.0.0        UG    -      -     -   1
10.1.1.0      10.1.3.21  255.255.255.0    UG    -      -     -   1
127.0.0.0     10.1.3.21  255.0.0.0        UG    -      -     -   1
10.1.1.0      10.1.3.21  255.255.255.0    UG    -      -     -   1
127.0.0.0     10.1.3.21  255.0.0.0        UG    -      -     -   1
127.0.0.0     10.1.3.21  255.0.0.0        UG    -      -     -   1
```

```
Node: 1 Time: 0s Ipv4ListRouting table
  Priority: 0 Protocol: ns3::Ipv4StaticRouting
Destination   Gateway    Genmask          Flags Metric Ref   Use Iface
127.0.0.0     0.0.0.0    255.0.0.0        U     0      -     -   0
10.1.3.0      0.0.0.0    255.255.255.0    U     0      -     -   1
  Priority: -10 Protocol: ns3::Ipv4GlobalRouting
Destination   Gateway    Genmask          Flags Metric Ref   Use Iface
10.1.1.1      10.1.3.21  255.255.255.255  UH    -      -     -   1
10.1.1.2      10.1.3.21  255.255.255.255  UH    -      -     -   1
10.1.3.0      0.0.0.0    255.255.255.0    U     -      -     -   1
10.1.2.0      10.1.3.21  255.255.255.0    UG    -      -     -   1
127.0.0.0     10.1.3.1   255.0.0.0        UG    -      -     -   1
127.0.0.0     10.1.3.3   255.0.0.0        UG    -      -     -   1
127.0.0.0     10.1.3.4   255.0.0.0        UG    -      -     -   1
127.0.0.0     10.1.3.5   255.0.0.0        UG    -      -     -   1
127.0.0.0     10.1.3.6   255.0.0.0        UG    -      -     -   1
127.0.0.0     10.1.3.7   255.0.0.0        UG    -      -     -   1
127.0.0.0     10.1.3.8   255.0.0.0        UG    -      -     -   1
127.0.0.0     10.1.3.9   255.0.0.0        UG    -      -     -   1
127.0.0.0     10.1.3.10  255.0.0.0        UG    -      -     -   1
127.0.0.0     10.1.3.11  255.0.0.0        UG    -      -     -   1
127.0.0.0     10.1.3.12  255.0.0.0        UG    -      -     -   1
127.0.0.0     10.1.3.13  255.0.0.0        UG    -      -     -   1
127.0.0.0     10.1.3.14  255.0.0.0        UG    -      -     -   1
127.0.0.0     10.1.3.15  255.0.0.0        UG    -      -     -   1
127.0.0.0     10.1.3.16  255.0.0.0        UG    -      -     -   1
127.0.0.0     10.1.3.17  255.0.0.0        UG    -      -     -   1
127.0.0.0     10.1.3.18  255.0.0.0        UG    -      -     -   1
127.0.0.0     10.1.3.19  255.0.0.0        UG    -      -     -   1
127.0.0.0     10.1.3.20  255.0.0.0        UG    -      -     -   1
10.1.1.0      10.1.3.21  255.255.255.0    UG    -      -     -   1
127.0.0.0     10.1.3.21  255.0.0.0        UG    -      -     -   1
10.1.1.0      10.1.3.21  255.255.255.0    UG    -      -     -   1
127.0.0.0     10.1.3.21  255.0.0.0        UG    -      -     -   1
127.0.0.0     10.1.3.21  255.0.0.0        UG    -      -     -   1
```

```
Node: 2 Time: 0s Ipv4ListRouting table
  Priority: 0 Protocol: ns3::Ipv4StaticRouting
Destination   Gateway    Genmask          Flags Metric Ref   Use Iface
127.0.0.0     0.0.0.0    255.0.0.0        U     0      -     -   0
10.1.3.0      0.0.0.0    255.255.255.0    U     0      -     -   1
  Priority: -10 Protocol: ns3::Ipv4GlobalRouting
Destination   Gateway    Genmask          Flags Metric Ref   Use Iface
10.1.1.1      10.1.3.21  255.255.255.255  UH    -      -     -   1
10.1.1.2      10.1.3.21  255.255.255.255  UH    -      -     -   1
10.1.3.0      0.0.0.0    255.255.255.0    U     -      -     -   1
10.1.2.0      10.1.3.21  255.255.255.0    UG    -      -     -   1
127.0.0.0     10.1.3.1   255.0.0.0        UG    -      -     -   1
127.0.0.0     10.1.3.2   255.0.0.0        UG    -      -     -   1
127.0.0.0     10.1.3.4   255.0.0.0        UG    -      -     -   1
127.0.0.0     10.1.3.5   255.0.0.0        UG    -      -     -   1
127.0.0.0     10.1.3.6   255.0.0.0        UG    -      -     -   1
127.0.0.0     10.1.3.7   255.0.0.0        UG    -      -     -   1
127.0.0.0     10.1.3.8   255.0.0.0        UG    -      -     -   1
127.0.0.0     10.1.3.9   255.0.0.0        UG    -      -     -   1
127.0.0.0     10.1.3.10  255.0.0.0        UG    -      -     -   1
127.0.0.0     10.1.3.11  255.0.0.0        UG    -      -     -   1
127.0.0.0     10.1.3.12  255.0.0.0        UG    -      -     -   1
127.0.0.0     10.1.3.13  255.0.0.0        UG    -      -     -   1
127.0.0.0     10.1.3.14  255.0.0.0        UG    -      -     -   1
127.0.0.0     10.1.3.15  255.0.0.0        UG    -      -     -   1
127.0.0.0     10.1.3.16  255.0.0.0        UG    -      -     -   1
127.0.0.0     10.1.3.17  255.0.0.0        UG    -      -     -   1
127.0.0.0     10.1.3.18  255.0.0.0        UG    -      -     -   1
127.0.0.0     10.1.3.19  255.0.0.0        UG    -      -     -   1
127.0.0.0     10.1.3.20  255.0.0.0        UG    -      -     -   1
10.1.1.0      10.1.3.21  255.255.255.0    UG    -      -     -   1
127.0.0.0     10.1.3.21  255.0.0.0        UG    -      -     -   1
10.1.1.0      10.1.3.21  255.255.255.0    UG    -      -     -   1
127.0.0.0     10.1.3.21  255.0.0.0        UG    -      -     -   1
127.0.0.0     10.1.3.21  255.0.0.0        UG    -      -     -   1
```
