# Supplementary material for: An empirical evaluation of link quality utilization in ETX routing for VANETs
Source: PeerJ Comput Sci. 2024 Sep 6;10:e2259. doi: 10.7717/peerj-cs.2259 (PMC11419655; doi:10.7717/peerj-cs.2259)
Supplement: Supplemental Information 1 [file peerj-cs-10-2259-s001.bz2 › ns-allinone-3.29/ns-3.29/src/netanim/doc/figures/Trajectory.pdf]

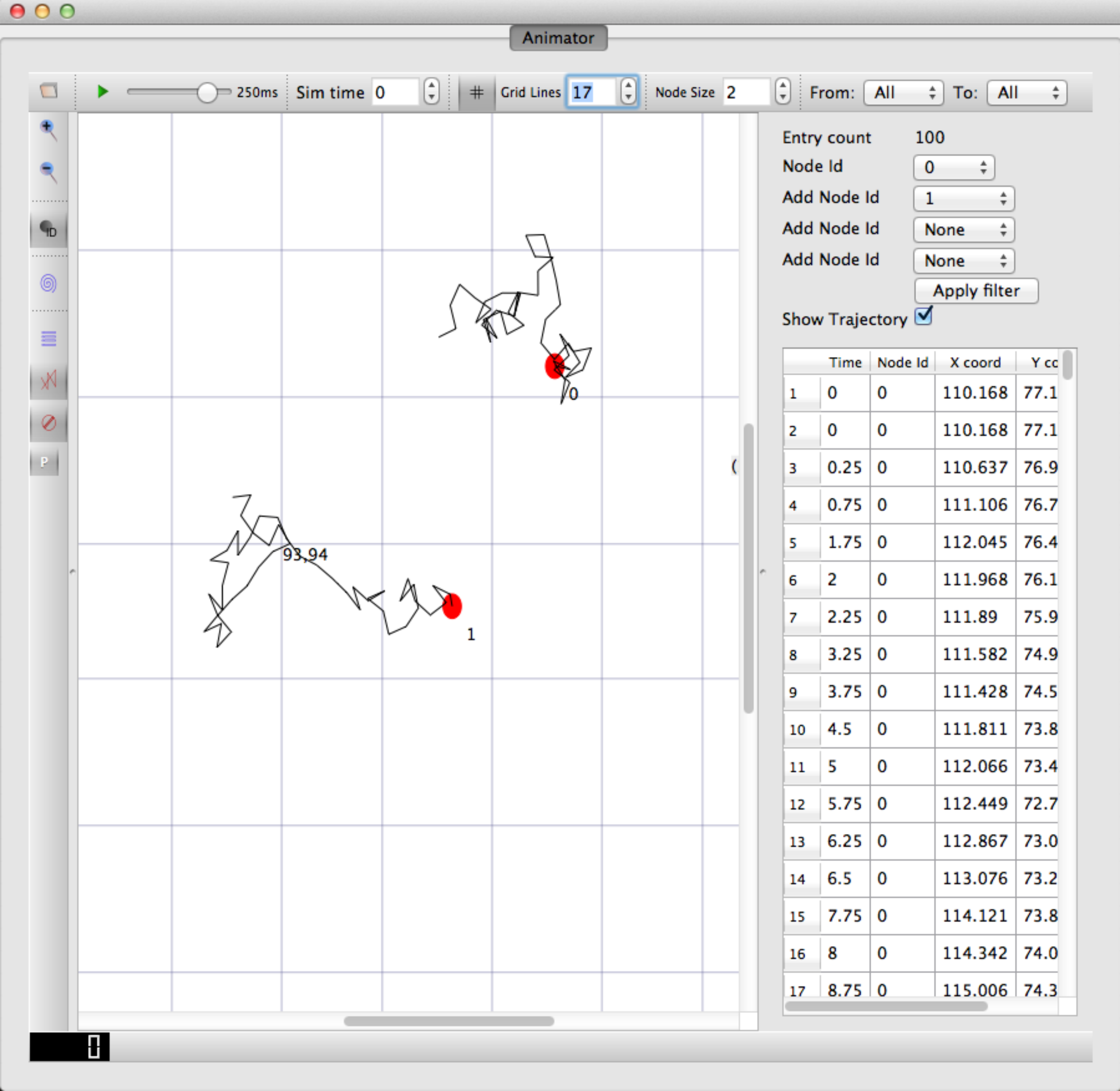

	Time	Node Id	X coord	Y co
1	0	0	110.168	77.1
2	0	0	110.168	77.1
3	0.25	0	110.637	76.9
4	0.75	0	111.106	76.7
5	1.75	0	112.045	76.4
6	2	0	111.968	76.1
7	2.25	0	111.89	75.9
8	3.25	0	111.582	74.9
9	3.75	0	111.428	74.5
10	4.5	0	111.811	73.8
11	5	0	112.066	73.4
12	5.75	0	112.449	72.7
13	6.25	0	112.867	73.0
14	6.5	0	113.076	73.2
15	7.75	0	114.121	73.8
16	8	0	114.342	74.0
17	8.75	0	115.006	74.3
