# Supplementary material for: An empirical evaluation of link quality utilization in ETX routing for VANETs
Source: PeerJ Comput Sci. 2024 Sep 6;10:e2259. doi: 10.7717/peerj-cs.2259 (PMC11419655; doi:10.7717/peerj-cs.2259)
Supplement: Supplemental Information 1 [file peerj-cs-10-2259-s001.bz2 › ns-allinone-3.29/ns-3.29/doc/ns3_html_theme/layout.html]

{#
ns-3/layout.html
~~~~~~~~~~~~~~~~~~~
Sphinx layout template for ns-3.
:copyright: Copyright 2012 by ns-3, see AUTHORS.
:license: GPL, see LICENSE for details.
#}
{% extends "basic/layout.html" %}
{% set reldelim1 = ' ' %}
{# set reldelim1 = ' @' #}
{%- block extrahead %}
{%- if theme\_customstylesheet %}
{%- endif %}
{%- if theme\_favicon %}
{%- endif %}
{% endblock %}
{% block header %}

|  |  |  |  |
| --- | --- | --- | --- |
|  | A Discrete-Event Network Simulator | - Home - Tutorials  ▼ English    Portuguese - Docs    ▼ Wiki    Manual    Models - Develop ▼ API    Bugs | {{ shorttitle }} |

{% endblock %}
{% block rootrellink %}- {{ theme\_projectname }}
{{ super() }}
{% endblock %}
{% if theme\_collapsiblesidebar|tobool %}
{% set script\_files = script\_files + ['\_static/sidebar.js'] %}
{% endif %}
