# Supplementary material for: An empirical evaluation of link quality utilization in ETX routing for VANETs
Source: PeerJ Comput Sci. 2024 Sep 6;10:e2259. doi: 10.7717/peerj-cs.2259 (PMC11419655; doi:10.7717/peerj-cs.2259)
Supplement: Supplemental Information 1 [file peerj-cs-10-2259-s001.bz2 › ns-allinone-3.29/ns-3.29/doc/ns3_html_theme/ns3_doxy_header.html]

$projectname: $title
$title

$treeview
$search
$mathjax
$extrastylesheet


|  |  |  |  |
| --- | --- | --- | --- |
|  | A Discrete-Event Network Simulator | - Home - Tutorials  ▼ English    Portuguese - Docs    ▼ Wiki    Manual    Models - Develop ▼ API    Bugs | API |
